# Systems biology analysis of vasodynamics in mouse cerebral arterioles during resting state and functional hyperemia

**DOI:** 10.1371/journal.pcbi.1013113

**Published:** 2026-04-27

**Authors:** Hadi Esfandi, Mahshad Javidan, Eric R. McGregor, Rozalyn M. Anderson, Ramin Pashaie

**Affiliations:** 1 Department of Electrical Engineering and Computer Science, Florida Atlantic University, Boca Raton, Florida, United States of America; 2 Department of Medicine, University of Wisconsin-Madison, Madison, Wisconsin, United States of America; 3 Geriatric Research, Education, and Clinical Center, William S. Middleton Memorial Veterans Hospital, Madison, Wisconsin, United States of America; Inria, FRANCE

## Abstract

Cerebral hemodynamics is tightly regulated by arteriolar vasodynamics. In this study, a systems biology approach was employed to investigate how the interplay between passive, myogenic, neurogenic, and astrocytic responses shapes arteriolar vasodynamics in small rodents. A model of neurovascular coupling is proposed in which neurons inhibit and dampen the myogenic response to promote vasodilation during activation, and facilitate the myogenic response to promote rapid vasoconstriction immediately post-activation. In this model, inhibition of the myogenic response is mediated by the hyperpolarization of smooth muscle and endothelial cells. Dampening and facilitation of the response are mediated by neuronal production of nitric oxide and release of neuropeptide Y, respectively. We also introduce a model for gliovascular coupling, in which astrocytes periodically inhibit the myogenic response upon detecting an increase in myogenic activity through interactions between their endfeet and arterioles. Our simulations suggest that in the resting state, delays in myogenic autoregulation can intrinsically generate low-frequency (∼0.1 Hz) oscillations in vessel diameter (vasomotion), in the absence of extrinsic neurogenic or systemic rhythmic inputs. In the active state, these oscillations are disrupted by the neurogenic and astrocytic responses. The biophysical model of arteriolar vasodynamics presented in this study lays the foundation for quantitative analysis of cerebral hemodynamics for cerebrovascular health diagnostics and hemodynamic neuroimaging.

## Author summary

Cerebral hemodynamic imaging is widely used to investigate brain function in-vivo. These signals are primarily shaped by arteriolar vasodynamics, which result from a combination of physiological processes mediated by multiple interacting cell types. A biophysical model of this dynamics offers a valuable computational framework for achieving more accurate and quantitative interpretation of hemodynamic signals. In this study, I applied a computational biology approach to incorporate several well-established cellular signaling pathways into a unified model, which was used to investigate system-level arteriolar behavior and identify missing or less understood mechanisms involved in cerebral blood flow regulation. Our results show that arteriolar vasodynamics is not solely driven by neurogenic responses; astrocytic response and hemo-vascular interactions also play important roles in shaping the observed dynamics. The model also provided a means to explore how in-silico analysis of hemodynamic signals can reveal potential cellular-level impairments that manifest as system-level changes in cerebral hemodynamics. Incorporating our proposed biophysical model into cerebral hemodynamic analysis can improve the fidelity of hemodynamic imaging—enabling more accurate inference of regional neuronal and astrocytic activity from hemodynamic signals, and enhancing our ability to diagnose cerebrovascular pathologies.

## Introduction

Cerebral arteriolar vasodynamics regulates essential mechanisms of brain homeostasis, including oxygen and glucose delivery [1], neuronal function [2,3], and glymphatic clearance [4]. Over the past two decades, advances in in-vivo imaging [5–8], genetic engineering [9], in-vitro vascular preparations [10,11], and targeted cellular manipulation [12] have enriched our understanding of the cellular mechanisms underlying cerebral blood flow (CBF) regulation. Several biophysical models have been developed to elucidate specific aspects of cerebrovascular dynamics, including cerebral autoregulation [13–15], vasomotion [16,17], neurovascular coupling (NVC) [18,19], and gliovascular coupling (GVC) [20,21]. Despite these advances, a biophysically grounded framework capable of systematically integrating myogenic, neurogenic, and astrocytic mechanisms that govern cerebrovascular dynamics remains lacking. In this article, we employed a computational and systems biology approach to study the dominant cell signaling pathways shaping arteriolar vasodynamics.

Functional hyperemia (FH) is a physiological process that boosts local blood flow in response to a local increase in brain activity. This increase in blood flow is facilitated by the transient vasodilation of feeding vessels mediated by the neurogliovascular coupling (NGVC), and deformability of red blood cells (RBCs) within the brain’s vasculature [22]. We can categorize these interactions into two groups: feedforward response, and feedback response [23]. The feedforward response refers to cerebral blood flow (CBF) regulatory mechanisms where non-vascular cells, in proportion to their physiological demand, send signals to vascular cells to modulate the myogenic response and induce vasodilation. If the vasodilations induced by feedforward responses fail to meet the demand during phases of intense and sustained activities, the feedback response, including the continuous feedback from metabolic deficiency sensors, is activated.

Research has demonstrated that RBCs act as oxygen sensors. RBCs autonomously regulate their deformability to modulate blood viscosity in capillaries in response to decreases in environmental oxygen tension [22]. We categorized the contribution of RBCs to FH as a feedback response, assuming their influence becomes significant only when vasodilation induced by feedforward NGVC cannot fully compensate for the oxygen consumed by activated brain cells. Moreover, biphasic vasodilations have been observed in the transitional zone (TZ) or pre-capillary arterioles during sustained FH, with the second delayed phase abolished by a K_*ATP*_ channel blocker [24]. Based on these findings, we also categorized K_*ATP*_ channel activation in vascular cells as a feedback response that is activated when the delivered blood cannot fully compensate for reduced nutrient levels. Recognizing the involvement of these feedback responses, along with other possible paths [23,25], our study aimed to develop a model of the feedforward NGVC responses that shape arteriolar vasodynamics in a dynamically functioning mouse brain.

Arteriolar vasodynamics is largely driven by neuronal activity, with the reported correlation between the two estimated at approximately 60% in awake mice [26]. Also, astrocytes have been shown to contribute to CBF regulation [27,28]. An open question is whether astrocytes dynamically modulate arteriolar vasodynamics and reduce this direct correlation. A recent study demonstrated that inhibiting astrocyte endfoot calcium signaling suppresses the delayed and secondary phases of sensory-evoked vasodilation in mouse penetrating arterioles (PAs) during sustained FH [12]. This finding suggests that astrocytes may regulate arteriolar vasodynamics conditionally, either by (1) inducing vasodilation in response to metabolic factors and/or (2) periodically initiating vasodilation at specific times. To investigate this conditional involvement, we employed a systems biology approach to disentangle neuronally from astrocyte-evoked vasodynamics and determine whether astrocyte-driven vasodynamics accounts for the portion of arteriolar vasodynamics uncorrelated to neuronal activity that observed in the awake mouse brain.

Vasodynamics is observed even in the absence of neuronal activity and is characterized by spontaneous low-frequency oscillations known as vasomotion. Vasomotion is considered a mechanism that facilitates efficient blood delivery and waste clearance [29–32]. Vasomotion is also observed in other organs, such as the skin [33], mesentery [34], and crucially, in isolated arterioles lacking systemic or neurogenic input [35]. This latter observation supports a vascular-intrinsic origin for vasomotion [36], distinct from other rhythmic fluctuations in cerebral hemodynamics driven by Mayer waves [37], neurovascular coupling [38], or cardiac and respiratory activities [39]. While the underlying mechanisms of vasomotion are not well understood, most previously proposed models have relied on cytosolic positive feedback through Ca^2+^ release channels (ryanodine or IP3 receptors) for sustaining oscillations [40,41]. A common strategy for generating oscillations in biological regulatory systems involves negative feedback with time delay [42]. Here, we hypothesized that vasomotion may emerge from the temporal dynamics of myogenic autoregulation, where passive distension amplifies delayed myogenic responses (negative feedback), leading to self-sustained oscillations in the cerebral vasculature. Using hemodynamic simulations in a simplified model of a network of coupled PAs, we investigate whether, in the absence of a cytosolic positive feedback loop, the model can still generate network-wide oscillations that resemble in-vivo vasomotion.

In our earlier work, we developed a coarsely segmented model of mouse cerebral vasculature, featuring a closed circulatory system and key morphological characteristics of mouse cerebrovasculature for in-silico analysis of static autoregulation [43]. In this study, we extend that framework to simulate hemodynamic and vasodynamic interactions within the vessel network under both active and resting brain states. This approach addresses a gap in existing computational NGVC studies by incorporating the often-overlooked influence of hemodynamics on vasodynamics. First, we build a cellular-level model of mouse PAs using a mathematical representation of their mechanobiological and electrophysiological properties and incorporate signaling pathways from other cell types that dynamically affect these properties (Section 1). We then use this PA model to simulate the kinetics of our proposed vasomotion (Section 2) and NGVC models (Section 3).

## Results

### Section 1: A cellular-level model of mouse penetrating arteriole

In this study, we focused on PA vasodynamics for two reasons: 1) PAs are known to be the bottleneck of blood perfusion to the cortex [43–45], and 2) vasodynamic recordings of PAs are available in many studies, including those where NGVC was manipulated to silence a specific signaling pathway either genetically [9] or pharmacologically [12]. The myogenic response, regulated by hemodynamics and influenced by NGVC, is the primary driver of PA vasodynamics. Regulation of the myogenic response in PAs by hemodynamics is primarily adjusted by arteriolar SMCs (aSMCs) and, to a lesser extent, by arteriolar endothelial cells (aECs) [46]. In this section, we first design an endothelium-denuded segmented model of a mouse PA that incorporates an effective myogenic response by using a cellular-based model of aSMCs. Next, we add a simplified model of aECs to each PA segment and integrate this segmented vessel model into different PA segments within a larger cerebrovascular model.

**Fig 1:**
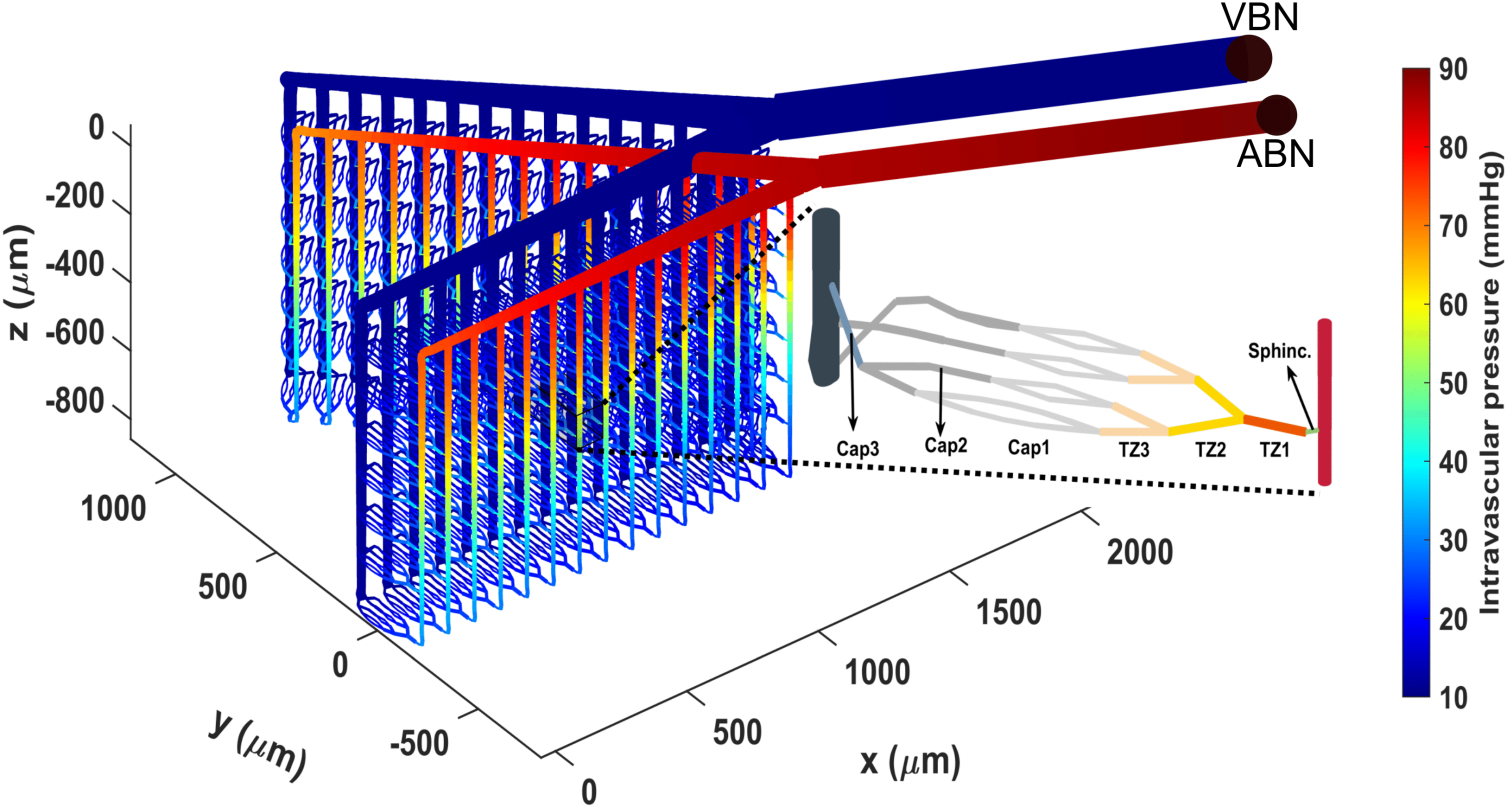
Designed segmented cerebral microvasculature model which includes two boundary nodes (ABN: artery boundary node, VBN: vein boundary node), a pial arteries network, and 30 PAs. The zoomed-in area highlights the structure of microvessels, including a sphincter, three layers of TZ segments (up to the 3rd order), and three layers of capillaries (beyond the 3rd order). The color coding illustrates the pressure distribution across the vascular segments, with boundary node pressures, ABNP and VBNP, set at 90 and 10 mmHg, respectively.

The cerebrovascular model used in this study is an extension of our earlier work, where we designed an artificial cerebrovascular model in the form of a graph-based network composed of interconnected segmented cylindrical tubes with specific length and diameter [43] (Fig 1), determining the resistance to blood flow according to Poiseuille’s law [47]. Briefly, we assumed that a typical PA in a mouse brain extends 840 μm into the tissue, bifurcating at various depths to supply capillaries, with deeper segments accommodating less blood flow and having smaller diameters than superficial ones, as informed by Murray’s minimum-cost hypothesis [48]. Accordingly, the diameter of a PA, consisting of 28 segments, each 30 μm long, was linearly reduced from 18 μm at the surface to 12.6 μm in the deepest cortical layers. In the following sections, we first model a vessel segment at 250 μm depth with a maximum active diameter of 16.4 μm and then extend this vessel segment model to all PA segments to create a PA model.

#### Endothelial-denuded segmented model of mouse penetrating arterioles.

The diameter of a vessel segment is the result of the balance between two opposing processes: passive distension and active constriction. The magnitude of passive distension depends on the vessel’s distensibility and is modulated by circumferential wall stress (WS) [49]. The magnitude of active constriction in each vessel segment depends on the density of surrounding mural cells and is regulated by specific biomechanical forces exerted by the blood on the vessel wall. The exact biomechanical forces and mechanisms through which vascular cells (mural cells and endothelial cells) sense these forces and subsequently modulate their ionic channels are not fully understood, but are thought to mitigate the destructive forces of increased WS and wall shear stress (WSS).

In our earlier study, within the hypothetical autoregulation range for the designed cerebrovascular model, where the artery boundary node pressure (ABNP) varies between 40 and 130 mmHg while the vein boundary node pressure (VBNP) is held constant at 10 mmHg (Fig 1), the constriction force of mural cells is linearly potentiated by the vessel WS [43,50]. According to Laplace’s law, this stress is directly proportional to the intravascular pressure (IP) and the internal diameter (*D*) of the vessel segment, and inversely proportional to the segment’s wall thickness (Δ), expressed as $WS=\frac{IP\cdot D}{\Delta }$. We did not account for the cytoskeletal remodeling, which likely occurs in pathological conditions like hypertension [49], and assumed that wall thickness remains constant under varying ABNP values. We quantified WS in all PA segments at ABNP = 130 mmHg (*WS*_max_) and used these values to model a linear relationship between SMC ion channel modulation and WS within the autoregulation range.

Myogenic tone (MT) development is primarily governed by SMC depolarization, activation of voltage-operated calcium channels (VOCCs), and an increase in intracellular calcium concentration [49]. Cell depolarization occurs either through the potentiation of depolarizing currents or the reduction of hyperpolarizing currents. Multiple ion channels have been implicated in the depolarization of SMCs (for review, see references [49] and [23]). Informed by the literature, our proposed SMC model includes WS-activated ion channels, such as TRPC6 [51], TRPM4 [52], and Ca^2+^-activated Cl^−^ channels (TMEM16A) [53,54], which play key roles in potentiating depolarizing currents in response to increase in WS, and WS-deactivated ion channels, including Kir channels [55,56], which inhibit hyperpolarizing currents as WS increases.

**Fig 2:**
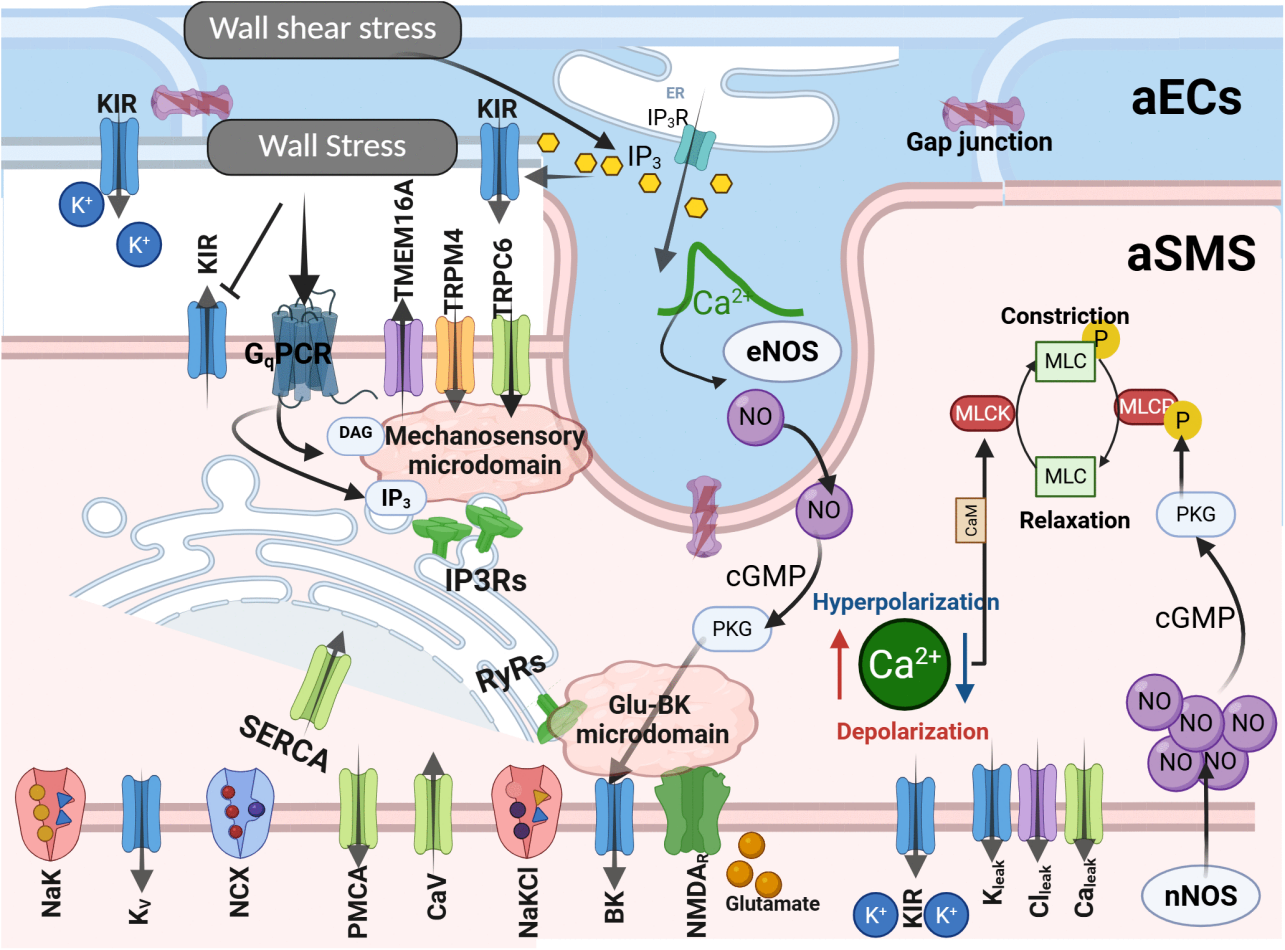
A schematic of the proposed SMC-EC model. Created in BioRender. For a detailed description we refer readers to the Methods section. The figure illustrates the channels and pumps regulating intracellular ion concentration and membrane potential in aSMCs, with color coding: potassium channels (blue), calcium channels and pumps (green), chloride channels (purple), and sodium channels (orange). The model includes two microdomains where calcium concentration can exceed the cytoplasm calcium concentration in aSMCs. The first is the mechanosensory microdomain, containing transient receptor potential (TRP) channels (e.g., TRPC6, TRPM4) and calcium-activated chloride channels (TMEM16A), which produce depolarizing currents in response to WS. In this microdomain, SMC mechanosensors are assumed to linearly convert WS into downstream signaling molecules, such as IP3 and DAG, modulating depolarizing currents. The second microdomain is the Glu-BK microdomain, where NMDA receptors, activated by neuron-released glutamate, trigger calcium efflux that activates nearby BK channels. The open probability of aSMC Kir channels is reduced by increasing WS, while larger WSS raises IP3 concentration within aECs, increasing both aEC Kir channel open probability and eNOS activity. The steady-state open probability of aSMC BK channels is increased by eNOS-dependent increases in NO/cGMP. In aSMC constriction mechanics, the rapid modulation of MLCK and MLCP activity is primarily driven by changes in cytoplasm SMC Ca^2+^ concentration and nNOS-dependent increases in NO/cGMP during increased neural activity, respectively.

**Fig 3:**
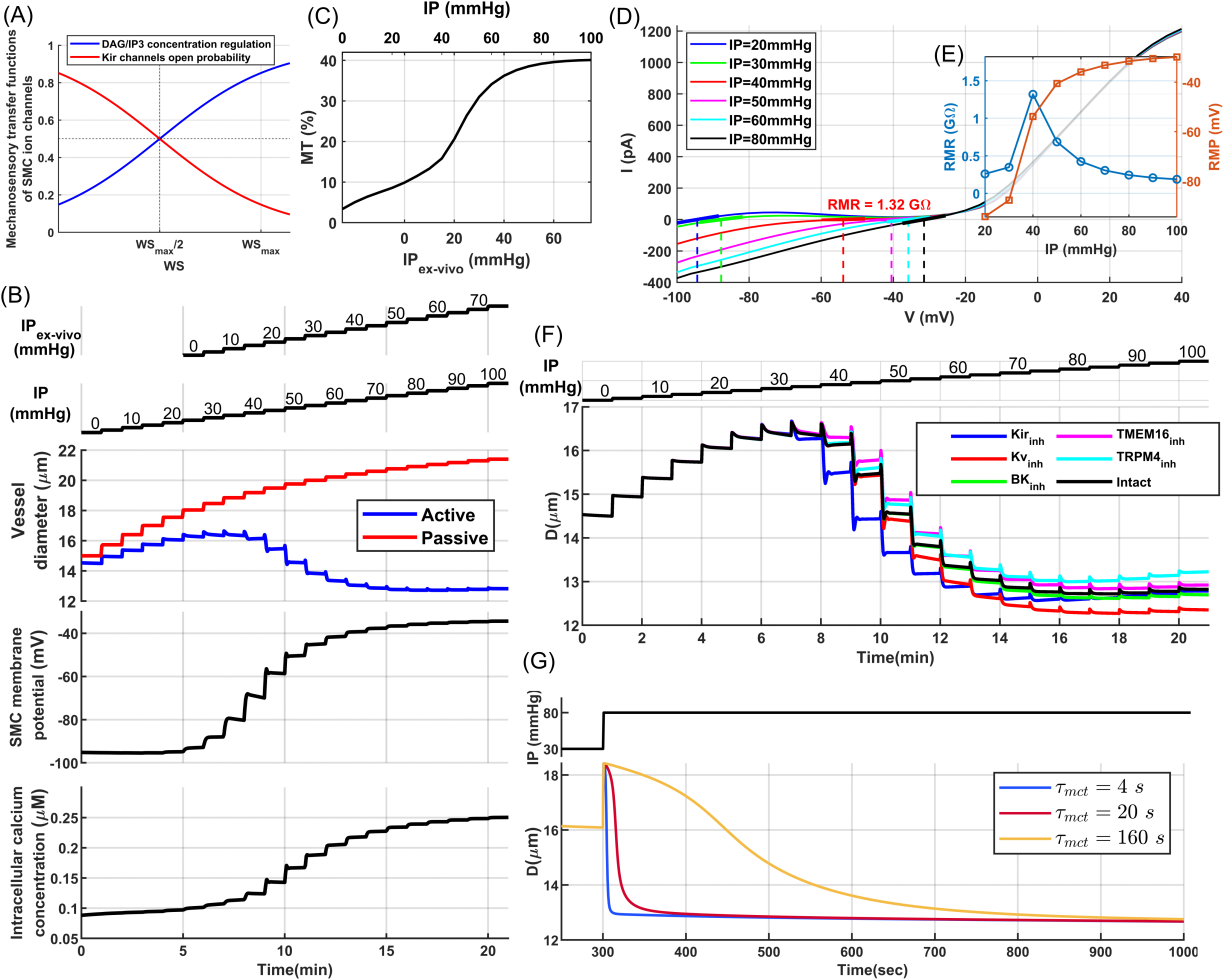
Endothelial-denuded segmented model of mouse PAs: (A) Quasi-linear sigmoidal function model the relationship between WS and mechanotransduction signaling molecules (IP3, DAG) and the WS-Kir_OP_ signaling pathway. **(B)** Pressure myography maneuver conducted on an endothelium-denuded vessel segment to calculate MT. **(C)** MT calculated for this segment, based on the curves plotted in panel (d-top), using $\left(\frac{D_{\text{passive}}-D_{\text{active}}}{D_{\text{passive}}}\right)\times 100$. **(D)** The aSMC I-V curve under various IP values from simulated voltage-clamp analyses. Vertical lines depict the RMP where membrane current is near zero. The reciprocal of the slope of the tangent lines at the intersections of the vertical lines and curves indicates RMR. **(E)** Calculated RMR and RMP at different IP values. **(F)** Pressure myography test simulated under the assumption of 50%-inhibition of the main ion channels involved in MT regulation. **(G)** Simulated vasodynamics of the segmented model in response to a sudden increase in IP from 30 to 80 mmHg, analyzed under various values of the mechanotransduction time constant ($\tau_{mct}$).

Fig 2 presents a schematic of the proposed aSMC-aEC model. Briefly, we assumed a linear relationship between the concentration of certain mechanotransduction signaling molecules (inositol trisphosphate (IP3) and diacylglycerol (DAG) [51]), and the exerted WS that potentiates depolarizing channels. This assumption is supported by several studies showing that increasing IP stimulates the phospholipase C (PLC) activity, which subsequently increases the concentration of second messengers (IP3 and DAG) in SMCs [57,58], with subsequent upregulation of ion channels (TRPC6, TRPM4, and TMEM16A) to potentiate depolarizing currents. Fig 3A depicts the relationship between the concentration of these second messengers and WS in our model, and for Kir channels, we simply assumed that their open probability (Kir_OP_) is directly downregulated by WS.

We evaluated the performance of our proposed endothelial-denuded segmented PA model through simulated pressure myography and patch-clamp tests before integrating it into the cerebrovascular model. Physiologically, external mechanical factors such as extravascular pressure, bath osmolarity [55], and the structural constraints of surrounding tissue impose additional stress on SMCs that scales with intravascular pressure [59]. In our in-silico framework, we omitted these factors to avoid introducing additional model complexity. To facilitate comparison of model outputs with ex-vivo data, we introduced an adjusted intravascular pressure (IP_ex-vivo_), defined as a constant 25 mmHg offset of the in-silico IP, to approximate the net mechanical influence of these factors on the force sensed by SMCs.

Initially, we simulated a pressure myography test to evaluate MT development in our model through stepwise increases in IP within a PA segment under calcium-free (passive) and nominal external calcium concentration (active) conditions (Fig 3B). Model parameters for passive distension were set so that the maximum passive diameter at high IP values reached about 130% of the maximum active diameter of the vessel segment [46]. For the active response, as expected, pressure elevation prompted membrane potential depolarization, leading to increased intracellular calcium concentration and subsequent vessel constriction (Fig 3B).

Under low IP conditions (0–25 mmHg), corresponding to unpressurized vessels ex-vivo, WS-dependent depolarizing channels are less active, and a high open probability of Kir channels maintains the membrane potential near the equilibrium potential of potassium. Under these conditions, intracellular [Ca^2+^] is low, aSMCs are less capable of constricting the vessel, and the vessel passively distends until depolarizing channels become more active with increased WS, while simultaneously the open probability of Kir channels decreases. This leads to cell depolarization and a subsequent increase in [Ca^2+^] to a level that induces active constriction. The maximum active diameter of this vessel segment is achieved within the IP range of 30–35 mmHg. This IP range is in agreement with our cerebrovascular model design, where a PA segment at the depth of 250 μm reaches its maximum dilation at the IP value between 30–35 mmHg when ABNP is set to 40 mmHg, which is the lower limit of the autoregulation range. Consistent with the in-silico and in-vivo analysis of autoregulation [43,60], vessels must initially constrict steeply after reaching their maximum dilation to be at the optimal point of autoregulation, and as pressure increases, even a small constriction generates significant resistance to blood flow, optimizing the IP distribution in the network at the upper limit of the autoregulation range (Fig 3D). The simulated values of membrane potential and [Ca^2+^] at various IP_ex-vivo_ levels (Fig 3B) are aligned with ex-vivo measurements for PAs [61–63], and the calculated MT of the modeled vessel segment (Fig 3C) is consistent with the experimental data for PAs in both the maximum tone and profile [46,64], indicating that the active constriction and passive distension models are effectively simulating the myogenic response of a PA vessel segment.

Next, we examined the resting membrane potential (RMP) and resting membrane resistance (RMR) which are critical electrophysiological parameters of mural cells. We simulated several voltage clamp tests under various IP levels and we measured the steady-state membrane current while changing the SMC membrane potential from -100–40 mV (Fig 3D). The generated I–V curves at IP = 20 and 30 mmHg are in good agreement with electrophysiological profiles of freshly isolated SMCs from mouse brain arterioles [55,56,65]. At these low IP values, the high open probability of Kir channels and reduced dominance of WS-depolarizing channels result in an I–V curve for the SMC model that shows the typical characteristics of Kir channels [66,67] including the negative slope and inward rectification. In isolated SMCs, reducing extracellular osmolarity and the consequent cell swelling suppresses Kir channel activity [55], an effect comparable to the shift observed in our simulations when IP increased from 20 to 30 mmHg under 4 mM [*K*^+^]_ex_. As IP increases across the physiological range, the I–V curve of the cell has a shallow slope (reflecting low ion permeability) and maintains a high RMR, which is a characteristic of excitable cells. RMR reaches its maximum value at the lower limit of the physiological range, and as IP increases, RMR decreases and RMP rises (Fig 3D and 3E), indicating that basal IP can modulate both RMP and RMR in SMCs, thereby affecting the sensitivity of the SMC membrane potential to external stimuli such as changes in hemodynamics or membrane potential of aECs.

In vascular SMCs, specific potassium channels operate under physiological conditions, mediating potassium efflux to counteract WS-dependent depolarizing currents, providing negative feedback for MT regulation [67], and maintaining a large RMR in aSMCs to enable rapid responsiveness to external stimuli. Arteriolar SMCs predominantly express four types of potassium channels [67]: ATP-sensitive (K_*ATP*_), large conductance Ca^2+^-activated (BK), inward rectifier (Kir), and voltage-gated (KV). It was suggested that K_*ATP*_ channels play a minor role in cortical arterioles [25]. Furthermore, pressure-induced depolarization in arteriole SMCs triggers an amplification of Ca^2+^ sparks due to the facilitated Ca^2+^-induced Ca^2+^ release (CICR) process in ryanodine receptors (RyRs), driven by increased Ca^2+^ efflux via adjacent T-type VOCC [68], forming a negative feedback loop with coupled BK channels that mitigates myogenic vasoconstriction by promoting SMC hyperpolarization. However, unlike cortical surface arteries, BK channel-mediated K^+^ efflux does not inhibit MT significantly in PAs, and under physiological conditions, only Kir and KV channels are highly activated in aSMCs[67]. Consequently, in our proposed SMC model, TMEM16A and TRPM4 channels are key depolarizing channels, while Kir, KV, and BK channels serve as key hyperpolarizing channels, with a less contribution from the latter.

To evaluate the contribution of these channels to SMC constriction, we simulated a pressure myography test under the assumption of 50% channel inhibition (Fig 3F). Kir channel inhibition significantly disrupted MT regulation within the physiological IP range. These simulation results align well with in vitro findings, where vessels exposed to a Kir channel blocker under physiological IP were significantly constricted [55], suggesting that SMC Kir channels play a crucial role in cerebral autoregulation. KV and BK channel inhibition caused MT dysregulation at mid to high IP values due to their voltage-dependent activation mechanism, with BK channels playing a much lesser role in the negative feedback regulation of MT in endothelium-denuded PAs. Inhibition of both key depolarizing channels, TRPM4 and TMEM16A, led to reduced vessel constriction.

Although the steady-state SMC mechanotransduction signaling pathways (such as DAG, IP3, and Kir_OP_) are regulated by WS, the cerebral vasculature operates in a dynamic environment where WS continuously fluctuates across vessel segments due to events like FH or changes in mean arteriolar pressure. Because mechanotransduction in SMCs does not occur instantaneously, there is a time delay in the adjustment of WS-dependent ion channel activity in response to rapid changes in WS. We modeled this delay using an exponential function with a mechanotransduction time constant ($\tau_{\text{mct}}$). This time constant plays a crucial role in shaping the temporal dynamics of the myogenic response, influencing the kinetics of FH and vasomotion, as explored in the following sections.

Various studies affirm that Neuropeptide Y (NPY) can enhance the vasoconstrictor actions of other molecules [69–72]. This facilitating effect is attributed to the synergistic interactions between NPY receptors (NPYRs) and other G-protein-coupled receptors (GPCRs) involved in vasoconstriction [73]. This synergy could potentiate transduction pathways following the mechanoactivation of Gq-coupled receptors in SMCs and lower the $\tau_{\text{mct}}$. We estimate that under physiological conditions, the value of $\tau_{\text{mct}}$ could range between 1–20 seconds and will provide evidence to support this. However, in-vitro conditions may result in much larger $\tau_{\text{mct}}$ values and a myogenic response that is several orders of magnitude slower than under physiological conditions [46,49,64,74]. Facilitation of the myogenic response by NPY is particularly relevant in cerebral arterioles, as NPY receptors are highly expressed in aSMCs, and there are direct NPY interneuron–aSMC junctions [75]. Fig 3G shows vessel diameter changes in response to an abrupt increase in IP from 30 to 80 mmHg in our model under three different $\tau_{\text{mct}}$, where the vessel initially passively distends, and the time to reach steady state can vary significantly depending on the value of $\tau_{\text{mct}}$.

#### Fast-acting downregulation of SMC constriction in endothelial-denuded vessel segment.

To simulate arteriolar vasodynamics, we need to identify and model the fast-acting downregulators of SMC constriction that induce rapid vasodilation during periods of increased neural activity. The regulation of SMC constriction involves two competing processes: myosin light chain (MLC) phosphorylation by myosin light-chain kinase (MLCK), counteracted by dephosphorylation through myosin light-chain phosphatase (MLCP). A decrease in intracellular calcium concentration inhibits calcium-calmodulin-dependent MLCK activation, thereby promoting muscle relaxation [76]. Additionally, MLCP is activated by protein kinase G (PKG) and MLCK is inhibited by protein kinase A (PKA), with PKG and PKA themselves being activated by secondary messengers cyclic guanosine monophosphate (cGMP) and cyclic adenosine monophosphate (cAMP), respectively. These secondary messengers are primarily produced by NO-dependent sGC [77] and hormone/neurotransmitter-stimulated adenylyl cyclase (AC) [78,79], respectively.

SMC intracellular calcium concentration is predominantly regulated by VOCCs, and reducing the SMC membrane potential or directly inhibiting VOCCs are the primary mechanisms of lowering SMC [Ca^2+^] and MT inhibition. Based on the reported in-vivo SMC [Ca^2+^] [80], we ruled out the possibility that fast VOCC inhibition via NGVC affects SMC constriction state during FH. We only focused on modeling the mediators that can rapidly modulate SMC membrane potential. Modulation of the SMC membrane potential is a multi-faceted process which is affected by hemodynamic changes, synaptic-like transmission between neural axons and SMCs [75], myoendothelial gap junctions [10], NO pathways [81], acidosis [25], and direct extracellular interactions between ion channels, such as astrocyte endfeet BK channels and SMC Kir channels [82]. We ruled out the possibility of the brain tissue acidification during FH, but other pathways were modeled in this study.

**Fig 4:**
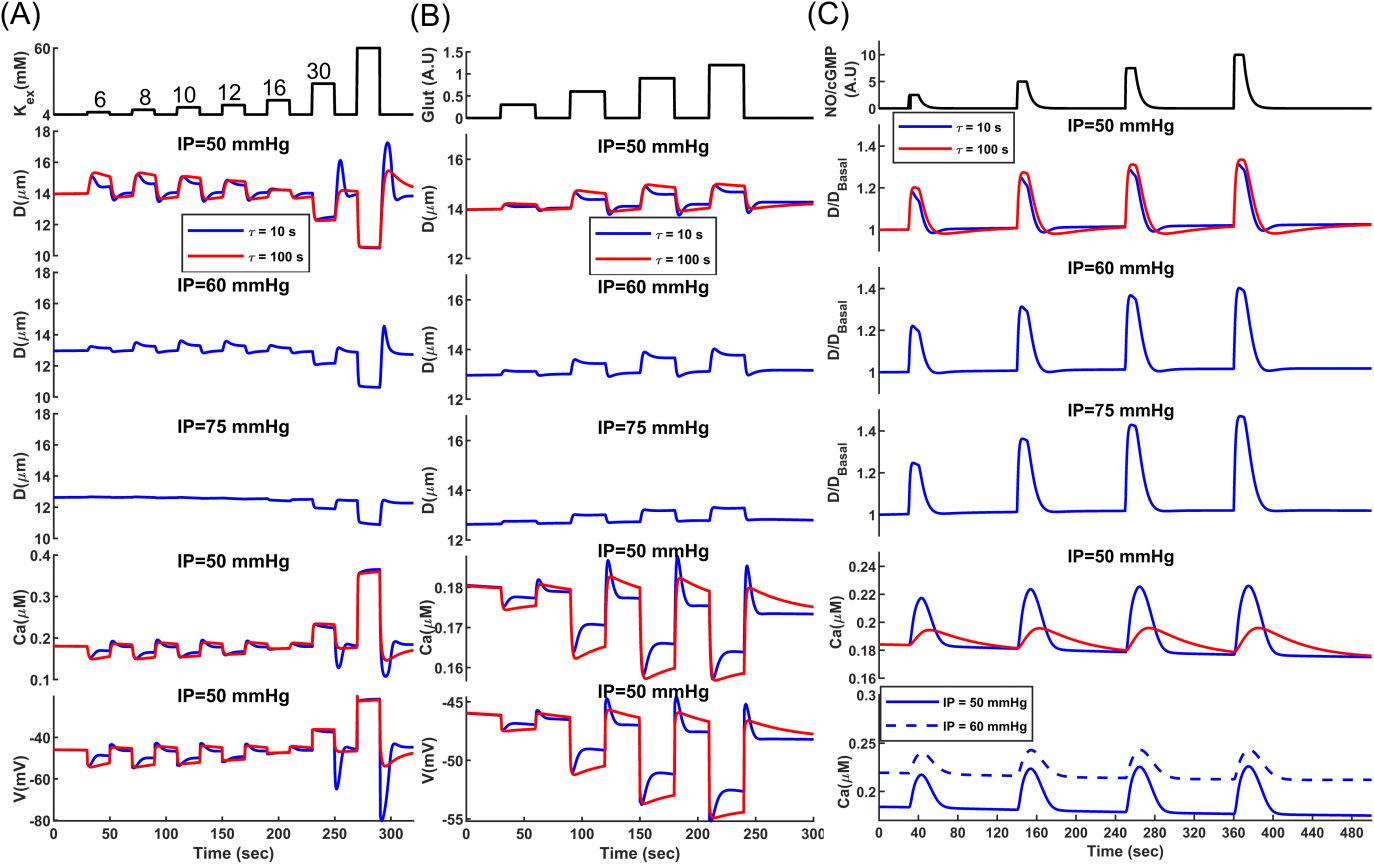
Analysis of vasodynamics in an endothelium-denuded model under the influence of vasodilatory mediators. Vasodilatory responses were measured for all mediators under two $\tau_{\text{mct}}$ and three IP values. **(A)** Changes in vasodynamics, SMC membrane potential, and [Ca^2+^] when increasing the SMC extracellular potassium concentration from a baseline of 4 mM up to 60 mM in discrete steps. The transient hyperpolarization overshoot observed after washout of extracellular K^+^ (60 to 4 mM) is primarily Kir channel-dependent. The abrupt negative shift in the K^+^ reversal potential (*E*_*K*_) transiently relieves rectification block, enhancing Kir channel conductance and producing a brief surge of outward current and hyperpolarization. In our simulations, elimination of Kir channel conductance abolished this overshoot, confirming its Kir channel origin. The ensuing dilation increases wall stress, after which WS-activated depolarizing currents and suppression of Kir channel activity restore *V*_*m*_ toward baseline. **(B)** Changes in vasodynamics, SMC membrane potential, and [Ca^2+^] when increasing the glutamate concentration from a baseline of 0 to 1.2 AU (arbitrary unit) in four discrete steps. **(C)** Changes in vasodynamics and [Ca^2+^] when increasing SMC NO/cGMP concentrations from a baseline of 0 to 10 AU in four discrete steps. Numbers considered for the concentration of glutamate and NO were hypothetical since the exact physiological values are unknown. Model parameters were adjusted accordingly to produce the maximum dilatory response at the highest hypothetical concentrations.

Kir channels are key candidates for modulating SMC membrane potential via NGVC, as they play a crucial role in SMC membrane potential autoregulation (Fig 3F). The vasodilatory response to the potassium released from astrocyte endfeet BK channels is mediated by SMC Kir channels [82–84]. Elevation of extracellular potassium increases Kir channel conductance which hyperpolarizes the cell. Fig 4A shows the vasodilatory response of the PA segmented model to the elevation of extracellular potassium ([*K*^+^]_ex_) at discrete levels for three IP and two SMC $\tau_{\text{mct}}$ values. At IP = 50 mmHg, extracellular potassium elevation induces the largest vasodilation compared to higher IPs. In this physiological IP range, the cell’s RMR is large (Fig 3D and 3E), and slight variations in Kir channel conductance can change the SMC membrane potential significantly. At higher IP values, RMR and Kir channel open probability decrease, making Kir channels less capable of inducing hyperpolarization in the SMC.

The SMC mechanotransduction time constant ($\tau_{\text{mct}}$) can also affect the magnitude of vasodilation. When $\tau_{\text{mct}}$ is small, SMCs respond rapidly to WS changes. The initial vasodilation increases WS according to Laplace’s law, leading to cell depolarization that counteracts Kir channel-induced hyperpolarization. This myogenic response is more pronounced at lower IP values because, under these conditions, the cell’s RMR is large, and slight changes in WS-dependent currents have a large impact on the SMC membrane potential.

At IP = 50 mmHg, the largest vasodilation was observed at [*K*^+^]_ex_ = 8 mM, but the peak shifts to higher potassium concentrations at IP = 60 mmHg because altering extracellular potassium not only impacts Kir channel conductance but also changes the potassium Nernst potential. Increasing IP also raises intracellular potassium concentration, further affecting the potassium Nernst potential [85]. Elevating extracellular potassium up to 16 mM induces vasodilation at IP = 50 and 60 mmHg, but it results in slight constriction at IP = 75 mmHg. Therefore, the direction and extent of diameter change depend on both extracellular potassium concentration and the value of IP [82]. At a high [*K*^+^]_ex_, the potassium Nernst potential is much larger than its physiological level, leading to a significant SMC depolarization (∼-20 mV at [*K*^+^]_ex_ 60 mM) and causing maximum constriction in the vessel, which typically does not occur at any IP value under physiological extracellular potassium concentration.

BK channels are modulated by the membrane potential and Ca^2+^ sparks near the cell membrane, which makes them key candidates for fast hyperpolarization and relaxation of aSMCs [86]. Studies have shown that BK channel activation can induce pronounced vasodilation in PAs when exogenous activators are present. For example, brain tissue acidification dilated PAs rapidly due to a dramatic increase in Ca^2+^ spark activity and BK channel function, driven by the enhanced RyR open probability [25]. BK channels in PA SMCs are also colocalized with GluN1, a subunit of NMDA receptors, and glutamatergic neurons have been shown to dilate arterioles through synaptic-like transmission at neural–aSMC junctions (NsMJs). Activation of NMDA receptors in Glu-NsMJs leads to a Ca^2+^ ion influx, which binds to and activates BK channels, resulting in increased potassium ion efflux, membrane hyperpolarization, and subsequent relaxation of aSMCs. In-vivo disruption of Glu-NsMJs inhibits NGVC substantially [75]. To incorporate this NGVC mechanism, we included a Glu-BK microdomain in our model, where activation of glutamate receptors locally increases [Ca^2+^] in the microdomain and activates BK channels (Fig 2).

Fig 4B shows the vasodilatory response of the PA segmented model to elevated glutamate at four discrete hypothetical concentrations, across three IP and two SMC $\tau_{\text{mct}}$ values. The highest hypothetical glutamate concentration induces a 3.5% steady-state dilation at IP = 50 mmHg (after balancing neurogenic and myogenic responses when $\tau_{\text{mct}}$= 10 s), while this level is about 5% at IP = 60 mmHg. This occurs because BK channels are mediated by both Ca^2+^ and membrane potential. At IP = 60 mmHg, the higher SMC membrane potential enhances the vasodilatory effect of glutamate. The myogenic response following initial vasodilation is less pronounced at IP = 75 mmHg due to small RMR, where changes in WS-dependent currents are less capable of altering the membrane potential and the [Ca^2+^]. The post-stimulus undershoot in the diameter change (observed in the blue curves of Fig 4A and 4B) has the same origin as the initial overshoot and is associated with the potentiation/inhibition of myogenic response following vasodilation/constriction. Once glutamate or [*K*^+^]_ex_ return to the baseline, the membrane potential is expected to return to its baseline. However, since MT is larger than its basal level in the dilation phase, following the vasoconstriction due to the removal of the stimulus, the delay in the adjustment of MT causes the vessel to further constrict, which produces a post-stimulus undershoot.

Nitric oxide (NO) is a potent vasodilator produced rapidly by both endothelial (eNOS) and neural nitric oxide synthase (nNOS) and plays a crucial role in NGVC [87]. In-vivo evidence shows that neuronally produced NO acts directly on arterioles [9,88,89]. Blockade of neural NO synthase has the most significant inhibitory effect on the feedforward mechanisms [87], which can be compensated by feedback mechanisms [90]. NO relaxes vascular SMCs and dilates blood vessels by increasing the intracellular cGMP concentration, which stimulates cGMP-dependent PKG activity. However, the rapid vasodilatory signalling pathway mediated by PKG is not fully understood. This complexity arises from the diverse ways that PKG can influence the SMC constriction state. PKG modulates various SMC ion channels, including the activation of BK channels [91], inhibition of TRPM4 channels [81], and inhibition of VOCCs [92], collectively leading to decreased SMC [Ca^2+^]. PKG also directly activates MLCP [93,94]; however, it has remained unclear whether PKG-dependent MLCP activation or PKG-SMC ion channel modulation plays a more significant role during the rapid dynamics of FH. Here, as one possible mechanistic scenario, we assume that the fast-acting effect of NO on aSMCs is primarily governed by the increased MLCP activity rather than by SMC ion channel modulation. This assumption is supported by ex-vivo studies showing that the early phase of NO-induced vasodilation involves the suppression of CPI-17 phosphorylation which is an MLCP inhibitory protein [95–97], and will be further evaluated against in-vivo experimental evidence in the following sections. Therefore, in our vessel wall mechanical model adopted from [98–100], variations in NO/cGMP/PKG activity directly affect the kinetics of actin-myosin interactions, ultimately leading to SMC relaxation.

Fig 4C shows the vasodilatory response of the PA segmented model to elevated NO/cGMP at four discrete hypothetical levels, across three IP and two SMC $\tau_{\text{mct}}$ values. Despite the immediate rise in NO/cGMP concentration, we assumed a degradation rate for these molecules that delays their decline under physiological conditions. This degradation was modeled by a decaying exponential function with a 5-sec time constant, regardless of any dose dependency that might exist in reality. The relative diameter change of NO-induced dilation is larger at a higher basal IP. This occurs because the increase in MLCP activity correlates with a decrease in MLC phosphorylation sensitivity to [Ca^2+^] [95], resulting in [Ca^2+^] having a less pronounced effect on SMC contraction during periods of elevated NO/cGMP. Therefore, during NO/cGMP dose-dependent relaxation of SMCs, vessels with higher basal IP experience larger passive distention, leading to a more pronounced diameter change.

If the myogenic response is defined as the intrinsic ability of SMCs to modulate their membrane potential and [Ca^2+^] by WS, then increased MLCP activity due to the NO/cGMP/PKG pathway can be seen as a mechanism of dampening this response by reducing the sensitivity of MLC phosphorylation to MLCK activity. On the other hand, hyperpolarizing SMCs with mediators can be viewed as the inhibition of myogenic response. During NO-induced dilation, the increase in diameter and WS leads to cell depolarization, causing a rise in [Ca^2+^] and potentiation of the myogenic response (Fig 4C). However, since the NO/cGMP/PKG pathway dampens the myogenic response, the effect of the potentiated myogenic response is small and negligibly counteracts the vasodilatory effect of NO. Comparing the NO vasodilatory response under two analyzed mechanotransduction time constants at IP = 50 mmHg shows that a smaller $\tau_{\text{mct}}$ leads to a sooner and more pronounced increase in [Ca^2+^], but this potentiation of the myogenic response during the dilatory phase only negligibly reduces the peak amplitude of NO-induced dilation compared to the case of a larger $\tau_{\text{mct}}$.

Rapid myogenic response potentiation ($\tau_{\text{mct}}$ =10 s) can help the vessel return to its basal level sooner after the stimulus; however, the slow degradation rate of NO/cGMP and the dampened myogenic response in the presence of these molecules does not lead to a pronounced post-stimulus undershoot. As NO/cGMP degrades and returns to its basal state, the myogenic response may cause a post-stimulus undershoot if [Ca^2+^] is above pre-stimulus levels. In the $\tau_{\text{mct}}$ =100 s case, where the myogenic response occurs more slowly, once NO/cGMP returns to its basal state and Ca^2+^ sensitivity of MLC phosphorylation increases, the post-stimulus undershoot period is longer. However, due to a smaller increase in myogenic response-induced [Ca^2+^], the degree of post-stimulus undershoot is only marginally larger than in the fast myogenic response scenario, where a significant rise in [Ca^2+^] occurs alongside the reduced Ca^2+^ sensitivity of MLC phosphorylation. Similar to the vasodilatory response of other mediators, like [*K*^+^]_ex_ and glutamate, the degree of post-stimulus undershoot decreases with increasing IP. This occurs because, under higher IP, cell RMR is small, and WS-dependent depolarizing currents have a reduced impact on the membrane potential, resulting in a smaller increase in [Ca^2+^] (Fig 4C, bottom panel). Consequently, smaller post-stimulus undershoots are observed at IP = 75 mmHg and IP = 60 mmHg compared to IP = 50 mmHg. In the following sections, as we analyze the neurogenic impulse response of the CBF regulatory system, we will present strong experimental evidence to support our analysis of the dynamics of NO-induced vasodilation.

#### Integrating segmented model of mouse penetrating arterioles into cerebral vasculature model.

Thus far, we have developed a segmented model of an endothelium-denuded vessel and simulated the primary vasodilatory signaling pathways. Endothelial cells are also well recognized for their crucial role in FH dynamics. In this section, we propose a simplified model of aECs to be added to the PA segment model, which we then integrate into the PA segments of the cerebral vasculature to capture their physiological contribution to FH dynamics. ECs are known to serve as sensors of neural activity within the CBF regulatory system, relaying this information retrogradely up the vascular tree via electrical coupling [10]. Active neurons discharge potassium ions, which enhance the conductance of Kir channels in ECs, leading to fast hyperpolarization of arteriolar ECs in both proximal and distal regions of the activated area, which is transmitted to SMCs through MGJs and results in their dilation [101,102].

ECs sense changes in potassium concentration and translate that to changes in their membrane potential. A large RMR, in the range of several Gigaohms [103,104], makes ECs sensitive to such changes, allowing them to respond quickly and precisely. [66] proposed an abstract mathematical model for ECs, which includes a model for the Kir current (*I*_*Kir*_) while combining all other transmembrane currents into one nonspecific linear background current, *I*_*bg*_(*v*) = *G*_*bg*_(*V*_*EC*_ − *E*_*bg*_), where *E*_*bg*_ = −30 mV was assumed to be constant. In our proposed model, we replaced *I*_*bg*_ with $I_{MGJ}=\frac{1}{R_{MGJ}}(V_{EC}-V_{SMC})$. Here, *R*_*MGJ*_ represents the resistance of gap junctions between an EC and SMC which was assumed to be 1 GΩ [66,105]. This replaced depolarizing current opposes *I*_*Kir*_-induced hyperpolarization to maintain a large RMR in ECs, and couple the EC membrane potential with the membrane potential of mural cells for autoregulation.

**Fig 5:**
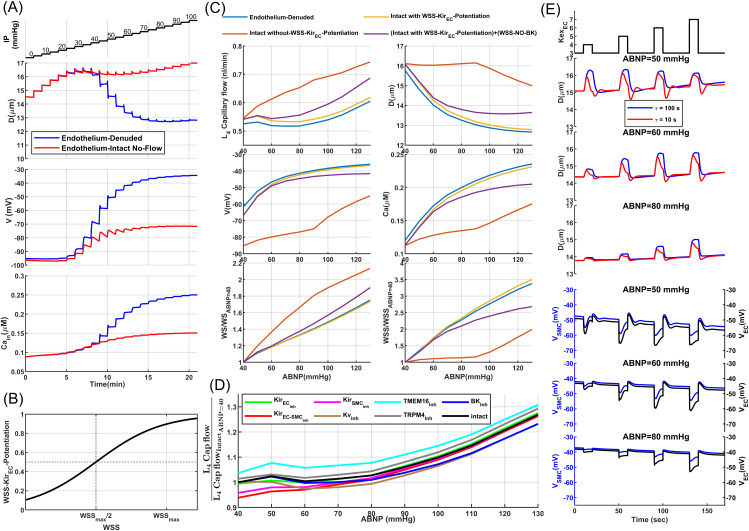
Integration of Intact segmented model of mouse PAs into the cerebral vasculature model: (A) pressure myography test performed on an intact vessel segment with no blood flow and exerted WSS. Note: these simulation results are not physiologically valid, as no WSS dependency for EC-Kir channel activation was considered in the model. In the absence of flow, EC-Kir channels are deactivated (panels B, **C)**, and MT is likely correctly developed in vessels without flow [106]. **(B)** A quasi-linear sigmoidal function models the relationship between WSS and Kir_*EC*_ open probability. **(C)** Analysis of the autoregulatory performance of the intact PA model. Purple curves are associated with a segment of the final PA model, where aEC respond to increased WSS caused by the rising ABNP. This response results in an increase in the open probability of EC Kir channels and the potentiation of eNOS-based signaling. NO produced by eNOS diffuses into the SMC, potentiating BK channels to enhance MT negative feedback and mitigate the elevated WSS. **(D)** Analysis of dysregulation with a 50% inhibition of primary ion channels in the PA model. **(E)** Vasodynamic analysis of the PA model under two different SMC $\tau_{mct}$ and three ABNP values, in response to an increase in EC extracellular potassium concentration from a baseline of 3 mM up to 7 mM in four discrete steps.

Fig 5A shows that, unlike the endothelium-denuded model, increasing the value of IP does not fully depolarize the SMC due to large Kir currents in coupled aEC. Consequently, SMC [Ca^2+^] does not exceed 150 nM, allowing passive distention to counteract the low contractile force of SMCs, which results in dilation at high IP values. In this pressure myography test, IP was increased with no blood flow in the vessel, resulting in zero WSS. The Kir current in aECs is also dependent on WSS, with WSS activating this channel [55]. At low mean arteriolar pressure, where blood flow is minimum and vessel diameter is maximum, WSS is minimum, nullifying the hyperpolarizing effect of EC Kir channels. As mean arteriolar pressure increases, WS-dependent currents in SMCs can constrict the vessel. As SMCs depolarize, the EC membrane potential also depolarizes due to gap junction coupling and small EC Kir channel activity under small WSS. With increasing SMC constriction force and vessel contraction, WSS rises which activates EC Kir channels; however, the EC is now less polarized due to coupled depolarized SMC. Therefore, Kir channel conductance is reduced at physiological extracellular potassium concentrations. Thus, EC Kir channel activity has a negligible effect on SMC membrane potential, allowing SMCs to autoregulate while maintaining EC Kir channels functional [55]. Increasing extracellular potassium of ECs by neuronally discharged potassium during FH causes a rightward and upward shift of the *I*_*Kir*_ curve [66], inducing EC hyperpolarization, which in turn hyperpolarizes SMCs.

To run simulations with this model, blood needs to flow into the vessel segment to exert WSS. This requires a circulatory network where blood flows through the network based on the IP gradient between its entry and outlet nodes. Taking a stepwise approach, we first integrated the endothelium-denuded segmented model of PA into the designed cerebral vascular model. Our earlier study demonstrated that, morphological differences exist between PA segments [43]. Superficial segments generally have larger diameters to accommodate higher blood flow and have higher expression of contractile elements to counteract passive distension for cerebral autoregulation. The relative expression of contractile elements in each segment was modeled by introducing a relative contractility index. Model parameters associated with the baseline diameter, passive distension, and expression of contractile elements (relative contractility) were tuned in the proposed endothelium-denuded segmented model, extending the segmented model to all 28 segments of each PA in the designed cerebral vasculature.

Once the endothelium-denuded PA model established, we set the VBNP to 10 mmHg and increased the ABNP from 40 to 130 mmHg in 10 mmHg increments, where PA segment diameters were automatically adjusted by the integrated endothelium-denuded model, while diameters of other segments, including surface arteries, sphincters, TZ, and capillaries, were manually optimized based on the myogenic response for each ABNP [43]. At ABNP = 130 mmHg, PA segments experience maximum WSS, as the blood flow peaks and vessel diameters are minimum. We quantified WSS values in all PA segments at ABNP = 130 mmHg and saved them as the maximum WSS (*WSS*_max_) of each segment. These *WSS*_max_ values were then used to model the modulation of EC Kir channel open probability in response to an imposed WSS in intact PA model, using a quasi-linear sigmoidal function(Fig 5B). In this intact PA model, the homocellular gap junctions between adjacent aSMCs and aECs have a resistance of 100 megaΩ [103,107,108].

To evaluate the developed intact PA model, we analyzed its performance in autoregulation. In our previous work, we showed that with optimal MT potentiation in all segments of our cerebral vasculature model, when ABNP increases within the autoregulation range, blood flow in non-bifurcating capillaries (Cap1 in Fig 1) remains around 0.5 nl/min. We also demonstrated that MT in TZ vessels and sphincters primarily autoregulates superficial blood flow, while MT in PAs plays a more pronounced role in deeper layers [43]. Building on this, we calculated the steady-state values of deep capillary (*L*4_Cap1_) blood flow and other model variables for a PA segment at a depth of 250 μm to assess the intact PA model’s autoregulation performance.

Adding the WSS-dependency of Kir channel activity to the endothelium-intact PA model (yellow curves) provides a similar autoregulatory performance to the endothelium-denuded model (blue curves) and also corrects the dysregulation observed in the absence of WSS-dependency in the EC Kir current (orange curves)(Fig 5C). Within the physiological ABNP range of 40–80 mmHg (corresponding to 30–65 mmHg IP within PA), in scenarios where MT is correctly adjusted in vessels (blue and yellow curves), blood flow in L4 non-bifurcating capillaries remains relatively constant. When ABNP increases above this range, even though the myogenic induced constriction in PA segments could not sustain plateaued autoregulation, *L*4_Cap1_ blood flow moderately increases, which is in agreement with the computational and experimental observations [43,60]. SMC membrane potential and [Ca^2+^] are also in the range reported in in-vitro studies [61]. The maximum MT level at ABNP = 130 mmHg is about 42% ($\frac{D_{\text{Pass}}-D_{\text{Act}}}{D_{\text{Pass}}}\times 100=\frac{21.5\;\mu \text{m}-12.6\;\mu \text{m}}{21.5\;\mu \text{m}}\times 100\approx 42\%$) for the analyzed PA segment, which matches the reported maximum MT in PAs[46].

WSS-dependent activation of eNOS is also known to inhibit MT in vessels [109]. In mouse PAs, this NO-induced dilation has been linked to increased SMC BK channel activity [46]. We adjusted the model parameters associated with the WSS/NO/BK pathway to reduce *MT*_max_ by approximately 14% compared to the case without the WSS-eNOS pathway (decreasing from 42% to 36%). As expected, when the WSS-NO pathway is included (purple curves) and the vessel is less constricted at the upper end of the autoregulation range, *L*4_Cap1_ blood flow increases at a higher rate. Additionally, while WS increases slightly compared to the case of no WSS-NO-BK pathway ($\frac{WS_{ABNP=130}}{WS_{ABNP=40}}$ changes from 1.75 to 1.9), WSS increases much less in the presence of the WSS-NO pathway ($\frac{WSS_{ABNP=130}}{WSS_{ABNP=40}}$ changes from 3.5 to 2.65). Within the autoregulation range, as mean arteriolar pressure increases, MT potentiation mitigates the WS increase associated with the rising ABNP, while MT inhibition via WSS mitigates the increased WSS. This regulation implies that vessels potentially adapt through both WS-dependent mitigation of WS via SMCs, and WSS-dependent mitigation of WSS via ECs, preventing excessive mechanical stress that could compromise vascular integrity and optimal function.

Next, we aimed to qualitatively demonstrate how disruption of different ion channels in our PA model can lead to cerebral dysregulation (Fig 5D). The simulation tested a 50% inhibition of various ion channels in the PA model. Focusing on the PA, and optimally adjusting other segments’ MT for autoregulation, the most dysregulated scenario occurs when Kir channels are disrupted in vascular cells, as SMC Kir current inhibition leads to more constricted vessels within the physiological pressure range (Fig 3F). Oxidative stress and inflammation in pathological conditions such as Alzheimer’s disease (AD) can impair Kir channel function [110–112], which might explain the hypoperfusion previously reported in AD mouse models [113,114]. As expected, the inhibition of TMEM16A channels and KV channels results in the hyperperfusion and hypoperfusion, pointing to their crucial roles in MT adjustment at different WS levels in aSMCs. Inhibition of BK channels also led to less WSS-dependent inhibition of MT in the upper range, and consequently, a smaller increase in blood flow.

After evaluating the intact PA model, we aimed to demonstrate that its EC Kir channels are functional in converting neural activity into vessel dilation. We addressed the question whether active neurons discharge potassium around ECs in specific vascular zones to hyperpolarize the endothelial network through gap junctions, or if all parenchymal ECs experience increased extracellular potassium during neural activity. In both scenarios, it seems logical that the EC basal membrane potential is mediated by MT in mural cells to maintain the autoregulation process. An increase in aEC [*K*^+^]_ex_, leads to endothelial network hyperpolarization in proportion to the amount of potassium released, which is subsequently transferred to SMCs, inducing vasodilation (Fig 5E). This proportionality is less evident in the case of ABNP = 50 mmHg due to the instability of both SMC and EC membrane potentials during the test, which continuously decline and impair the correlation between [*K*^+^]_ex_ and vasodilation. Under very low mean arterial brain pressure (ABNP = 50 mmHg), aSMCs operate at low IP (30–40 mmHg depending on cortical depth), small WS-depolarizing currents, and large RMR (Fig 3D and 3E), which makes them sensitive to minor changes in the dominant SMC Kir currents. The negative slope region of the Kir channel I-V curve explains this continuous downward trend (Fig 3D - green curve). A slight initial hyperpolarization can trigger a positive feedback loop, increasing Kir channel conductance and driving the membrane potential further toward negative values until the Kir current saturates at the peak of the negative slope region. This persistent hyperpolarization and vasodilation might serve as a protective mechanism under low mean arterial pressure, maximizes vessel dilation, and increases arterial pressure to counteract the low perfusion.

### Section 2: Modeling the kinetics of vasomotion

Vasomotion, characterized by low-frequency, undamped oscillations of arteriolar diameter, is a feature of cerebral vasculature dynamics observed during the resting state [115–117]. In this state, neural activity is limited to baseline levels, and we assumed that the myogenic response in arterioles is facilitated by the tonic release of mediators from various brain cells, including NPY and other signaling molecules.

**Fig 6:**
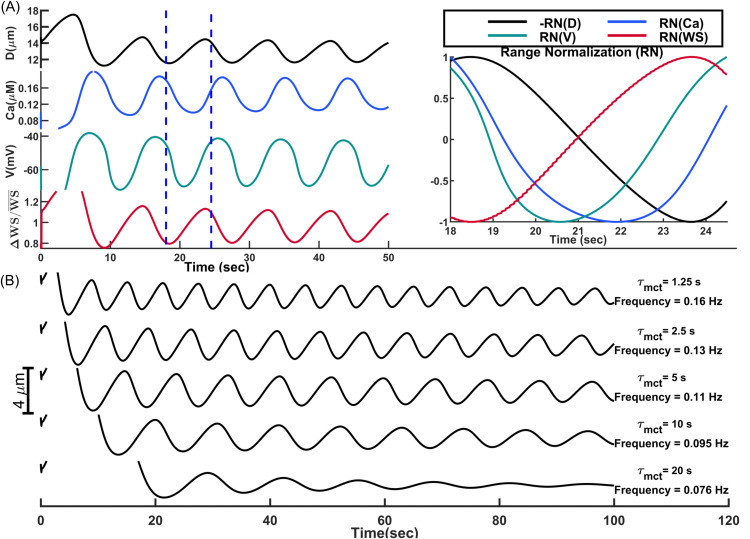
Oscillations of a PA diameter induced by the delayed myogenic responses. **(A)** Left: Oscillations of the PA segment diameter, SMC [Ca^2+^] and membrane potential, and relative changes of WS. Right: Range-normalized view of oscillations between 18-24.5 sec (the period between blue dashed area in the left panel). The diameter curve is inverted for better visualization of time lags. **(B)** Analysis of oscillation frequency and amplitude for various mechanotransduction time constants ($\tau_{mct}$).

A delayed negative feedback, including the delay in the myogenic response, can induce oscillations. When WS in a vessel segment increases, the myogenic response triggers the muscular constriction to counteract the elevated WS. Delayed readjustment of the muscular force generates an overshoot in the system’s response which deviates WS in the opposite direction. The repeating cycles of overshoots and delayed corrections generate oscillations (Fig 6A). Variations in the vessel diameter cause changes in the exerted WS and hemodynamics, and with some delay, modulate the SMC membrane potential. Range-normalized curves reveal a 2 sec delay between the WS trough and the SMC membrane potential trough in our model (t = 18.5 sec vs. t = 20.5 sec (Fig 6A, right panel)). The adjustment of the SMC [Ca^2+^] via VOCCs lags behind changes in its membrane potential due to the time required for Ca^2+^ ions to enter or exit the cell until [Ca^2+^] is tuned to the membrane potential. In our model, this delay is about 1.5 sec (t = 20.5 sec vs. t = 22 sec). The delay in the adjustment of MLC phosphorylation in response to changes in [Ca^2+^] further delays the adjustment of SMC constriction and vessel diameter, resulting in a 1.7 sec delay between the vessel diameter and SMC [Ca^2+^] curves (t = 22 sec vs. t = 23.7 sec). This timing aligns with the time-lag observed in-vivo between vessel diameter and SMC [Ca^2+^] changes in awake mice [80]. Overall, there is a cumulative 5.2 sec delay between the moment the vessel segment experiences the minimum WS and the time the SMC constriction force reaches to its minimum which is the end of the dilation phase. A similar dynamic occurs in the opposite direction, but with faster Ca^2+^ dynamics during constriction, resulting in a 4.2 sec lag between the maximum WS and maximum SMC constriction force which is the end of the constriction phase. This results in a 9.4 sec full oscillation period, or the frequency of about 0.1 Hz. These results show how a delayed myogenic response can generate oscillations in the PA diameter.

Vasomotion oscillations in a PA segment were analyzed for various $\tau_{mct}$ values (Fig 6B). As expected, decreasing $\tau_{mct}$ via increasing NPY concentration (facilitation of the myogenic response) reduces the interval between the maximum WS and the peak of constriction force, and increases oscillation frequency, consistent with experimental observations [118]. In this case, the oscillation amplitude is reduced since the system rapidly corrects any deviations from the target state. The amplitude of vasomotion observed for $\tau_{mct}$=1.25 sec is smaller than the amplitude of oscillations when $\tau_{mct}$=5 sec (Fig 6B).

Arteriolar oscillations in the PA model ultimately dampen over time. Under fast myogenic response conditions (small $\tau_{mct}$), these oscillations fully dampen after 2–3 minutes. When the response is slower (e.g., $\tau_{mct}$=20 sec), the damping occurs even sooner, within the first minute. Sustained oscillations are observed in the cerebral vasculature in-vivo, indicating that additional factors contribute to maintaining oscillations. To show how these vessels generate synchronized undamped oscillations, we should model PAs as a network of coupled oscillators. In a system of coupled oscillators, synchronized oscillations in adjacent oscillators (PAs) can reinforce each other, sustaining the overall oscillatory activity. For computational efficiency, our simulations so far have modeled only a single PA using cellular-based coupled segments, while keeping all other segments in the cerebrovascular network at a fixed diameter. As a result, the stabilization of proximal hemodynamics led to a gradual damping of oscillations in the analyzed PA. This damping occurs because sustained vasomotion relies on synchronized fluctuations in hemodynamics across the vascular network, which requires coupling between arteriolar segments throughout the system. To simulate this, we developed a computationally efficient non-cellular simplified macro-scale model for all arteriolar segments based on insights gained from the cellular PA model. Then, we used the new simplified model to investigate the effect of network synchronization in sustaining vasomotions.

In the next set of simulations, we initially assigned an ABNP of 60 mmHg to the cerebrovasculature model and adjusted the diameters of all segments to achieve cerebral autoregulation at this ABNP value ([43]). Based on the IP distribution in the network and the assigned diameters, the initial WS in arteriolar segments (AS) was calculated using:


$$WS(AS,i=0)=\frac{D(AS,i=0)\cdot IP(AS,i=0)}{h(AS)}$$ (1)

These values, which serve as the baseline around which the WS in arteriolar segments oscillates under an ABNP of 60 mmHg in the cerebrovasculature model, were stored as the *WS*_*avg*_(*AS*) vector, assuming a constant vessel thickness *h*(*AS*). We further assumed that at this ABNP level, the MT is equal across all arteriolar segments, which results in a hypothetical SMC membrane potential of -45 mV (V(AS,i=0)=−45) in all segments. With these initial values set, the simulation loop was initiated with a time step of *dt* = 0.2 sec, where *i* denotes the iteration number. In each iteration, the diameter of each segment was updated according to the macro-scale arteriolar segment model described below. A hemodynamic analysis was then conducted to calculate the new hemodynamics based on updated vessel diameters for the subsequent step. This recursive process was repeated for 600 seconds.

To model vasodynamics in this approach, diameters of arteriolar segments were updated in each iteration based on the delayed myogenic response, passive distension, and the coupling between adjacent segments. Based on the results of Fig 6A, if the WS of a segment exceeds *WS*_*avg*_, the myogenic response is triggered to correct this deviation, albeit with a delay. Consequently, the real-time SMC membrane potential of an arteriolar segment is proportional to the difference between its WS from several seconds earlier and the segment’s *WS*_*avg*_:


$$V_{\text{init}}(AS,i)=-45+MR_{F}\cdot \left(WS(AS,i-\frac{MR_{D}}{dt})-WS_{avg}\right),\;\text{for }i>0$$ (2)

Here, *V*_init_(*AS*,*i*) represents the initial membrane potential of each arteriolar segment before coupling to adjacent segments. *MR*_*F*_ is the myogenic response factor, and *MR*_*D*_ denotes the myogenic response delay, which was set to 5 sec in our model. As shown in Fig 6A, part of the delay in the myogenic response is attributed to the lag between changes in SMC membrane potential and [Ca^2+^], as well as between changes in [Ca^2+^] and adjustments in SMC constriction force. However, in this macro-scale model, where a vessel diameter is adjusted based on the real-time SMC membrane potential, all these delays were encapsulated in *MR*_*D*_. This means that from the moment WS exceeds *WS*_*avg*_, until the vessel stops dilating, there is a delay of *MR*_*D*_ sec.

The SMC membrane potential of each segment is influenced not only by its own hemodynamics but also by coupling with adjacent segments. In reality, this coupling occurs through gap junctions, where currents flow based on membrane potential differences, similar to how voltage distributes across resistive-capacitive (RC) circuits. To capture the gradual influence of neighboring segments’ membrane potentials on each other and simulate real-time RC interactions via electrical coupling in our macro-scale model, we implemented a secondary iterative loop within each simulation step. In each iteration of the secondary loop, we computed the gap junction current (*I*_gap_) for each arteriolar segment, progressively updating the segment’s membrane potential in an RC circuit-like manner. At the end of the secondary loop, the final stabilized coupled membrane potential (*V*_final_(*AS*,*i*)) was obtained from the initial uncoupled membrane potential (*V*_init_(*AS*,*i*)). The myogenic response (MR) portion of the segment’s diameter was then determined by comparing the segment’s membrane potential to -45 mV:


$$D_{\text{MR}}(AS,i)=-45-V_{\text{final}}(AS,i)$$ (3)

If the final membrane potential of a segment is larger than -45 mV, this means that the WS in that segment (and in surrounding segments due to the coupling) 5 sec ago was above its average value. Consequently, the MR portion of the segment’s diameter becomes negative, leading to a decrease in diameter to counteract the increased WS and bring it closer to the average. Conversely, if the final membrane potential is less than -45 mV, the opposite process occurs.

The passive distension (PD) portion of the segment’s diameter was calculated based on the real-time WS experienced by each segment using the following equation:


$$D_{\text{PD}}(AS,i)=D_{WS=0}\cdot (1+\alpha \cdot (1-e^{-K\cdot WS(AS,i)}))$$ (4)

In this equation, *K* represents the rate at which the vessel distends as WS increases, and the parameter α determines the maximum magnitude of passive vessel distension achievable at large WS values. The diameter of each segment at each main iteration step was then calculated by adding the passive distension portion of the segment’s diameter (*D*_PD_) and the myogenic response portion of the segment’s diameter (*D*_MR_):


$$D(AS,i)=D_{\text{PD}}(AS,i)+D_{\text{MR}}(AS,i)$$ (5)

**Fig 7:**
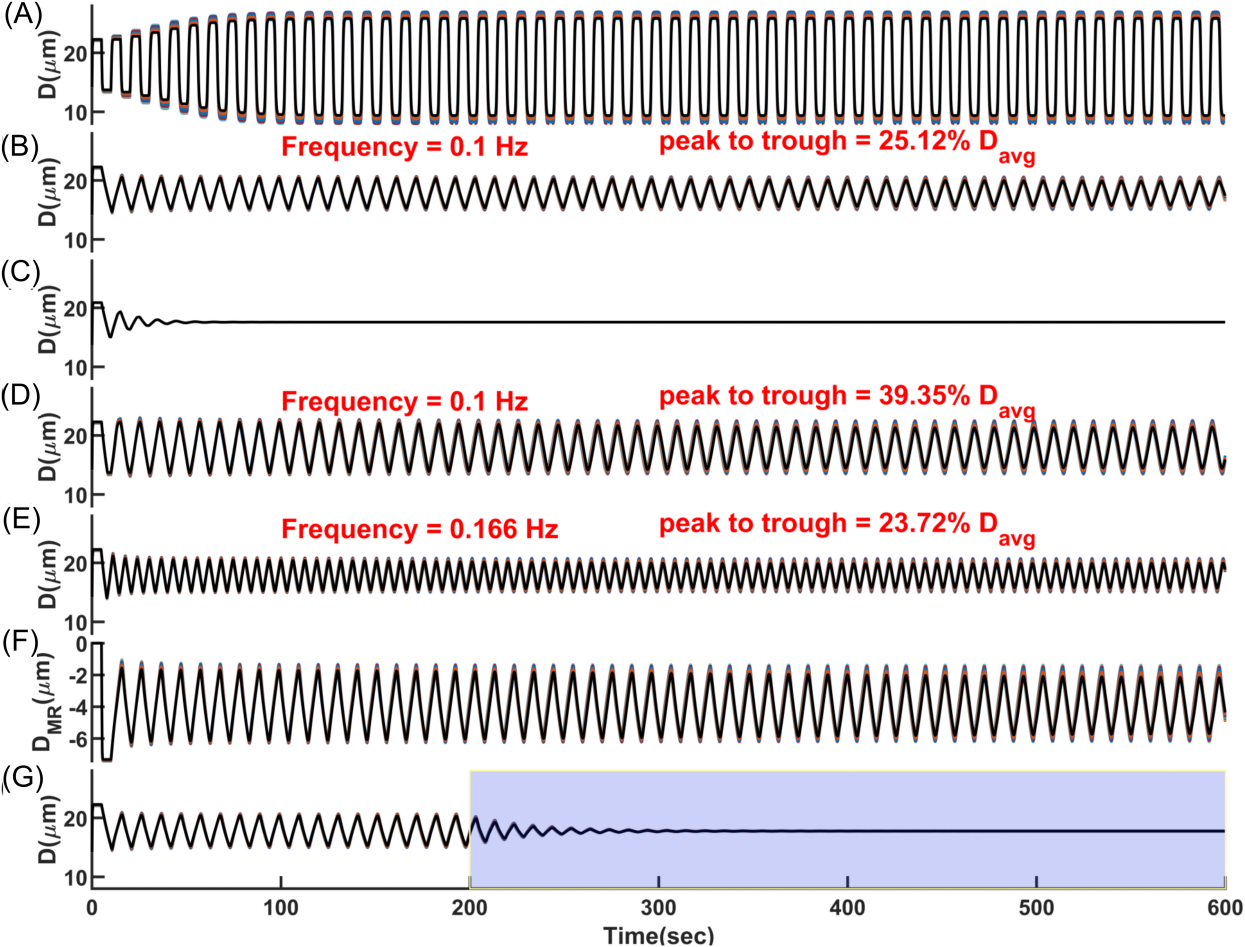
Synchronized vasomotion in a network of 1009 coupled arteriolar segments of the cerebrovascular model, where the diameter of each segment was modeled based on delayed myogenic response (*MR*_*D*_ = 5 s) and passive distension. Curves represent synchronized vasomotion in 10 arteriolar segments with approximately equal diameters from different PAs, overlaid. **(A)** Square-wave oscillatory pattern with a large amplitude, assuming no constraints on the arteriolar diameter change rate in the model. **(B)** Triangular and symmetric oscillatory pattern with an amplitude of 25% of the mean diameter, assuming different maximum allowable diameter change rates during dilation and constriction phases (15% per sec for dilation and 7.5% per sec for constriction). **(C)** Dampened oscillations when the macro-model was implemented only in segments of one PA, rather than in a coupled PA network through pial arteries. **(D)** Triangular and symmetric oscillatory pattern with an amplitude of 40% of the mean diameter, assuming equal and large maximum allowable diameter change rates during dilation and constriction (15% per sec). **(E)** Maximum diameter rate change and the delay in the myogenic response are the primary variables modulating vasomotion amplitude and frequency. Larger allowable diameter change rate with a faster myogenic response (*D*_*MR*_ = 3 s) results in a moderate oscillation amplitude (24% of the mean diameter). **(F)** Myogenic response portion of diameter oscillations in **(B)**. These are negative values as the myogenic response induces constriction to counter passive distension, with constriction periodically potentiated and inhibited. Comparing peak-to-trough diameter oscillations with myogenic response oscillations shows that about 15% of oscillation amplitude is attributed to passive distension. **(G)** Passive distension plays a key role in sustaining vasomotion. Keeping the passive distension portion of the diameter constant from *t* = 200 sec (highlighted interval) results in dampened oscillations.

The algorithm corresponding to this simplified model and the model parameter values are provided in the Methods section. Fig 7A shows the oscillatory dynamics of segments at the same cortical depth across 10 different PAs within the simulated vasculature. The oscillations have a square-wave pattern resulting from the vessels alternating between phases of distension and constriction. After the initial passive distension, the vessels remain distended for a fixed period because the initiation of potentiation of MT is delayed. Once this delay elapses, MT increases instantaneously leading to rapid constriction. As WS decreases and crosses below the average threshold, there is another delay before the MT inhibition begins. During this delay, vessels remain constricted despite the small WS. After this delay, MT decreases instantaneously, causing the vessels to dilate quickly. These abrupt transitions, combined with the fixed delays before MT adjustments, produce the distinct square-wave vasodynamics observed.

In reality, however, MT does not change instantaneously since SMC membrane potential, [Ca^2+^], and the ratio of phosphorylated MLC molecules change slowly over time (see Fig 6A). The fastest observed rate of arteriolar dilation during vasomotion in awake mouse PAs is approximately 10–15% per second ([80]), while the fastest constriction rate was reported to be half of that [119]. To replicate this physiological behavior in our model, we implemented constraints that limit the rate of diameter change for each vessel segment during both dilation (15% per sec) and constriction (7.5% per sec) (Algorithm 2 in the Methods section).

Fig 7B displays the oscillatory behavior of PAs after applying constraints to the diameter change rate. As shown, all PAs synchronously oscillate in a triangular pattern, indicating that synchronization is a dominant feature in these oscillations. This contrasts with the sinusoidal and dampened oscillations observed in the cellular-based PA model depicted in Fig 6A. As previously mentioned, when diameters of pial arteries and other PA segments are held constant in the cerebrovasculature model, the hemodynamics in the pial network stabilize, leading to damping of oscillations in the simulated PA. Similarly, Fig 7C shows that implementing the macro-scale model in only one PA within the cerebrovascular model also results in dampened oscillations. These findings show that for the oscillations to sustain, synchronized hemodynamic fluctuations and synchronized changes in SMC membrane potentials are required. These synchronized activities reinforce the oscillations throughout the network.

As depicted in Fig 7B, even though the maximum allowable diameter change rates during the dilation and constriction phases were set differently, the vasomotion still has a symmetric triangular pattern. The maximum diameter change rate does not exceed the lower limit of 7.5% per sec. This occurs because the rate of constriction or dilation governs how quickly WS decreases or increases. Consequently, the inhibition or potentiation of the MR also follows the slower rate of constriction or dilation. This prevents any exceedance of the 7.5% per sec limit, even though the model allows for a faster dilation rate. Therefore, the smaller rate of vessel constriction or dilation determines the rate of both phases, leading to a symmetric triangular vasomotion pattern in synchronized vessels. Under this 7.5% per sec constriction rate, the peak-to-trough vasomotion amplitude is approximately 25% of *D*_*avg*_ in our model.

Fig 7D shows that when the maximum allowable diameter change rates were set equal and large in both dilation and constriction phases (15% per sec), the resulting vasomotion amplitude is larger, with a peak-to-trough change of approximately 40% of *D*_*avg*_. Faster diameter change rates allow vessels to overshoot or undershoot more sharply during the delay period, leading to larger oscillation amplitudes. However, in the physiological vascular system, an increase in the diameter change rate may be accompanied by a decrease in the MR delay, unlike in our model where these variables are independent. This is because faster diameter changes imply quicker changes in SMC [Ca^2+^] and MLC phosphorylation, which are processes that also influence the MR delay. A reduced MR delay limits the vasomotion amplitude, as quicker feedback reduces the time available for energy buildup and overshoot within each cycle (Fig 6B). Fig 7E displays vasomotion with an allowable maximum diameter change rate of 15% per sec for both dilation and constriction, while the MR delay was set to 3 sec. As shown, the amplitude of vasomotion is approximately 24% of *D*_*avg*_. These simulations show that increasing the vessel diameter change rate, when accompanied by a decrease in the MR delay, and vice versa, have opposing effects on the vasomotion amplitude. This balance helps maintain the vasomotion amplitude within a desired range to prevent excessive vessel constriction or dilation.

While we showed that hemodynamic-vasodynamic interactions can lead to sustained oscillations, we did not emphasize the role of wall mechanics in this dynamic. Delayed negative feedback can initiate oscillations, but these oscillations may not persist indefinitely due to inherent damping mechanisms and energy losses in real physiological systems. To maintain sustained oscillations, an amplifying mechanism is required to compensate these losses. For example, in previous vasomotion models, amplification was achieved through intracellular processes like calcium-induced calcium release (CICR) in SMCs, creating an active biochemical positive feedback loop [115,120]. In our vasomotion model, we propose a mechanical amplification mechanism: when SMCs relax, passive vessel wall distension increases the diameter and amplifies deviations in wall stress, which are then corrected by the delayed myogenic response; when SMCs constrict, the opposite occurs.

Fig 7F shows the MR portion of diameter changes associated with the vasomotion displayed in Fig 7B. While the total oscillation amplitude in a vessel segment with an average diameter of about 17.5 μm is approximately 4.5 μm, the MR portion accounts for 3.85 µm (85%), indicating that 0.65 μm (15%) of the oscillation amplitude is attributed to passive distension in the model. Fig 7G displays the vasomotion pattern for *t* > 200 sec when the passive distension portion of vessel diameter was held constant at its value at *t* = 200 sec. As shown, oscillations driven solely by delayed nega*t*ive feedback start damping immediately. During the first dilation phase for *t* >= 200 sec, the absence of passive distension results in less pronounced dila*t*ion and thus smaller deviations in wall stress, leading to a weaker subsequent constriction. This dampening dynamic continues un*t*il the vessel diameter stabilizes completely. Collectively, the simulation results in Fig 7 demonstrate that sustained vasomotion in coupled PAs emerge from the dynamic interplay between delayed myogenic response and passive wall mechanics, which together amplify wall-stress deviations and maintain oscillatory behavior.

**Fig 8:**
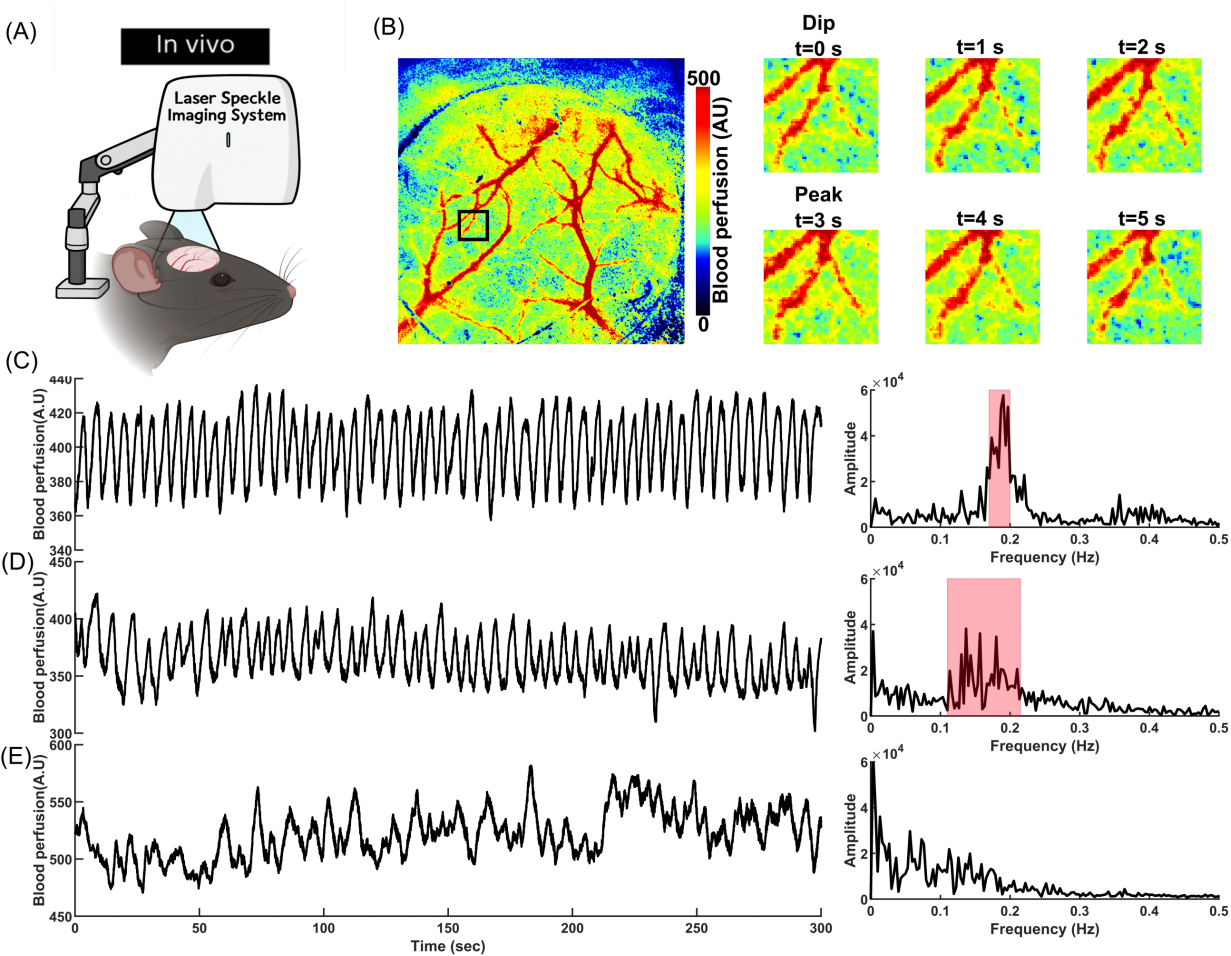
In-vivo analysis of flowmotion characteristics in ketamine-dexmedetomidine anesthetized mice. **(A)** Schematic representation of in-vivo flowmotion measurements using LSCI. Created in BioRender. **(B)** Left: An example of a recorded LSCI signal at a specific time near the left middle cerebral artery (MCA) in the somatosensory cortex of a mouse. Right: Spatiotemporal changes in the LSCI signal during one cycle of flowmotion within the ROI depicted on the left. **(C, D)** Two examples of LSCI signals where flowmotion exhibits a triangular oscillatory pattern. Even in such triangular flowmotion, there is no dominant oscillatory frequency. **(E)** A typical recorded flowmotion signal.

To provide in-vivo support for the proposed vasomotion model, we used laser speckle contrast imaging (LSCI) to analyze flowmotion in the somatosensory cortex of anesthetized mice (Fig 8A). Flowmotion refers to oscillations in blood flow that originate from vasomotion and have similar characteristics. Five minutes of high-temporal-resolution LSCI signals were recorded from different animals. Fig 8B shows a color-coded LSCI signal in the highlighted region of interest (ROI) for one cycle of flowmotion, which was then spatially averaged across the entire ROI to extract the flowmotion signal, including examples from different animals (Fig 8C–8E). In our in-vivo experiments, we rarely observed flowmotion with a complete triangular pattern (Fig 8C and 8D). This pattern is the characteristic of synchronized, vascular-centric vasomotion generation with minimal interference from neurogenic or astrocytic responses—or phase-locked oscillations of neurogenic, astrocytic, and myogenic activity [26,121]. These dynamics suggest that, at the time of recording, the animal was deeply anesthetized, and NO production suppressed [122]. NO dampens the myogenic response, and its suppression facilitates the interaction between passive and myogenic responses required for oscillation generation. This facilitated myogenic response may also explain the 0.18–0.2 Hz vasomotion frequency observed in Fig 8C, approximately twice the commonly reported 0.1 Hz vasomotion frequency in awake mice [32,123]. Several factors within the mouse brain vasculature can continuously modulate the myogenic response dynamics, including SMC mechantransduction time constant, intracellular Ca^2+^ dynamics, and the sensitivity of MLC phosphorylation to [Ca^2+^]. These factors collectively determine the diameter change rate and MR delay, which can continuously alter the frequency and amplitude of vasomotion (Fig 7). As shown in Fig 8D, within five minutes of recording, multiple dominant frequencies of oscillations emerged in the flowmotion signal. This suggests that, in the dynamic environment of the cerebral vasculature, we should not expect a single dominant frequency arising from an unchanged delayed myogenic response in the network. In our in-vivo experiments, we rarely observed full oscillations, and flowdynamics were generally stochastic (Fig 8E). To investigate the primary drivers of these stochastic flowdynamics, we introduce additional layers to the model.

### Section 3: Modeling NGVC-driven arteriolar vasodynamics

In this section, we first simulate the interplay between passive, myogenic, and neurogenic responses by implementing the proposed NVC model and validate its predictions against experimental data. Subsequently, we examine the role of astrocytes in modulating arteriolar vasodynamics by comparing NVC model-predicted vasodynamics with in-vivo recordings from the awake mouse brain.

#### Modeling arteriolar vasodynamics in response to neuronal impulse activity.

To refine the proposed computational model to better reflect physiology, we incorporated in-vivo experimental data. The essential experimental data for our analysis in this section were sourced from in-vivo vasodynamic recordings derived from 0.5 sec optogenetic (OG) stimulation, captured using two-photon microscopy from the somatosensory cortex of mice [124]. Previous studies have shown that OG-evoked vasodilations predominantly initiate below cortical layer IV [125–127]. Thus, analyzing the upward propagation of OG-evoked vasodynamics from deeper to more superficial cortical layers provides a valuable lens to examine the impact of neuronal impulse input on vasodynamics across different cortical depths. This approach allows us to identify depth-dependent deterministic features in neurogenic-myogenic interactions, which can be validated against the predictions of our proposed model to evaluate its accuracy.

**Fig 9:**
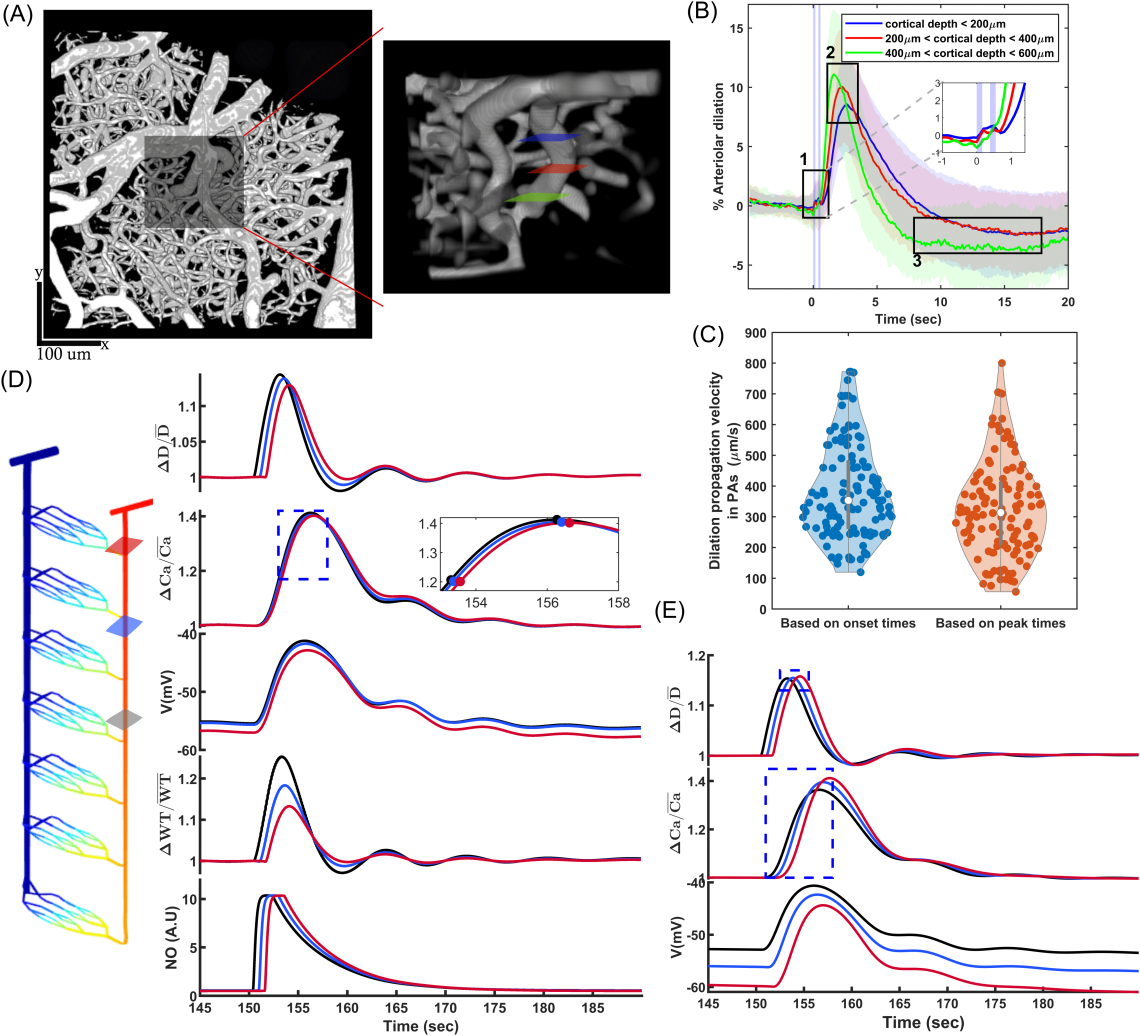
Neurogenic impulse response of the CBF regulatory system: (A-C) OG-evoked vascular reactivity in PAs of awake mouse brain across different cortical depths, sourced from [124,125]. (A-left) Example of a two-photon image of the mouse somatosensory cortex vasculature. (A-right) Depth-resolved illustration of the ROI shown on the left. **(B)** Averaged OG-evoked arteriolar vasodynamics measured in three different cortical depth regions (colored cross-sections in (A-right)), highlighting three depth-dependent features of the in-vivo OG-evoked vasodynamics: 1. Delayed arteriolar vasodilation in superficial layers, 2. Attenuated arteriolar dilation in superficial layers, and 3. More pronounced post-stimulus constrictions in deeper layers. **(C)** Violin plot of the calculated speed of vasodilation propagation along each PA in the dataset, determined using onset-to-onset and peak-to-peak times from concurrently recorded vasodynamics across different depths, assuming PAs penetrate perpendicularly into the tissue. **(D)** Left: Modeled PA consisting of 28 coupled segments, each including EC, SMC, and passive distension models. Right: Spatiotemporal changes in model parameters at three cross-sections of the PA, located at depths of 405, 225, and 45 μm, in response to instantaneous neuronal activity. The dots in the zoomed-in area represent timestamps used for calculating Ca^2+^ wave propagation and electrical signaling. **(E)** Spatiotemporal changes in model parameters in response to instantaneous neuronal activity, assuming there exists no electrical coupling between PA segments (removal of gap junctions), resulting in inaccurate predictions of vasodynamics compared to in-vivo recordings.

Brief OG stimulation triggers delayed and attenuated arteriolar dilations in superficial cortical layers compared to deeper ones, with a more pronounced post-stimulus undershoot in the deeper layers ([125,128]) (Fig 9A and 9B). We quantified the propagation speed of arteriolar dilation by measuring the onset and peak times of dilation at different cortical depths of each PA (as detailed in Methods), and identified vasodilation propagation speeds of 300–400 μm/sec (Fig 9C), significantly slower than the retrograde electrical propagation speed of 2 mm/sec reported by [10]. nNOS-expressing interneurons evoke arterial dilation in the somatosensory cortex of mice [129], and the delayed vasodilation in superficial arterioles might result from slower neurovascular communication kinetics in superficial layers rather than from the delayed propagation of neural activity from the deeper layers to the superficial area [125,128]. Taking these observations together, we assumed, as a hypothesis, that instantaneous neuronal activity leads to the release of equal amounts of neuronally produced NO across all cortical depths, with delayed modulation of the myogenic response and vasodilation in superficial layers due to slower neurovascular communication kinetics. We then evaluated whether this scenario could recapitulate the depth-dependent deterministic features observed in PA vasodynamics, including the attenuated vasodilation during upward propagation and the more pronounced post-stimulus undershoot in deeper layers.

We initially set the ABNP of the cerebrovascular model to 60 mmHg, and we allowed the oscillations to fully damp before applying neurogenic input to the cellular-level PA, allowing us to analyze the average neurogenic impulse response of the PA, without interference from oscillations. We then assumed that instantaneous neuronal activity induced a sudden rise in NO concentration in PA segments located below 600 μm depth, whereas in PA segments above 600 μm, this rise propagated at a speed of 400 μm/sec. This delayed adjustment was designed to capture the depth-dependent onset of vasodilation observed in Fig 9B.

Fig 9D shows the changes in various parameters of the analyzed PA at three different depths. As shown in the bottom panel, the NO/cGMP concentrations were modeled to increase with a delay corresponding to cortical depth and to decay exponentially with a 5 sec time constant. The NO/cGMP/PKG pathway primarily induces rapid vasodilation by activating PKG, which in turn activates MLCP and desensitizes MLC to Ca^2+^ which leads to partial dampening of the myogenic response (Section 1). Therefore, when the PA segment located at 405 μm (black curves) experiences this NO-induced vasodilation, the damped myogenic response is followed by an increase in vessel segment diameter and elevated IP during the dilation phase, which increases the WS and potentiates the myogenic response. This, in turn, causes a subsequent increase in SMC membrane potential and SMC [Ca^2+^]. Due to the electrical coupling between vascular cells, the increased SMC membrane potential in deeper PA segments, which experience the rise in NO concentration earlier, is transmitted to the upper vascular cells via gap junctions, causing rapid depolarization in the upper layers’ SMCs even before the NO concentration rises there. Based on the timing of the mid and peak [Ca^2+^] increases in analyzed segments, the electrical propagation speed in our model was approximately 1200 μm/s. In-vivo findings showed that during instantaneous sensory stimulation, the propagation of vasodilation in superficial vessels lagged behind the rise in SMC [Ca^2+^], indicating that vasodilation occurred at a speed of around 400 μm/sec, consistent with the values calculated in Fig 9C, where the SMC Ca^2+^ wave was propagating at approximately 1200 μm/sec [130].

During the time interval when SMC [Ca^2+^] is increasing almost simultaneously across all segments but before reaching the peak, the NO/cGMP concentration is higher in the deeper layers due to the earlier rise. As a result, the dilation of deeper PA segments during this phase is more pronounced than in the superficial segments. In the superficial segments, the equal concentration of NO/cGMP is accompanied by a more potentiated myogenic response evoked by earlier dilations in deeper segments, which is quickly transmitted electrically upstream (Fig 9D, top panel). This dynamic explains the attenuation observed in the upward propagation of vasodilation observed experimentally (Fig 9B). Furthermore, the potentiated myogenic response results in rapid vasoconstriction following the initial NO-induced partial dampening of the myogenic response, rather than the vasodynamic response that follows the slow degradation of NO/cGMP. Therefore, during the interval when SMC [Ca^2+^] is near its peak in all segments, the NO/cGMP concentration in deeper cortical layers is lower due to its earlier degradation following the initial rise. At this interval, the increased MLC Ca^2+^ sensitivity in the deeper segments leads to a more pronounced post-stimulus undershoot. This dynamic also explains the more pronounced post-stimulation undershoot observed in the deeper segments experimentally (Fig 9B).

Fig 9E shows the simulation results under similar neurogenic input, but without coupling between adjacent vascular cells in the modeled PA. The basal SMC membrane potential at different depths is not within the same range as it was in the coupled segments scenario depicted in Fig 9D. Additionally, the myogenic response begins to potentiate independently in each PA segment following its delayed NO-induced vasodilation, rather than in a synchronized manner, despite the delay in dilation. In this simulation, the depth-dependent deterministic features observed experimentally and computationally are absent. Therefore, Fig 9 collectively demonstrates that the observed experimental features can be recapitulated by a scenario in which instantaneous neuronal activity triggers NO production to act on SMCs, momentarily dampening the myogenic response and inducing vasodilation. In this scenario, the NO-induced dilation subsequently potentiates the myogenic response, leading to the attenuation of the initial vasodilation and the emergence of a post-stimulus undershoot in PA segments. Depending on the cortical depth of the PA, the degree of attenuation in the initial vasodilation and the magnitude of the post-stimulus undershoot vary.

**Fig 10:**
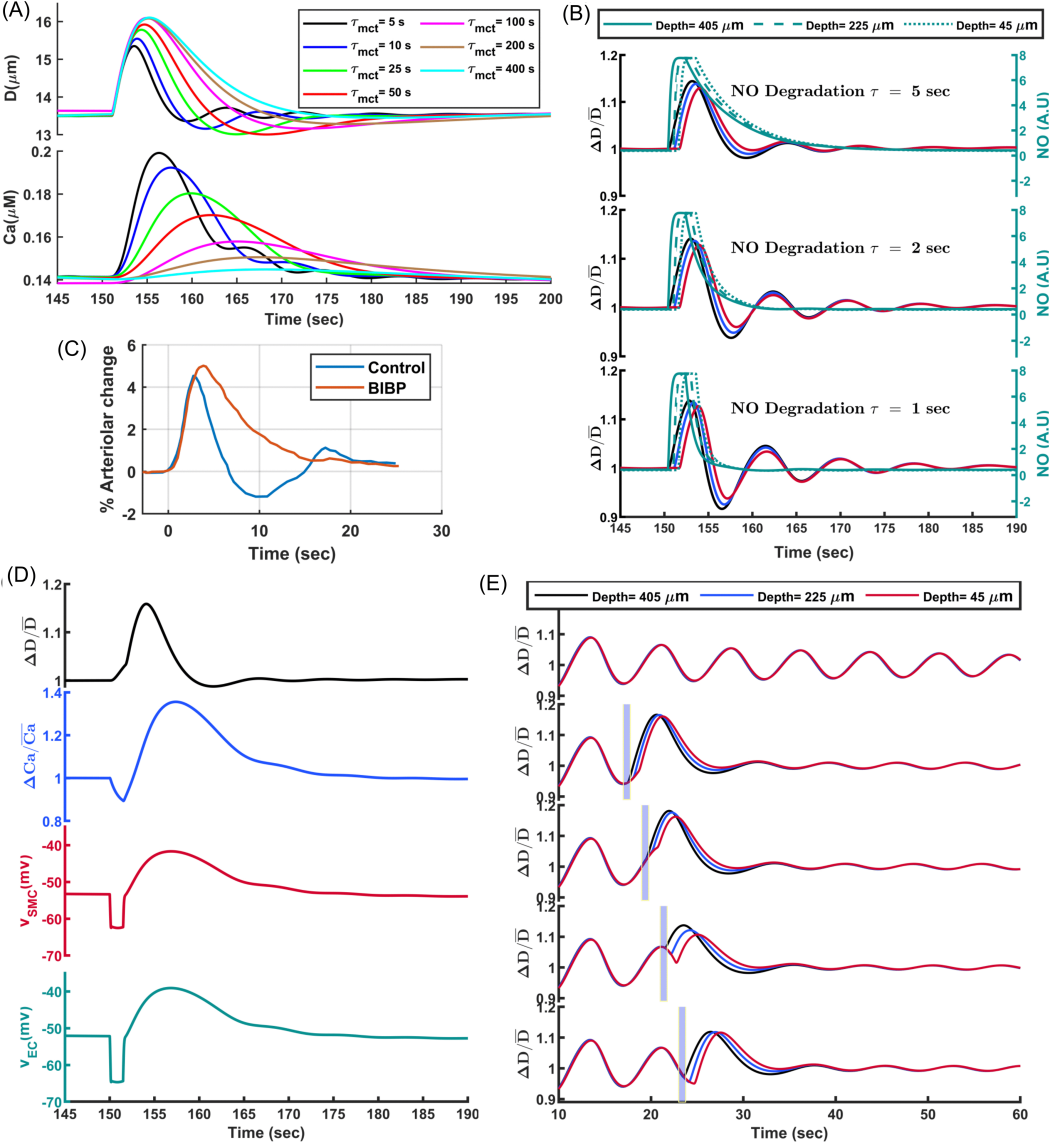
Analyzing arteriolar vasodynamics in response to neuronal impulse input under various model parameter values. **(A)** Vasodynamics and SMC Ca^2+^ dynamics under various SMC mechanotransduction time constant ($\tau_{mct}$). Decreasing $\tau_{mct}$ leads to a more attenuated vasodilation, along with a shorter duration of vasodilation and post-stimulus undershoot. **(B)** Predicted vasodynamics under various NO/cGMP degradation time constants. Faster degradation results in more pronounced post-stimulus constriction and oscillatory behavior, which is absent in in-vivo signals, suggesting that NO/cGMP degradation occurs slowly under physiological conditions. **(C)** In-vivo recorded vasodynamics from Uhlirova’s study [125], showing significant changes in vasodynamics upon the application of the Y1 receptor blocker BIBP 3226. Data were digitized from Uhlirova’s study and replotted here, under the terms of the CC BY 4.0 license (https://creativecommons.org/licenses/by/4.0/). **(D)** Model-predicted vasodynamics assuming that, during instantaneous neuronal activity, neuronally released mediators also hyperpolarize vascular cells, leading to biphasic SMC [Ca^2+^] and larger vasodilation. **(E)** Model-predicted vasodynamics based on the assumption that instantaneous neuronal activity occurs at four different phases of ongoing vasomotion.

Next, we analyzed the effects of different $\tau_{mct}$ values on vasodynamics in response to neuronal impulse input. Increasing $\tau_{mct}$—which corresponds to lower activation of SMCs’ NPYRs—resulted in a slower myogenic response, where adjustments allowed a less immediate and pronounced reaction to changes in the diameter and WS (Fig 10A). Consequently, the initial dilation induced by NO-dependent partial dampening of the myogenic response was smallest with the fastest myogenic response and largest with the slowest. However, the magnitude of the post-stimulus undershoot depends not only on the degree of the potentiated myogenic response, which is more pronounced with smaller $\tau_{mct}$, but also on the degradation rate of NO/cGMP molecules. The post-stimulus undershoot was most pronounced at $\tau_{mct}$ values of 25 and 50 secs, compared to both smaller and larger $\tau_{mct}$ values. The reason is, within this $\tau_{mct}$ range, and with a 5 sec time constant for the exponential decay of NO/cGMP concentration, the increased Ca^2+^ sensitivity of MLC from 10 to 15 sec after the initial NO/cGMP rise coincides with a delayed myogenic response-induced Ca^2+^ peak. This, in turn, resulted in a more pronounced post-stimulus undershoot compared to faster myogenic response scenarios, where even larger Ca^2+^ peaks occurred within the first 10 sec, when MLC sensitivity to Ca^2+^ was reduced due to higher NO/cGMP concentration. Furthermore, the analyzed dynamics shows that both the duration of the initial vasodilation and the post-stimulus undershoot increase with increasing $\tau_{mct}$, indicating a slower myogenic response. These modeled and comparative dynamics were similarly observed in instantaneous sensory-evoked vasodynamics in adult and aged mice [131]. Based on the reported vasodynamics and concurrent SMC Ca^2+^ monitoring in that study, and the vasodynamics we analyzed under various $\tau_{mct}$ values in Fig 10A, we can conclude that the myogenic response in aged mice is slower than in adults, since the duration of both vasodilation and the myogenic response-induced elevation of SMC [Ca^2+^] were longer in aged mice.

Next, we analyzed vasodynamics in response to neuronal impulse input with a fixed $\tau_{mct}$ of 5 sec, while changing the time constant of the assumed NO/cGMP concentration exponential decay. As shown in Fig 10B, reducing the time constant, which accelerates the decay of NO/cGMP concentration, affected the duration of vasodilation, the degree of post-stimulus undershoot, and the timing of its occurrence. Comparing these simulation results with the experimental data shown in Fig 9B suggests that a slower decay in NO/cGMP concentration, with τ=5 sec degradation time constant, replicates the experimentally observed vasodynamics more accurately. Therefore, the 5-sec time constant is a reasonable estimate for the NO/cGMP concentration exponential decay.

Our systems biology approach demonstrated that the potentiated myogenic response is primarily responsible for the biphasic vascular reactivity observed in neuronally-evoked vasodynamics, characterized by an initial vasodilation followed by rapid constriction, often leading to a post-stimulus undershoot. However, the constriction phase is blunted following the administration of the NPY receptor antagonist, BIBP ([125]), suggesting a potential interaction between NPY-positive inhibitory neurons and vascular SMCs. This interaction would facilitate inhibitory response within the CBF system (myogenic response), effectively counterbalancing initial blood flow increases triggered by excitatory responses. By comparing the vasodynamics under various SMC $\tau_{mct}$ values (Fig 10A) with those observed after BIBP administration (Fig 10C), where neither the attenuation of initial vasodilation nor the post-stimulus undershoot is present, we can infer that the NPY receptor antagonist significantly slows the myogenic response. In our model, to replicate vasodynamics under BIBP application, the SMC $\tau_{mct}$ would have to increase by a factor of 40–80 or more compared to $\tau_{mct}=5$ sec. Therefore, the vasodynamics observed with BIBP likely represent pure NO-induced vasodilation, which follows the decay of NO/cGMP concentration. This approximately 16–20 sec constriction phase suggests that the NO/cGMP degradation time constant is around 4–5 sec.

In our analysis of vasodynamics in response to neuronal impulse input, we assumed that the brief duration of neuronal activity was insufficient to induce SMC hyperpolarization, either through neuronally released potassium acting on the endothelial network or by direct neuronal activation of SMC glutamate receptors colocalized with BK channels (Section 1). However, if either pathway is activated, the resultant vasodynamics would not change significantly. Fig 10D shows the vascular dynamics where the SMC membrane potential is immediately hyperpolarized due to neuronally mediated increases in EC [*K*^+^]_ex_ and the activation of SMC BK channels for a brief period (1.5 sec). This rapid hyperpolarization of SMCs caused an initial decrease in SMC Ca^2+^ levels, followed by a myogenic response-dependent elevation in SMC Ca^2+^, resulting in a biphasic Ca^2+^ pattern—an initial decline followed by a subsequent increase. Such biphasic Ca^2+^ dynamics have been reported in short-duration (<4–5 s) stimulus-evoked vascular responses [130,131]. In the 1.5 s neuronal activity scenario (Fig 10D), the initial neurogenic network hyperpolarization and associated SMC Ca^2+^ decrease are smaller than the subsequent myogenic post-stimulus depolarization and Ca^2+^ increase (also evident in the Supplementary Material of [130] (Fig 4 and videos)). For slightly longer duration of neuronal activity (3–5 s), the neurogenic hyperpolarization and the subsequent myogenic depolarization become comparable (Fig 3 of [131]). Both our simulations and in-vivo data consistently show post-stimulus uncoupling between Ca^2+^ and vascular tone: significant increases in Ca^2+^ occur after the stimulus, while only marginal or no post-stimulus constriction is observed. This provides validation for our assumption that NO-mediated PKG→MLCP activation reduces Ca^2+^–tone coupling during the phases of FH.

Next, we analyzed vasodynamics in response to neuronal impulse input, assuming that there is ongoing vasomotion in the arteriolar segments. As previously mentioned, the cellular-level PA model oscillates for two to three minutes after the simulation onset, until oscillations are fully damped (Fig 10E, top panel). Here, we assumed that neuronal input was applied to the PA model at four distinct points in time during oscillations including the: oscillation trough, mid-dilation, peak, and mid-constriction. As shown in Fig 10E, the ongoing oscillatory myogenic response influences both the magnitude of the initial dilation and the post-stimulus undershoot of vasodynamics depending on the timing of the neurogenic input to the PA. Furthermore, since NO suppresses the myogenic response for 10–20 sec post-stimulation due to its slow degradation rate, the ongoing vasomotion is damped after the stimulus. This occurs because the vessel is less constricted following the initial stimulus-evoked vasodilation, and the reduced constriction leads to a weaker delayed inhibition of the myogenic response during the dilation phase. As a result, oscillations remain damped and do not recover in this simulation, since we only modeled one PA with no synchronization in the cerebrovascular model. In reality, synchronization in the vascular network may help the oscillation recover post-stimulation, albeit with a new phase compared to the pre-stimulation oscillation, and this new phase is determined by the timing of the stimulus relative to the ongoing oscillation (Fig 10E).

#### Modeling arteriolar vasodynamics in response to neuronal step activity.

**Fig 11:**
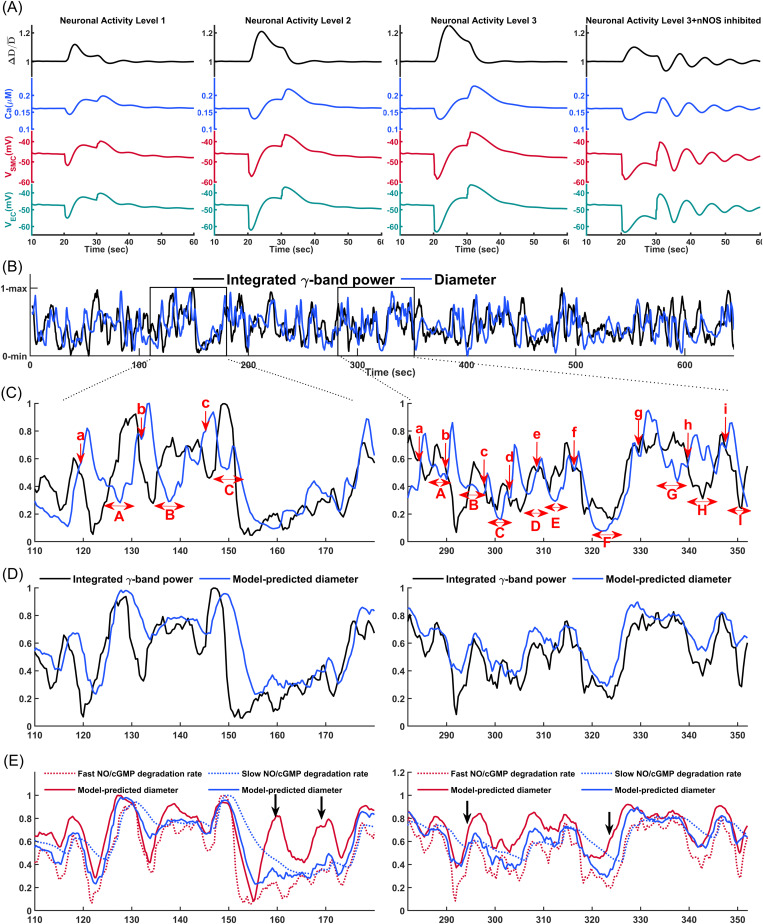
(A) Neurogenic step response of the CBF regulatory system. Arteriolar vasodynamics, vascular cells membrane potential, and SMC [Ca^2+^] are plotted for three hypothetical neuronal activity levels, increasing from level 1 to level 3. The corresponding hypothetical level of neuronal mediators are as follows: EC external potassium concentration changes from 3 mM at the baseline to 4.5, 6, and 7.5 mM; Glutamate concentration changes from 0 at baseline to 0.4, 0.8, and 1.2 AU; NO levels change from 0 at baseline to 2.5, 5, and 7.5 AU. In the right panel, all mediators corresponding to level 3 neuronal activity were applied except NO production. **(B–E)** Role of astrocytes in modulating arteriolar vasodynamics: **(B)** In-vivo recorded integrated gamma-band power and concurrent vasodynamics of an awake mouse as reported by Mateo et al. [26]. **(C)** Magnified view of two range-normalized epochs of in-vivo signals. Single-sided arrows and lowercase letters indicate timestamps for the initiation of astrocytic responses triggered by vessel constriction. Double-sided arrows and capital letters indicate time intervals for delayed potentiated myogenic responses in reaction to astrocytic responses. **(D)** Predicted vasodynamics by the proposed NVC model. **(E)** Predicted vasodynamics by the NVC model under both slow and fast degradation rates of NO/cGMP.

Here, we analyze vasodynamic output in response to a neuronal step input, assuming a relatively stable overall neural activity and minimal neuronal feedback inhibition during sustained stimulation. If the stimulation persists for an extended duration, the neuronally mediated SMC hyperpolarization, in addition to the dampening effect of NO on the myogenic response, remains effective for the entire period of neuronal activity. Fig 11A shows key PA model variables in response to a 10 sec sustained neurogenic input under three different neuronal activity levels with $\tau_{mct}=5$ sec. We assumed that the hypothetical values of all neuronally released mediators, including NO, glutamate, and discharged potassium, increase linearly from level 1 to level 3. As shown, the SMC membrane potential initially hyperpolarizes in proportion to the level of neuronally released SMC hyperpolarization mediators. Simultaneously, NO-induced dilation significantly potentiates the myogenic response, which counteracts the neuronally induced SMC hyperpolarization, causing the SMC [Ca^2+^] to revert toward its pre-stimulation level or even higher after several seconds, until the equilibrium between neurogenic hyperpolarization and myogenic depolarization is established.

In sustained FH in awake mice, such a pronounced SMC [Ca^2+^] undershoot in the first 5 s of stimulation is evident in experimental data [12,132]. Notably, as shown in Fig 1 of [12], during the initial 5 s of a 30 s sensory stimulation, while ∼60% of the initial neurogenic decrease in SMC [Ca^2+^] was reverted by the myogenic response, only ∼20% of the vasodynamic response was reverted. This observation further supports our assumption that NO-mediated PKG→MLCP activation reduces Ca^2+^–tone coupling during the phases of FH. In both [12,132], after myogenic depolarization and constriction within the first 5 s, a secondary phase of SMC [Ca^2+^] reduction is observed during the remaining stimulation interval, which has been attributed to astrocytic responses and is discussed in the next section.

The myogenic response should get more potentiated in neuronal activity level 3 compared to level 2, as the dose-dependent NO-induced vasodilation in level 3 is more pronounced. Consequently, we would expect a larger myogenic response-induced Ca^2+^ elevation in level 3 compared to level 2, although both reach the same [Ca^2+^] and SMC membrane potential by the end of the stimulation. This occurs because the more pronounced potentiated myogenic response in level 3 is counteracted by a stronger neuronally induced SMC hyperpolarization (inhibition of the myogenic response). As a result, the steady-state myogenic tone at the end of stimulation in both levels 2 and 3 are approximately equal. This dynamic is evident when comparing the level of SMC membrane potential depolarization immediately after the stimulus ends. Upon the sudden cessation of neuronal inputs, the SMC depolarization in level 3 is more than level 2, indicating that neuronally mediated inhibition of the myogenic response during the stimulation period was larger in level 3 than level 2.

Next, we analyzed the vasodynamics in neuronal activity level 3, assuming that nNOS is inhibited and NO is not produced during the stimulation period (Fig 11A, right panel). As shown, the neuronally mediated SMC hyperpolarization induced initial dilation which was followed by the myogenic response potentiation and a constriction phase. These simulation results demonstrate that delayed myogenic response potentiation is not solely attributed to NO-induced dilation, and any form of sudden constriction or dilation is followed by the subsequent dilation or constriction, respectively. For example, ATP puffing by micropipette close to vessels in-vivo induced dilation followed by constriction, and this constriction was abolished by P2 receptor antagonists [133], GPCRs that are largely associated with the myogenic tone regulation in the cerebral microvasculature [134].

Post-stimulus (Fig 11A, right panel), with the cessation of neuronal activity, the SMC depolarizes, and the vessel immediately constricts since there is no NO to desensitize the coupling between changes in Ca^2+^ and the constriction force of SMCs. Subsequently, this immediate constriction is followed by a dilation phase caused by the delayed myogenic response, which is then followed by another constriction phase. Similar dynamics were observed in Fig 1 of [135], where application of a NOS inhibitor produced an abrupt post-stimulus constriction followed by oscillatory responses. However, that study examined short (2 s) FH dynamics, in which neurogenic hyperpolarization can evoke the initial dilation and the myogenic response is not fast enough to counteract it, leading to the conclusion that NO is not responsible for the initial sensory-evoked hyperemia. Our simulations highlight that NO plays a critical role in both sustained (>2 s) and instantaneous (<2 s) FH dynamics. In sustained FH, NO dampens the myogenic response to promote dilation and prevents it from being reversed by subsequent potentiated myogenic activity. In instantaneous FH, where neuronally released hyperpolarizing factors are not sustained long enough to fully hyperpolarize the network and drive a pronounced Ca^2+^ reduction in SMCs, NO again serves as the primary vasodilator.

#### Astrocytic Regulation of Arteriolar Vasodynamics

Ultra-slow fluctuations in neuronal signaling, occurring as an envelope over γ-band activity, entrain vasomotion [26]. Therefore, we aimed to test whether modulating arteriolar myogenic response with the envelope of γ-band activity would result in model-predicted vasodynamics that align with experimental observations. Fig 11B shows an example of recorded integrated gamma-band power over 0.4 sec intervals (the envelope of γ-band activity) and concurrent arteriolar vasodynamic measurements are as reported in Mateo et al.’s study. For this test, we used the simplified macro-scale arteriolar segment model, where vessel diameter was determined by delayed myogenic response in coupled segments and passive distension, which resulted in vasomotion in resting state. To include neuronal modulation in this model, we needed to determine the time lag between changes in neuronal activity and the corresponding changes in the myogenic response, whether through inhibition or dampening. Vasodynamics lag behind the envelope of γ-band activity by approximately 1.9 sec ([26]). Therefore, we can modify Eq. 3 as follows:


$$D_{\text{MR}}(AS,i)=-45-V_{\text{final}}(AS,i)-GB(AS,i-\lfloor \frac{1.9\text{ sec}}{dt}\rfloor )$$ (6)

Here, $GB(AS,i-\lfloor \frac{1.9\text{ sec}}{dt}\rfloor )$ represents a factor of the range-normalized γ-band signal, where the real-time myogenic response is inhibited by the γ-band signal from 1.9 sec earlier. We showed that the myogenic response is damped by NO produced during neuronal activity, and that NO/cGMP degradation occurs gradually. Therefore, we initially smoothed the decreasing trend of the range-normalized γ-band signal and then used this smoothed signal to dampen the myogenic response. Fig 11E (blue dashed line) shows the smoothed range-normalized γ-band signal, while Fig 11E (red dashed line) depicts the non-smoothed version. Thus, the real-time vessel diameter can be updated as:


$$D(AS,i)=D_{\text{PD}}(AS,i)+D_{\text{MR}}(AS,i)\cdot \left(1-GB_{S}\left(1,i-\lfloor \frac{1.9\text{ sec}}{dt}\rfloor \right)\right)$$ (7)

The term $\left(1-GB_{S}\left(1,i-\lfloor \frac{1.9\text{ sec}}{dt}\rfloor \right)\right)$ models the dampening effect of neuronally-produced NO on the myogenic response by the smoothed and delayed γ-band signal.

Fig 11C displays a magnified view of two range-normalized epochs of in-vivo signals, and Fig 11D shows the range-normalized predicted vasodynamic by our macro-scale NVC model, plotted alongside the in-vivo γ-band signal. Comparing the model-predicted vasodynamic with the in-vivo vasodynamic shows that, in some cases, the model output shows good similarity to the in-vivo signal. For instance, in epoch 1, between the 145–170 sec interval, the smoothed decay of NO-induced dampening of the myogenic response prevents the immediate resumption of oscillatory myogenic activity after a sudden decrease in neuronal activity. As shown in the red curves in Fig 11E, if the NO/cGMP concentration closely follow the γ-band signal without smoothing (red dashed curve), the rapid decline in diameter would trigger a pronounced delayed myogenic response, leading to a subsequent dilation and the onset of oscillations. Therefore, the slow decay of NO-induced dampening of myogenic response prevents the emergence of myogenic oscillations in arteriolar vasodynamics in awake mice and helps vascular dynamics more closely follow neuronal demand, thereby stabilizing FH. Supporting this, injection of an NO inhibitor reduced the correlation between hemodynamic and LFP signals [136] and increased myogenic oscillations (vasomotion) in mice [135].

Even with the above refinements, significant discrepancies exist between the vasodynamics predicted by the NVC model and the in-vivo vasodynamics, which are associated with the astrocytic response in the CBF regulatory system. For example, take the timestamp ‘b’ in epoch 1. At this point, our model predicted that, due to the decline in the γ-band signal, the vasodynamics would follow a delayed downward trend. However, during the initial moments of this decline, we observed a sudden and unexpected rise. This unexpected deterministic feature is not exclusive to this timestamp; similar events could be seen at all timestamps marked by red single-sided arrows and lowercase letters. For instance, at timestamps ‘a’ and ‘c’ in epoch 1, similar unexpected events occur, and in epoch 2, we observed at least nine similar events within the 70 sec magnified view.

We attributed this deterministic feature of vasodynamics to astrocytes activities based on studies investigating astrocytes contributions to FH. A key study demonstrating that astrocytes possess mechanosensitive TRPV4 channels, which enable them to detect mechanical strain caused by changes in vessel diameter [137]. If, due to ongoing neuronal and synaptic activities, astrocytic endfeet TRPV4 channels are preconditioned with their endogenous ligands, such as epoxyeicosatrienoic acids (EET) [12,138], and if IP3 receptors on the endoplasmic reticulum within astrocytic endfeet are primed for a Ca^2+^ wave [139], paired with astrocytes depolarization caused by potassium removal from brain tissue, then TRPV4-evoked Ca^2+^ signaling in astrocyte endfeet could activate nearby BK channels. This would trigger the release of retained potassium into the surrounding perivascular space (PVS), amplifying the conductance of SMC Kir channels and resulting in SMC hyperpolarization (Fig 4A). Thus, if astrocyte endfeet are primed for significant Ca^2+^ signaling and subsequently detect vessel constriction during FH, they can actively induce arteriolar vasodilation. This mechanism aligns with findings from multiple studies, where Ca^2+^ signaling in astrocyte endfeet was observed post-stimulation in short-duration FH experiments, specifically when PAs began to constrict [140–144].

At timestamp ‘b’ in epoch 1, after astrocyte endfeet detected vessel constriction and released a large amount of potassium near the SMC Kir channels, the vessel dilated. Following this immediate rise in vessel diameter, the delayed myogenic response induced a subsequent constriction phase. Although our model predicted that vasodynamics should remain relatively unchanged during time interval ’B’, the delayed potentiated myogenic response triggered by astrocytic activity at timestamp ‘b’ induced a constriction phase, despite neuronal activity remaining relatively high during the ‘B’ interval. Similarly, during time interval ‘C’ in epoch 1, while our proposed NVC model predicted that vasodynamics would follow the rising trend of the γ-band signal with a delay, the delayed potentiated myogenic response triggered by astrocytic inhibition of the myogenic response at timestamp ‘c’ had a significant impact on vasodynamics. Once this delayed potentiated myogenic response passed, the increased γ-band signal caused vessel dilation, but this dilation was quickly terminated due to the subsequent decline in the γ-band signal. At timestamp ‘f’ in epoch 2, while our NVC model predicted constriction, astrocytic inhibition of the myogenic response caused the vessel to dilate. However, neuronal activity also showed an immediate decline during this time. As a result, during time interval ‘F,’ both the delayed potentiated myogenic response triggered by astrocytic activity at timestamp ‘f’ and the small neuronal inhibition of the myogenic response significantly constricted the vessels. Even the subsequent increase in the γ-band signal could not easily elevate the vessel diameter during this interval, giving the impression that the γ-band signal would lag behind the vasodynamics. However, this was not the case, since the delayed potentiated myogenic response triggered by astrocytic activity drove this unexpected vasodynamics.

Other indicated timestamps for the initiation of astrocytic responses and the corresponding time intervals for delayed potentiated myogenic responses in reaction to these astrocytic signals are well-justified with the proposed NGVC model. As demonstrated, astrocytes periodically inhibit the myogenic response, particularly in response to its potentiation, while neurons continuously send signals to modulate the myogenic response in vessels. Study of these interactions provide a clearer understanding of how astrocytes introduce additional low-frequency components to vasodynamics [137]. Vasodynamics generally should have stochastic low-frequency components from the envelope of the γ-band signal, as the myogenic response is continuously inhibited or dampened by this signal. Additionally, the non-singular frequency of vasomotion, which is more pronounced during periods of low and steady γ-band signal amplitude, also emerges in vasodynamics. Our findings show that astrocytic activity further modulates arteriolar vasodynamics, thereby adding more low-frequency components to the vasodynamics.

## Discussion

### Significance and summary

In this study, we introduced a novel framework for mouse cerebral arteriolar vasodynamics, incorporating interactions among passive, myogenic, neurogenic, and astrocytic responses of the CBF regulatory system. Under resting conditions, passive distension and delayed myogenic responses generate vasodynamic oscillations; however, during dynamic brain activity, neurogenic and astrocytic inputs modulate the myogenic response, disrupt oscillations, and introduce vasodynamic/hemodynamic fluctuations that are approximately 60% correlated with fluctuation in neuronal activity [26,145]. We identified three state-dependent elements in the modulation of the myogenic response that explain the vasodynamic fluctuations not directly correlated with neuronal activity (Fig 11C):

1. The modulation of the myogenic response by neuronal and astrocytic inputs is state-dependent, with the degree of modulation determined by the vascular WS seconds before the input is received.
2. The dynamic of myogenic response is state-dependent and governed by the balance between dampening modulators (e.g., NO/cGMP/PKG/MLCP activation) and facilitatory modulators (e.g., NPY receptor activation). When this balance shifts toward a facilitated myogenic response, sudden vasodynamic changes would induce oscillatory vasodynamics that may not be directly correlated with the ongoing neuronal activity.
3. The modulation of the myogenic response by astrocytes is state-dependent and is periodically triggered during the early phase of myogenic potentiation, when arteriolar constriction is sensed via physical interactions between arterioles and astrocytic endfeet.

These interactions define a state-dependent system, where vasodynamic and hemodynamic outputs depend not only on current inputs but also on the system’s internal state, shaped by prior activity. In this study, we quantified the first two state-dependent myogenic response modulations and identified the third. Future research can further refine this quantitative framework, and by training it with large multimodal datasets of concurrent brain electrical activity and hemodynamic/vasodynamic signals, lay the foundation for developing a bidirectional predictive model of NGVC. This bidirectional model, which allows for more accurate prediction of vasodynamic or hemodynamic changes based on fluctuations in neural activity and vice versa, has potential applications in early detection of neurovascular diseases [146,147], implementation of hemodynamic brain-machine interfaces (BMIs) [148], neuroprosthetics [149], functional connectivity studies [150,151], cognitive neuroscience [152], and pharmacological research [112,153].

### Novel methodological approaches

In this study, we developed both micro-scale (cellular-level) and macro-scale models for arteriolar segments embedded within a coarsely segmented circulatory model of the cerebral vasculature and used that to simulate transient hemodynamic-vasodynamic interactions. Each segment was modeled by transfer functions that relate a wide range of hemodynamic forces—such as WS and WSS—to passive distension and intercellular interactions that adjust the muscular force. The primary goal of introducing these transfer functions was to implement a justifiable autoregulatory mechanism in the developed cerebrovascular model. To add further physiological details, we incorporated appropriate adjustments to the mechanoreactivity of arterioles (by $\tau_{mct}$, and delays) to represent the range of potential myogenic responses to real-time hemodynamic forces. Ultimately, the interplay between the passive distension and active constriction determined the diameter of the arteriolar segment.

### Vasomotion model

Through computational analysis, we showed that under resting conditions, arteriolar segments cannot maintain a stable balance between passive distension and active constriction, leading to low-frequency, high-amplitude pulsatile dynamics. These spontaneous oscillations serve as key regulatory mechanisms in cerebrovascular physiology, including enhancing tissue perfusion [154,155], facilitating glymphatic clearance [31,32], and driving neurovascular synchronization [156]. Therefore, studying the primary signaling mechanisms and the physiological conditions necessary for generating these high-amplitude oscillations is important.

Our proposed vasomotion model differs mechanistically from previously suggested models that rely solely on oscillations generated intrinsically in SMCs via membrane potential or cytosolic calcium oscillators [40,41]. We showed that while the electrophysiological properties of vascular cells, such as large RMR in aSMC-aEC layers are crucial for the vessel’s excitability to mechanical inputs, the mechanical properties of vascular segments—such as distensibility, mechanosensitivity, and mechanotransduction dynamics—also play indispensable roles in generating oscillations. In our model, vascular distensibility acts as the amplifier of wall-stress deviations that sustain oscillations. Additionally, a highly sensitive mechanosensory subsystem in vessels, capable of translating subtle hemodynamic changes into SMC intracellular signaling, is essential for the proper functioning of the delayed negative feedback in this oscillator. This sensitivity diminishes with increased vascular wall thickness, a common pathological feature in conditions such as hypertension [157]. Furthermore, a mechanotransduction dynamics that is neither overly rapid nor excessively slow yields the highest amplitude of pulsatile dynamics (Fig 6B). In-vivo data suggest that mechanotransduction is slowed in aged mice compared to younger animals [131]. Incorporating these detailed functional and morphological characteristics of cerebral arterioles into the vasomotion model establishes a quantitative framework for investigating key factors contributing to vasomotion attenuation in pathological conditions [41].

Our computational results demonstrate that delayed myogenic feedback coupled with passive distension is sufficient to produce sustained oscillations with frequencies and patterns consistent with in-vivo vasomotion observations. However, these results do not constitute proof that this is the exclusive mechanism initiating or maintaining vasomotion in-vivo. Complementary pathways, such as store-operated calcium signaling or neurogenic/astrocytic modulation, may also contribute to vasomotion initiation and amplification in the cerebral vasculature. Moreover, our model assumes deterministic temporal dynamics in the closed WS → V${\;}_{m}\to {\text{Ca}}^{2+}\to$tone (diameter/WS) loop, which produces single-frequency sustained oscillations. However, these in-vivo temporal dynamic features (e.g., delays and rise and fall dynamics of each component of the loop) are stochastic in nature and are continuously modulated by various factors, likely preventing the emergence of sustained single-frequency oscillations and instead producing irregular cycles with multiple frequency components around the fundamental frequency [158]. Future models incorporating variable temporal dynamics into the components of this feedback loop, which would disrupt phase coherence between coupled elements, could better capture the complex multi-frequency vasomotion patterns commonly observed in-vivo [119].

### NVC model

In our proposed NVC model, we categorized the vasodilatory effects of neuronal activity on arterioles into two dominant groups: juxtacrine inhibition of the myogenic response by inducing hyperpolarization in SMCs and ECs, and paracrine dampening of the myogenic response via NO production by nNOS-expressing interneurons. We demonstrated that each dampening or inhibition of the myogenic response (excitatory response) is followed by a potentiation (inhibitory response), facilitated by the activity of NPY-expressing interneurons. This computational model could be further refined by elucidating and incorporating the primary mechanisms and relative contributions of other neuronal cell types involved in the CBF regulatory system [159].

Previously proposed NVC models assumed that NPY acts as a direct vasoconstrictor [19,125,160]. In the model we proposed, NPY is the facilitator of the myogenic response instead of acting solely as a direct vasoconstrictor. Our rationale was based on the comparisons between our simulation results and reported in-vivo observations. In awake mice, arteriolar vasodynamics in response to instantaneous neuronal activity has a post-stimulus undershoot occurring 6–7 sec after the vasodilation onset, followed by a secondary dilation of smaller amplitude in 10–15 sec post-vasodilation onset [125], similar to the simulation results under $\tau_{\text{mct}}=5$ sec as shown in Fig 10A. Therefore, it is reasonable to assume that a facilitated myogenic response is the primary driver of both the constriction phase and the secondary dilation. This secondary dilation phase was also observed in our OG stimulation of the somatosensory cortex in ketamine-dexmedetomidine anesthetized mice [8,127]. In α-chloralose anesthetized mice, the constriction phase was smoother, and the post-stimulus undershoot was prolonged, similar to simulated vasodynamics under $\tau_{\text{mct}}=25$ sec. It was reported that the pharmacological blockade of NPY-Y1 receptors significantly smoothed the constriction phase [125]. These comparisons suggest that: 1. neuronally evoked SMC NPY1R activation facilitates the myogenic response and, 2. different anesthetic agents have differentiable effects on the dynamics of the myogenic response.

### GVC model

The astrocytic response in the CBF regulatory system during FH is triggered when astrocyte endfeet sense constriction in arteriolar segments. In-vitro experiments demonstrated that astrocyte endfeet can detect vessel wall strain changes [137], and in-vivo experiments showed that astrocytes contribute to secondary phase of PA vasodilation during sustained FH [12]. Using in-vivo experimental data, our analysis suggests that during sustained FH, astrocytes detect constriction caused by the delayed potentiation of the myogenic response following initial NVC-mediated vasodilation. Astrocytes induce additional vasodilation, which is subsequently followed by another phase of delayed myogenic response potentiation and arteriolar constriction. This constriction is again detected by astrocyte endfeet, triggering another vasodilation. This cycle may occur two to three times during a 30 sec sustained sensory stimulation in awake mice. Notably, in-vivo signals clearly illustrate this stepwise vasodynamic behavior during sustained FH [12].

Despite the frequent astrocytic responses observed in the dynamically functioning mouse brain (Fig 11C), we cannot fully attribute the astrocyte-dependent vasodilatory response to a feedforward, non-metabolic pathway. In 30 sec sustained FH experimental data, astrocyte endfeet Ca^2+^ signaling consistently occurs during the initial constriction phase following the initial NVC-mediated vasodilation, without exception [12]. In some published datasets from this same study, subsequent Ca^2+^ signaling events in astrocyte endfeet are not observed. Since neuronal activity did not show a significant decrease over time during their 30 sec FH experiments, it seems likely that the attenuation of astrocytic responses in the later phase of stimulation involved metabolic-related factors. After the first or second step-wise astrocyte-mediated vasodilation in PAs, and the consequent delivery of more blood to the activated region, the astrocyte endfeet may become less preconditioned for substantial Ca^2+^ signaling. These observations suggest that astrocytes likely play a pivotal role in a metabolic feedback mechanism within the brain parenchyma [161]. Elucidating the signaling mediators involved in the astrocytic response in CBF regulation might offer a clearer understanding of their contributions to feedforward and feedback pathways.

We could not find the deterministic feature of astrocytic response in optical or magnetic in-vivo signals acquired from anesthetized mouse brains, including in our experiments, likely because anesthesia disrupts astrocyte calcium signaling [162,163]. Different anesthetics used in various experimental settings may have different effects on the function of astrocytes [164]. For example, in concurrent recording of BOLD fMRI and Ca^2+^ signaling in anesthetized rat brain during 20 sec sensory stimulations, sometimes post-stimulus glia-related positive BOLD signals were observed immediately post-stimulus, accompanied by increased Ca^2+^ signaling in astrocytes during the stimulus [165]. This post-stimulus positive BOLD fMRI may have arisen from arteriolar vasodilation induced by astrocytic potassium release when vessels initially began to constrict post-stimulus. Astrocytes’ ability to detect vasoconstriction and release potassium into the PVS to induce rapid vasodilation could enhance the amplitude of vasomotion and low frequency vasodynamics in the brain. This mechanism would interrupt the constriction phase early, amplifying vasodilation, which is followed by a more pronounced myogenic response potentiation and vasoconstriction. This idea is supported by in-vivo experimental data which showed that inhibiting astrocyte endfeet Ca^2+^ signaling reduces the amplitude of low-frequency vasodynamics in PAs[137].

### Assumptions and simplifications

In this study, several levels of simplifications and assumptions were incorporated to model an abstract version of the CBF regulatory system, allowing us to focus exclusively on the dominant signaling pathways that shape arteriolar vasodynamics. Despite notable differences between PAs and other vascular segments with large contractility such as sphincters and precapillary arterioles [166–170], it seems plausible that sphincters and precapillary arterioles share similar mechanisms with PAs in shaping their vasodynamics. For example, in the case of vasomotion, sphincters and TZ vessels must also oscillate to provide a similar level of pulsatile dynamics across different cortical depths. If vasomotion were restricted to the arteriolar level and absent in sphincters and precapillary arterioles, capillaries in deeper regions would experience pronounced blood flow oscillations, while blood flow in superficial capillaries would have minimal oscillations. This does not align with physiological expectations. If the NGVC dynamics of sphincters and TZ vessels differ significantly from those of PAs, more blood would be directed toward deeper cortical layers during FH, which again would not align with physiological norms. In-vivo recorded vasodynamics in TZ vessels and Ca^2+^ dynamics in the ensheathing pericytes that encase these vessels corroborate that vasomotion, and low-frequency vasodynamics are also present in TZ vessels, but no vasodynamics have been detected in capillaries during normal brain functioning [171]. It would be valuable to know whether sphincters and precapillary arterioles share similar mechanisms with PAs in regulating CBF.

Previous experimental studies observed delayed vasodilation and increases in positive BOLD fMRI signals in the superficial layers of the awake mouse brain following induced instantaneous neuronal activity [125,128,172]. In this study we assumed that there is a cortical depth-dependent time lag in the increase of NO around aSMCs, with superficial aSMCs experiencing this NO increase with a longer delay compared to deeper ones (Fig 9D). The underlying mechanisms behind this phenomenon remain unclear. Given that neuronal activity is known to propagate rapidly across cortical layers, then depth-dependent properties of neurovascular communication become a plausible factor [128]. Further investigation is required to fully elucidate the origins of this delay in superficial layers and to determine whether the CBF regulatory system leverages such depth-dependent kinetics to optimize blood delivery across cortical layers. Furthermore, our model assumes that NO primarily mediates vasodilation through the PKG→MLCP activation pathway affecting calcium sensitization, rather than through direct modulation of SMC ion channels. While our simulations recapitulate in-vivo observations of reduced SMC Ca^2+^-tone coupling during and post-FH, we emphasize that this assumption represents one mechanistic hypothesis requiring further experimental validation.

It remains unclear whether neurons release potassium around ECs in specific vascular zones and whether ECs in regions with recurrent neural engagement adapt their Kir channel expression in response. This “vascular signaling plasticity” [173] would allow the cerebral vasculature to better match the blood supply with ongoing neuronal activity. In our model, we simplified the EC-dependent mechanism of aSMC membrane potential modulation in response to neuronal activity, considering only an increase in extracellular potassium in aECs during periods of elevated neuronal activity. If a substantial number of ECs across different vascular zones within the parenchyma experience this rise in [*K*^+^]_ex_ during sustained neuronal activity, the endothelial layer could theoretically shift to a quantized state of hyperpolarization and maintain this state throughout the period of elevated activity. This shift would result in more pronounced inhibition of the myogenic response by ECs than modeled in this study when examining vasodynamics in response to step neuronal input. The myogenic response-dependent SMC depolarization following initial NVC-mediated vasodilation could also depolarize aECs through gap junctions and induce a prominent initial overshoot in vasodynamics (Fig 11A, right panel). On the other hand, if local endothelial layer membrane potential is strictly regulated by well-connected EC networks responding to localized neuronal activity, this SMC-mediated EC depolarization may not occur.

Our primary focus in this study was on feedforward NGVC signaling pathways, where non-vascular cells, in proportion to their physiological demand, send signals to vascular cells to modulate myogenic tone and induce vasodilation. However, when baseline neuronal activity is low and an abrupt stimulus sharply elevates demand, delays in feedforward NVC (from neuronal signaling to the corresponding vasodilation) can lead to substantial transient oxygen mismatches [174–177]. In such cases, RBCs respond to falling PO_2_ by deforming to facilitate capillary flow, which increases WSS in vessels. Research has demonstrated that mechanosensitive Piezo1 channels in ECs can be activated by this increased WSS, triggering eNOS-mediated NO production and vasodilation [178,179], which augments nNOS-derived NO in cerebral vasculature to facilitate FH. Notably, FH data from short-duration stimuli showed a fast, NO-dependent vasodilatory response that was completely abolished in eNOS knockout mice [9]. Our modeling focused on dynamically functioning brains, assuming NGVC-driven vascular responses efficiently deliver oxygen without invoking RBC deformation and therefore attributing NO production primarily to nNOS. These metabolic and mechanosensitive pathways were not included in the present model, but they serve as important regulatory mechanisms that can profoundly influence cerebrovascular dynamics and can be explored in future works.

## Methods and materials

### Ethics statement

All animal procedures were approved by the William S. Middleton Memorial Veterans Hospital Institutional Animal Care and Use Committee (IACUC), protocol number RA0010.

### Hemodynamic-vasodynamic simulations in the cellular-level PA model

To build a computational model for arteriolar segments, the first step is to model how mechanosensitive ion channels in vascular cells are modulated by hemodynamic forces. These forces regulate ion flux through mechanosensitive channels, which, in combination with ion flux through non-mechanosensitive channels, shape the electrochemical gradients and resting conditions of the cell. To define the segment-specific dynamic range of hemodynamic forces, we extracted *WS*_max_ and *WSS*_max_ for each arteriolar segment. This range was determined under the assumption of static autoregulation in the cerebral vasculature, where each segment operates under specific WS-MT and WSS-MT transfer functions, enabling autonomous adjustment of vessel segment contraction state based on hemodynamic inputs. Given that WS-MT has more control over MT adjustments than WSS-MT within the physiological range of hemodynamic forces [46], we particularly aimed to build a more detailed model of aSMCs than aECs in this study.

The SMC electrophysiology was modeled using a system of ODEs, primarily adapted from Karlin’s study [85], with adjustments for PA SMCs. The T-type VOCC/BK/RyRs microdomain, which minimally affects PA SMC negative feedback in MT regulation, was not incorporated in the model, while a BK-Glu microdomain was included as part of the NGVC mechanisms. This microdomain incorporates NMDA receptors that, upon activation by neuron-released glutamate, facilitate Ca^2+^ efflux to activate BK channels. The inclusion of such microdomains reflects the requirement for localized [Ca^2+^] to exceed the cytoplasmic [Ca^2+^] in SMCs to activate Ca^2+^-sensitive channels. Additionally, a wall stress-transducing microdomain (WS-TM) was incorporated to account for the Ca^2+^-dependent activation of key channels involved in MT regulation, such as TMEM16A and TRPM4. Without the WS-TM, a positive feedback loop would occur, where depolarization triggers further Ca^2+^ influx via VOCCs, continuously activating these Ca^2+^-sensitive depolarizing channels. Thus, incorporating a macrodomain where Ca^2+^ modulation is controlled exclusively by non-voltage-gated channels, such as mechanosensitive TRPC6, was essential.

For the mechanosensory subsystem of the SMC model, given that the signaling cascades associated with this subsystem are not fully understood, we applied simplifications that may not be entirely physiologically accurate. We assumed that WS linearly influences phosphatidylinositol 4,5-bisphosphate (PIP2) concentration through the mechanoactivation of G-protein-coupled receptors (GPCRs) [180]. Specifically, an increase in WS enhances the conversion of PIP2 into IP3 and DAG. This relationship was modeled as a quasi-linear sigmoidal relationship between increased WS and reduced PIP2, leading to both a decrease in the probability of Kir channel activation [180] and, in parallel, an increase in DAG and IP3 concentrations (Fig 3A). The elevated DAG and IP3 subsequently raise the open probability of TRPC6 channels [51] and IP3 receptors (IP3Rs) within the WS-TM, respectively. The activation of TRPM4 channels in the WS-TM, via transient Ca^2+^ release from IP3Rs, generates transient inward cation currents (TICCs), where the frequency of TICCs is influenced by the concentrations of Ca^2+^ and IP3 within the WS-TM. In our model, this cascade was simplified by focusing on steady-state conditions. This steady-state approach streamlines the system of ODEs by eliminating the need to model numerous microdomains where transient currents occur. Instead, the cumulative effects of these transient currents are captured in the steady-state activation levels of channel pools, which reduce the computational demands for simulations. With this simplification, we assumed that IP3 concentration directly increases the open probability of TRPM4 channels rather than acting indirectly through IP3Rs [81]. As a result, the activity of TRPM4 channels is regulated by WS/[DAG]/TRPC6/[Ca^2+^]_TM_ and WS/[IP3]_TM_. Similarly, the mean ion flux through TMEM16A channels, calcium-activated chloride channels assumed to be localized in the WS-TM, is regulated by WS/[DAG]/TRPC6-[Ca^2+^]_TM_ and the SMC membrane potential.

Our model incorporates relatively high Kir channel densities in aSMCs with WS-dependent modulation of their activity. Sancho et al. provided convincing experimental evidence supporting the mechanosensitivity of these channels in pial SMCs [55]. However, the assumed maximal Kir channel conductance in our aSMC model is higher than what their patch-clamp data suggest. Our primary experimental constraint for determining this conductance was the need to replicate the myogenic tone profile observed in pressure myography of endothelium-denuded vessels ex-vivo. Specifically, our model needed to reproduce the initial dilation at low pressures before reaching maximum dilation at the lower limit of the autoregulation range—a behavior that required high Kir channel conductance and was completely blunted under Ba^2+^ application ex-vivo [55]. Therefore, our assumed aSMC Kir conductance values should be interpreted as effective parameters that enable the model to capture key features of myogenic behavior (Fig 3B), and also respond appropriately to elevated extracellular potassium (Fig 4A). To the best of our knowledge, no prior ex-vivo studies have examined potassium challenges at various pressure levels in endothelium-denuded PAs, and most potassium challenge experiments have been conducted on intact vessels at constant pressure. Due to electrical coupling between aECs and aSMCs, and since Kir channels act as amplifiers of hyperpolarization due to their inward rectification mechanism, any hyperpolarization initiated in aSMCs in intact vessels (e.g., by elevated [*K*^+^]_ex_) can be further amplified by Kir channels in aECs, making it difficult to determine cell-specific Kir channel contributions from intact arteriolar data. Potassium challenges in endothelium-denuded arterioles under various pressure levels could be explored in future ex-vivo studies to better characterize aSMC Kir channel open probability, conductance, and rectification profiles.

Similarly, our model incorporates relatively high Kir channel densities in aECs with experimentally supported WSS-dependent modulation of their activity [55] to avoid contradicting myogenic autoregulation (Fig 5A and 5B). Patch-clamp experiments on isolated ECs are typically measured under no-flow conditions, which may not reflect maximal Kir activity in intact, perfused vessels. Our central motivation for assuming high aEC Kir channel densities was to enable local conversion of elevated extracellular potassium into EC hyperpolarization at the PA level as a mechanism for NVC (Fig 5E). However, we acknowledge that this assumption represents one possible mechanistic scenario for NVC at the PA level, but not the only possibility. In reality, the primary site of endothelial K^+^-mediated hyperpolarization may occur at downstream capillaries, with PA ECs serving predominantly as conduits that amplify and transmit hyperpolarization signals rather than as sites of primary K^+^ sensing and conversion [66]. Such a scenario would not necessarily require high Kir channel densities in PA endothelium and warrants further experimental investigation.

Considering these uncertainties and simplifications in the electrophysiology of the developed PA model, we present qualitative comparisons with voltage-clamp experiments (Fig 3D) and pressure myography maneuvers (Fig 3B) to support key features of our developed cellular PA model. However, many simulated values reported in Section 1, such as RMR, RMP, and the degree of vasodilation/constriction during potassium challenges, should be interpreted as exploratory scenarios that enable investigation of putative mechanisms rather than quantitative predictions directly validated by experimental measurements. For detailed equations governing SMC and EC electrophysiology, including those describing channel currents and intracellular ion concentrations, readers are referred to [85] and to our supplementary material (S1 Text).

Upon establishing and validating the electrophysiological model, we integrated it with another critical component of the arteriolar segment model: vessel contraction adjustments by modeling the interplay between passive distension and SMC constriction mechanisms. Importantly, its output (i.e., vessel diameter) serves as the primary input for hemodynamic analysis. To effectively represent the SMC constriction mechanism through the dynamic modulation of MLCK and MLCP activities, we focused on the dominant signaling mechanisms proposed in this study. Specifically, the model links the SMC electrophysiology framework via Ca^2+^-mediated modulation of MLCK, while enabling NO/cGMP/PKG-driven modulation of MLCP activities in response to external stimuli from ECs and nNOS-expressing interneurons. To achieve this, we employed the four-state Michaelis-Menten kinetics model proposed by [98] and adapted by [100]. This model captures transitions between four distinct myosin states: unphosphorylated myosin (M), phosphorylated myosin (Mp), actin-bound unphosphorylated myosin (AM), and actin-bound phosphorylated myosin (AMp). A system of ODEs describes the temporal evolution of these states, with rate constants defining the transitions. The Ca^2+^ dependency of actomyosin interactions, along with the effects of NO/cGMP/PKG signaling, was incorporated by modulating these rate constants. By conserving the total myosin across all states, the model provides a robust framework for simulating SMC constriction dynamics. For further details on the implementation and parameterization of this model, readers are referred to [181], and to our supplementary material (S1 Text).

The PA segmental model was then integrated into the cerebrovascular model. Algorithm 1 outlines the hemodynamic-vasodynamic simulation workflow developed to investigate the kinetics of vasomotion and FH dynamics using this cellular PA model. The hemodynamic analysis was based on the research conducted by Secomb’s [182] and Pries’s [47] groups.

**Algorithm 1 hemodynamic-vasodynamic simulation workflow in cellular-level PA model**

1: Load the cerebrovascular model;

2: Set the ABNP to 60 mmHg and adjust the fixed diameters for all segments to model autoregulation at this ABNP value;

3: Integrate the PA segmental model into each segment of one PA;

4: Adjust the segmental PA model parameters to account for morphological variations across different segments along the PA penetration;

5: **Begin the time-domain simulation**

• Vasomotion Dynamics Analysis (*t* < 150 sec)

- Hemodynamic analysis of the cerebrovascular model; input: real-time diameter, output: WS and WSS for each segment (every 0.2 sec)

- Vasodynamic analysis of the PA computational model; inputs: WS and WSS for each segment, output: updated diameters (every 0.1 msec)

• FH Dynamics Analysis (*t* > 150 sec)

- Hemodynamic analysis of the cerebrovascular model; input: real-time diameter, output: WS and WSS for each segment (every 0.2 sec)

- Vasodynamic analysis of the PA computational model; inputs: WS, WSS, EC [*K*^+^]_ex_, NO, and glutamate; output: updated diameters (every 0.1 ms)

6: **end of simulation**

### Hemodynamic-vasodynamic simulations in the network of macro-scale models of coupled arteriolar segments

The developed simplified macro-scale model of arteriolar segments incorporates the delayed myogenic response, passive distension, and electrical coupling between adjacent segments. Algorithm 2 outlines the simulation workflow. The model begins by setting initial conditions, calculating *WS*_*avg*_ based on IP, D, and constant vessel wall thickness (h). The variables related to the myogenic response, passive distension, and electrical coupling in the model are defined to simulate vasomotion and FH dynamics. In the analysis of vasomotion dynamics, where the myogenic response is not modulated by stochastic γ-band signals, the membrane potential of each arteriolar segment is initially computed solely based on its hemodynamic inputs (*V*_*init*_(*AS*)). To model the resistive-capacitance nature of electrical coupling between segments, a secondary coupling loop is introduced. This loop operates at a finer time step (*dt*_2_ = 0.4 msec) to avoid fast and large influences of adjacent segments on each other. In this loop, the difference in membrane potential between adjacent segments is calculated, and the membrane potential of each segment is iteratively updated. $\Delta V_{\text{coupling}}(AS,j)$ represents the cumulative voltage differences between each segment and its adjacent segments. The final membrane potential at the end of this loop (*V*_*final*_(*AS*, *i*)) is used for subsequent calculations in the main loop. The passive portion of the diameter (*D*_PD_(*AS*,*i*)) is then computed based on the real-time WS and depends on the distensibility parameters and the minimum diameter under no pressure conditions (unpressurized state), which was assumed to be 90% of the maximum active diameter (Fig 3B). The total diameter of each segment is the sum of the MR portion and the passive portion. Finally, if the updated diameter exceeds the maximum allowable diameter change in one main loop iteration, the change is constrained by the maximum allowable dilation ($\Delta D_{{\text{max}}_{\text{dila.}}}(AS)$) or constriction ($\Delta D_{{\text{max}}_{\text{const.}}}(AS)$) rates. During the FH vasodynamic analysis, all steps remain unchanged except for the inhibition of MR by the γ-band signal (*GB*), and the dampening of MR by the smoothed γ-band (*GB*_*S*_), as described in Section 3.

### Animal preparation and in-vivo imaging

We used 6–12 month-old transgenic optogenetic mice (THY:ChR2/H134R-YFP). Mice were anesthetized with isoflurane (1.5%–2.0% in oxygen), and the scalp was retracted to thin the skull over the somatosensory cortex. A 3 mm coverslip was installed approximately 1.5–2 mm lateral and 1.5–2 mm posterior to bregma. The average heart rate during surgery was 450–550 bpm. Afterward, anesthesia was switched to ketamine (25–50 mg kg^-1^ SC) and dexmedetomidine (0.05–0.5 mg kg^-1^ SC) for imaging. Heart rate during imaging was 250–350 bpm, with peripheral capillary oxygen saturation between 95–100%. Body temperature was maintained at 36.5–37.5° C using a heating pad. Cerebral blood flow (CBF) was recorded using laser speckle contrast imaging (LSCI; RFLSI III, RWD Life Sciences).

### Statistical analysis

In Section 3, we quantified the onset time of OG-evoked vasodilations at various cortical depths from Uhlirova’s dataset to assess the speed of vasodilation propagation in response to instantaneous neuronal activity. A sigmoidal function was fitted to the diameter increase data to determine the onset of dilation, identified by the point where the first derivative of the fitted curve crossed a predefined threshold, marking the start of the diameter increase.

**Algorithm 2 hemodynamic-vasodynamic simulation workflow in a network of macro-scale models of coupled arteriolar segments**

1: Load the cerebrovascular model

2: Set the ABNP to 60 mmHg and adjust the initial diameters for all segments to model autoregulation at this ABNP value;

3: Initialize model variables for arteriolar segments (AS); $V_{final}(AS,i=0)=-45mv$, *D*(*AS*,*i* = 0) =*D*(*AS*);

4: Hemodynamic analysis of the cerebrovascular model to calculate *IP*(*AS*,*i* = 0);

5: Calculate the average arteriolar segment WS values (*WS*_*avg*_(*AS*)) based on *IP*(*AS*,*i* = 0), *D*(*AS*,*i* = 0), and the constant $h(AS)=0.1\times D_{{active}_{max}}$, where $D_{{active}_{max}}$ represents the segment diameters at ABNP = 40 mmHg.

6: Define model variables: delayed myogenic response variables: $MR_{D}=5\;sec$, $MR_{F}=19\times 10^{-3}\frac{mv}{mmHg}$; passive distension variables: $D_{WS_{min}}=0.9\times D_{{active}_{max}}$, α=0.5, *K* = 3 × 10^−3^; coupling time constant: *RC* = 1 × 10^−3^ sec; maximum allowable diameter change rate: $DR_{\text{dilation}}=7.5\frac{\%}{sec},DR_{\text{constriction}}=7.5\frac{\%}{sec}$; Neuronal MR inhibition factor: $N_{MR_{Inh}}=10$;

7: **Begin time-domain simulation** (loop variable: i, *dt*_1_=0.2 s)

• Vasomotion Dynamics Analysis

- Hemodynamic analysis of the cerebrovascular model; input: real-time diameter *D*(*AS*, *i* − 1), output: *WS*(*AS*,*i*)

- $V_{init}(AS)=-45+MR_{F}\times (WS(AS,i-\frac{MR_{D}}{dt_{1}})-WS_{avg}(AS))$

- Define $V_{temp}(AS,1)=V_{init}(AS)$ to initialize the coupling loop

- Coupling loop (loop variable: j, *dt*_2_ = 0.1 ms)

* Calculate the sum of voltage differences between each segment and its adjacent segments: $\Delta V_{\text{coupling}}(AS,j)=\sum (V_{\text{temp}}(\text{adjacent},j-1)-V_{\text{temp}}(AS,j-1))$

* Update the membrane potential: $V_{temp}(AS,j)=V_{temp}(AS,j-1)+\frac{1}{RC}\times \Delta V_{\text{coupling}}(AS,j)\times dt_{2}$

- *V*_*final*_(*AS*, *i*) = *V*_*temp*_(*AS*, *end*)

- $D_{\text{PD}}(AS,i)=D_{WS_{min}}\times (1+\alpha \times (1-\exp(-K\times WS(AS,i)))$

- $D_{\text{MR}}(AS,i)=-45-V_{final}(AS,i)$

- $D(AS,i)=D_{PD}(AS,i)+D_{MR}(AS,i)$

- $\Delta D_{{\text{max}}_{\text{dila.}}}(AS)=DR_{\text{dilation}}\times dt_{1}\times D(AS,i-1)$

- $\Delta D_{{\text{max}}_{\text{const.}}}(AS)=DR_{\text{constriction}}\times dt_{1}\times D(AS,i-1)$

- **If** $D(AS,i)-D(AS,i-1)>\Delta D_{{\text{max}}_{\text{dila.}}}(AS)$

- **Then** $D(AS,i)=D(AS,i-1)+\Delta D_{{\text{max}}_{\text{dila.}}}(AS)$

- **If** $D(AS,i)-D(AS,i-1)<-\Delta D_{{\text{max}}_{\text{const.}}}(AS)$

- **Then** $D(AS,i)=D(AS,i-1)-\Delta D_{{\text{max}}_{\text{const.}}}(AS)$

• FH Dynamics Analysis

- ....

- $D_{\text{MR}}(AS,i)=-45-V_{final}(AS,i)-N_{MR_{Inh}}\times GB(AS,i-\lfloor 1.9\text{ sec}/dt_{1}\rfloor )$

- $D(AS,i)=D_{PD}(AS,i)+D_{MR}(AS,i)\times ((1-GB_{S}(1,i-\lfloor 1.9\text{ sec}/dt_{1}\rfloor )))$

- ....

8: **End of simulation**

## Supporting information

S1 Text Mathematical model of the segmented penetrating arteriole. (PDF)
