# Supplementary material for: Systems biology analysis of vasodynamics in mouse cerebral arterioles during resting state and functional hyperemia
Source: PLoS Comput Biol. 2026 Apr 27;22(4):e1013113. doi: 10.1371/journal.pcbi.1013113 (PMC13138759; doi:10.1371/journal.pcbi.1013113)
Supplement: S1 Text — (PDF) [file pcbi.1013113.s001.pdf]

Supplementary Material

Mathematical Model of the Segmented Penetrating Arteriole

Supplementary Material for

Systems biology analysis of vasodynamics in mouse cerebral arterioles during resting state and functional hyperemia

Hadi Esfandi[1,2], Mahshad Javidan[1,2], Eric R. McGregor[2], Rozalyn M. Anderson[2,3], Ramin Pashaie[1*]

1 Electrical Engineering and Computer Science Department, Florida Atlantic University, Boca Raton, FL, USA
2 Department of Medicine, University of Wisconsin–Madison, Madison, WI, USA
3 Geriatric Research, Education, and Clinical Center, William S. Middleton Memorial Veterans Hospital, Madison, WI, USA

* Correspondence: rpashaie@fau.edu

Overview

The segmented penetrating arteriole model presented here was specifically designed for a particular arteriolar segment within our cerebrovascular model, where its dynamic range of hemodynamic forces—such as WS and WSS—was previously characterized under the assumption of static autoregulation. To generalize this model for application to any arteriolar segment within any cerebrovascular model, it is necessary to calibrate key variables, including those related to the dynamic range of hemodynamic forces and the morphological characteristics of the vessel segment (e.g., baseline diameter and relative contractility), to ensure proper functionality. This segmented arteriolar model includes an electrophysiological model (SM 1) and a mechanobiological model (SM 2).

SM 1: Arteriolar Electrophysiological Model

The electrophysiological model of this arteriolar segment, which includes a detailed model of an aSMC electrophysiology, is primarily adapted from Karlin's study[1]. The aSMC is electrically coupled via gap junctions to a simplified electrophysiological model of an aEC. Ion flux across the channel models is quantified in femtomoles per second (fmol/s), and electrical current is expressed in picoamperes (pA).

BK channel

BK channel activity requires calcium concentrations in the micromolar range. To achieve this, specific microdomains are defined where calcium influx accumulates in confined regions, locally reaching the necessary micromolar concentrations. The T-type VOCC/BK/RyRs microdomain, which minimally affects PA SMC negative feedback in MT regulation, was not incorporated in the model, while a Glu-BK microdomain was included as part of the NGVC mechanisms. This Glu-BK microdomain includes NMDA receptors that, upon activation by neuron-released glutamate, trigger Ca^{2+} influx to activate BK channels. The activation dynamics of the BK channel in the SMC are described by the following equation [2]:

$$\frac{dP_{\mathrm{BK}}\left(v, Ca_{\mathrm{BK\text{-}Glu}}\right)}{dt} = \lambda_{\mathrm{BK}} \cdot \left(K_{\mathrm{BKact}}\left(v, Ca_{\mathrm{BK\text{-}Glu}}\right) - P_{\mathrm{BK}}\right) \tag{S1}$$

with the equilibrium state K_{BKact} given by:

$$K_{\mathrm{BKact}}\left(v, Ca_{\mathrm{BK\text{-}Glu}}\right) = \frac{Ca^2_{\mathrm{BK\text{-}Glu}}}{Ca^2_{\mathrm{BK\text{-}Glu}} + \beta_{\mathrm{BK}} \cdot \exp\left(\mathrm{V_{BK}} - \frac{v}{\mathrm{R_{BK}}}\right)}. \tag{S2}$$

Here, the constants λ_{BK}, V_{BK}, and R_{BK} describe the channel activation properties by membrane potential (v) and $Ca_{\mathrm{Glu\text{-}BK}}$. The BK channel flux J_{BK} is calculated using a form of the Goldman-Hodgkin-Katz (GHK) current equation:

$$J_{\mathrm{BK}}(v, Ca_{\mathrm{Glu\text{-}BK}}, K_{\mathrm{in}}, K_{\mathrm{ex}}, WSS) := \begin{cases} 10^9 \cdot \mathrm{BK}_T \cdot \mathrm{perm_{BK}} \cdot P_{\mathrm{BK}}(v, Ca_{\mathrm{BK\text{-}Glu}}) \cdot \mathrm{BK_{WSS\_gain}} \cdot \\ \frac{WSS}{\mathrm{WSS_{max}}} \cdot \left(K_{\mathrm{ex}} - K_{\mathrm{in}}\right), & \text{if } v = 0 \\ 10^9 \cdot \mathrm{BK}_T \cdot \mathrm{perm_{BK}} \cdot P_{\mathrm{BK}}(v, Ca_{\mathrm{BK\text{-}Glu}}) \cdot \mathrm{BK_{WSS\_gain}} \cdot \\ \qquad \frac{WSS}{\mathrm{WSS_{max}}} \cdot z_K \cdot \beta \cdot v \cdot \frac{K_{\mathrm{ex}} - K_{\mathrm{in}} \exp(z_K \cdot \beta \cdot v)}{\exp(z_K \cdot \beta \cdot v) - 1}, & \text{if } v \neq 0 \end{cases} \tag{S3}$$

The BK channel current I_{BK} is then given by:

$$I_{\mathrm{BK}}\left(v, Ca_{\text{Glu-BK}}, K_{\mathrm{in}}, K_{\mathrm{ex}}, WSS\right) := -\frac{z_K \cdot \mathrm{F} \cdot J_{\mathrm{BK}_i}(v, Ca_{\mathrm{m}_i}, K_{\mathrm{in}}, K_{\mathrm{ex}})}{1000} \tag{S4}$$

In our model, the open probability of BK channel (P_{BK}) is also linearly modulated by NO produced by eNOS in response to increased WSS, acting to mitigate MT and prevent excessive WSS. Accordingly, P_{BK} is scaled by $\mathrm{BK}_{\mathrm{WSS\_gain}} \cdot \frac{WSS}{\mathrm{WSS}_{\mathrm{max}}}$ in Eq. S3.

Table S1: BK channel model parameters[2]

**Parameter**	**Value and Unit**
λ_{BK}	45 s^{-1}
β_{BK}	1 $\mu\mathrm{mol}^2$
V_{BK}	-27 mV
R_{BK}	12 mV
BK_T	6.0
$\mathrm{perm}_{\mathrm{BK}}$	$4 \times 10^{-13}\ \mathrm{cm}^3\,\mathrm{s}^{-1}$
$\mathrm{BK}_{\mathrm{WSS\_gain}}$	20

KV channel

$$P_{\mathrm{KV}}(v) := \frac{1}{1 + \exp\left[\frac{-(v - \mathrm{V}_{\mathrm{KV}})}{\xi_{\mathrm{KV}}}\right]} \tag{S5}$$

$$J_{\mathrm{KV}}(v, K_{\mathrm{in}}, K_{\mathrm{ex}}) := \begin{cases} 10^9 \cdot \mathrm{KV}_T \cdot \mathrm{perm}_{\mathrm{KV}} \cdot P_{\mathrm{KV}}(v) \cdot \left(K_{\mathrm{ex}} - K_{\mathrm{in}}\right), & \text{if } v = 0 \\ 10^9 \cdot \mathrm{KV}_T \cdot \mathrm{perm}_{\mathrm{KV}} \cdot P_{\mathrm{KV}}(v) \cdot z_K \cdot \beta \cdot v \cdot \dfrac{K_{\mathrm{ex}} - K_{\mathrm{in}} \exp(z_K \cdot \beta \cdot v)}{\exp(z_K \cdot \beta \cdot v) - 1}, & \text{if } v \neq 0 \end{cases} \tag{S6}$$

$$I_{\mathrm{KV}}\left(v, K_{in}, K_{ex}\right) := \frac{-\mathrm{z}_{\mathrm{K}} \cdot \mathrm{F} \cdot J_{\mathrm{KV}}\left(v, K_{in}, K_{ex}\right)}{1000} \tag{S7}$$

Table S2: $\mathrm{K_V}$ channel model parameters[1]

**Parameter**	**Value and Unit**
V_{Kv}	6 mV
ξ_{Kv}	14 mV
$\mathrm{perm}_{\mathrm{Kv}}$	2.5×10^{-14} cm^3 s^{-1}
Kv_T	1.2×10^3

WS-Dependent Ion Channels (TRPC6, TRPM4, TMEM16A, $\mathbf{Kir_{SMC}}$)

For the mechanotransduction subsystem of the SMC model, we made a simplifying assumption that may not be entirely physiologically accurate. Specifically, we assumed that WS linearly modulates the intracellular concentration of PIP2 through the mechanoactivation of GPCRs [3]. An increase in WS promotes PIP2 hydrolysis, reducing its concentration while elevating the levels of IP3 and DAG. The resulting increase in DAG and IP3 enhances the activity of TRPC6 channels [4] and IP3Rs, which in turn boost the activation of the primary depolarizing channels, TMEM16A and TRPM4. Meanwhile, the reduction in PIP2 lowers the open probability of Kir channels, thereby suppressing the main hyperpolarizing current [3]. The temporal evolution of these biochemical processes was modeled using first-order kinetics with a mechanotransduction time constant (τ_{mct}).

Kir channel[5, 6]

$$\frac{dKir_{\mathrm{OP}}}{dt} = -\frac{1}{\tau_{\mathrm{mct}}}\left(Kir_{\mathrm{OP}} - Kir_{\mathrm{OP\_SS}}\right) \tag{S8}$$

$$Kir_{\mathrm{OP\_SS}}(R,P) = 1 - \frac{1}{1+\exp\left(-\mathrm{k}_{Kir_{OP}} \cdot \left(\frac{R\cdot P}{\mathrm{h}} - \frac{\mathrm{WS_{max}}}{2}\right)\right)} \tag{S9}$$

$$I_{Kir}(R,P,v,K_{ex}) = \frac{\bar{\mathrm{G}}_{Kir}\sqrt{K_{ex}}\,(v-E_K)}{1+\exp\left(\frac{v-V_{0.5_{kir}}}{\mathrm{k_{kir}}}\right)} \cdot Kir_{\mathrm{OP}}(R,P) \tag{S10}$$

$$E_K(K_{in},K_{ex}) = \frac{\mathrm{RT}}{\mathrm{F}}\ln\frac{K_{ex}}{K_{in}} \tag{S11}$$

$$V_{0.5_{kir}}(K_{in},K_{ex}) = E_K + \Delta V_{kir} \tag{S12}$$

Table S3: Kir channel model parameters

Parameter	Value and Unit
$\bar{G}_{\mathrm{Kir}}$	2.75 nS/$\sqrt{\mathrm{mmol}}$
ΔV_{Kir}	20 mV
K_{Kir}	11 mV
$k_{Kir_{OP}}$	0.01

TRPC6 channel

$$\frac{dDAG}{dt} = -\frac{1}{\tau_{\mathrm{mct}}}\left(DAG - DAG_{\mathrm{SS}}\right) \tag{S13}$$

$$DAG_{\mathrm{SS}}(R,P) = \frac{\mathrm{DAG_{max}}}{1+\exp\left(-\mathrm{K_{DAG,IP3}}\cdot\left(\frac{R\cdot P}{h}-\frac{\mathrm{WS_{max}}}{2}\right)\right)} + \mathrm{DAG_{min}} \tag{S14}$$

TRPC6 was modeled as a DAG-gated, voltage-dependent nonspecific cation channel with bell-shaped Ca^{2+} dependence (activation and high-Ca inhibition) [3, 1]; we explicitly modeled Ca^{2+} influx through TRPC6 and its coupling to the WSTM, while Na^{+} permeation and additional modulators (e.g., PIP_2, PKC) were omitted for simplicity. The resulting Ca_{WSTM}, regulated by TRPC6-mediated Ca^{2+} influx, in turn modulates the activity of TRPM4 (Eq. S25) and TMEM16A (Eq. S30) channels.

$$S_{\mathrm{TRPC6\_DAG\_act}}(DAG) = \frac{DAG^{n_{\mathrm{TRPC6\_DAG}}}}{DAG^{n_{\mathrm{TRPC6\_DAG}}} + \mathrm{K_{TRPC6\_DAG}}^{\mathrm{n_{TRPC6\_DAG}}}} \tag{S15}$$

$$X_{\mathrm{TRPC6\_Ca\_act}}(Ca_{WSTM}) = \frac{Ca_{WSTM}^{\mathrm{n_{TRPC6\_Ca}}}}{Ca_{WSTM}^{n_{\mathrm{TRPC6\_Ca}}} + \mathrm{K_{TRPC6\_Ca\_act}}^{\mathrm{n_{TRPC6\_Ca}}}} \tag{S16}$$

$$Y_{\mathrm{TRPC6\_Ca\_inh}}(Ca_{WSTM}) = \frac{K_{\mathrm{TRPC6\_Ca\_inh}}^{n_{\mathrm{TRPC6\_Ca}}}}{K_{\mathrm{TRPC6\_Ca\_inh}}^{n_{\mathrm{TRPC6\_Ca}}} + Ca_{WSTM}^{n_{\mathrm{TRPC6\_Ca}}}} \tag{S17}$$

$$P_{\mathrm{TRPC6}}(Ca_{WSTM}, DAG) = S_{\mathrm{TRPC6\_DAG\_act}} \cdot X_{\mathrm{TRPC6\_Ca\_act}} \cdot Y_{\mathrm{TRPC6\_Ca\_inh}} \tag{S18}$$

$$J_{\mathrm{Ca\_TRPC6}}(v, Ca_{WSTM}, DAG) := \begin{cases} 10^6 \cdot \mathrm{TRPC6}_T \cdot \mathrm{perm}_{\mathrm{TRPC6}} \cdot P_{\mathrm{TRPC6}}(Ca_{WSTM}, DAG) \cdot \\ (\mathrm{Ca_{ex}} - Ca_{WSTM}), & \text{if } v = 0 \\ 10^6 \cdot \mathrm{TRPC6}_T \cdot \mathrm{perm}_{\mathrm{TRPC6}} \cdot P_{\mathrm{TRPC6}}(Ca_{WSTM}, DAG) \cdot \\ \quad z_{\mathrm{Ca}} \cdot \beta \cdot v \cdot \dfrac{(\mathrm{Ca_{ex}} - Ca_{WSTM} \cdot \exp(z_{\mathrm{Ca}} \cdot \beta \cdot v))}{\exp(z_{\mathrm{Ca}} \cdot \beta \cdot v) - 1}, & \text{if } v \neq 0 \end{cases} \tag{S19}$$

$$I_{\mathrm{TRPC6}}(v, Ca_{WSTM}, DAG) = \frac{-z_{\mathrm{Ca}} \cdot \mathrm{F} \cdot J_{\mathrm{Ca\_TRPC6}}(v, Ca_{WSTM}, DAG)}{1000} \tag{S20}$$

Table S4: TRPC6 channel model parameters[1]

Parameter	Value and Unit
$\mathrm{n_{TRPC6\_DAG}}$	2
$\mathrm{K_{TRPC6\_DAG}}$	1.38 µmol
$\mathrm{n_{TRPC6\_Ca\_M}}$	1
$\mathrm{K_{TRPC6\_Ca\_act}}$	1.7 µmol
$\mathrm{K_{TRPC6\_Ca\_inh}}$	1×10^1 µmol
$\mathrm{K_{DAG,IP3}}$	0.01
$\mathrm{DAG_{max}}$	0.1 µmol
$\mathrm{DAG_{min}}$	0.02 µmol
$\mathrm{TRPC6}_T$	2.5×10^2
$\mathrm{perm_{TRPC6}}$	$1.5 \times 10^{-13}\ \mathrm{cm^3\,s^{-1}}$

TRPM4 channel

$$\frac{dIP3}{dt} = -\frac{1}{\tau_{\mathrm{mct}}}\left(IP3 - IP3_{\mathrm{SS}}\right) \tag{S21}$$

$$IP3_{\mathrm{SS}}(R, P) = \frac{\mathrm{IP3_{max}}}{1 + \exp\left(-\mathrm{k_{DAG,IP3}} \cdot \left(\frac{R \cdot P}{h} - \frac{\mathrm{WS_{max}}}{2}\right)\right)} + \mathrm{IP3_{min}} \tag{S22}$$

Modeling the signaling pathways between IP3Rs and TRPM4 channels, where numerous microdomains must be considered for the occurrence of transient inward cation currents (TICCs) [7, 4], is computationally intensive. To simplify this, we assumed that an increase in IP3 concentration directly increases the steady-state open probability of TRPM4 channels. Additionally, studies have shown that Ca^{2+} entry through TRPC6 channels can further amplify Ca^{2+} release via IP3Rs on the sarcoplasmic reticulum

(SR), thereby enhancing TRPM4 channel activity and potentiating myogenic tone [7, 4]. Therefore:

$$X_{\mathrm{TRPM4\_act}}(Ca_{WSTM}) = \frac{Ca_{WSTM}^{\mathrm{n_{TRPM4\_Ca}}}}{Ca_{WSTM}^{\mathrm{n_{TRPM4\_Ca}}} + \mathrm{K}_{\mathrm{TRPM4\_Ca\_act}}^{\mathrm{n_{TRPM4\_Ca}}}} \tag{S23}$$

$$X_{\mathrm{IP3R}}(IP3) = \frac{IP3^{\mathrm{n_{TRPM4\_IP3}}}}{IP3^{\mathrm{n_{TRPM4\_IP3}}} + \mathrm{K}_{\mathrm{TRPM4\_IP3}}^{\mathrm{n_{TRPM4\_IP3}}}} \tag{S24}$$

$$P_{\mathrm{TRPM4}}(IP3, Ca_{WSTM}) = X_{\mathrm{TRPM4\_act}}(Ca_{WSTM}) \cdot X_{\mathrm{IP3R}}(IP3) \tag{S25}$$

$$J_{\mathrm{Na\_TRPM4}}(v, Ca_{WSTM}, IP3, Na_{\mathrm{in}}) := \begin{cases} 10^9 \cdot \mathrm{TRPM4}_T \cdot \mathrm{perm_{TRPM4}} \cdot P_{\mathrm{TRPM4}}(Ca_{WSTM}, IP3) \cdot (\mathrm{Na_{ex}} - Na_{\mathrm{in}}), & \text{if } v = 0 \\ 10^9 \cdot \mathrm{TRPM4}_T \cdot \mathrm{perm_{TRPM4}} \cdot P_{\mathrm{TRPM4}}(Ca_{WSTM}, IP3) \cdot z_{\mathrm{Na}} \cdot \beta \cdot v \cdot \frac{(\mathrm{Na_{ex}} - Na_{\mathrm{in}} \cdot \exp(z_{\mathrm{Na}} \cdot \beta \cdot v))}{\exp(z_{\mathrm{Na}} \cdot \beta \cdot v) - 1}, & \text{if } v \neq 0 \end{cases} \tag{S26}$$

$$I_{\mathrm{TRPM4}}(v, Ca_{WSTM}, IP3, Na_{in}) := \frac{-\mathrm{z_{Na}} \cdot \mathrm{F} \cdot J_{Na\_\mathrm{TRPM4}}(v, Ca_{WSTM}, IP3, Na_{in})}{1000} \tag{S27}$$

Table S5: TRPM4 channel model parameters

**Parameter**	**Value and Unit**
$\mathrm{n_{TRPM4\_Ca}}$	1
$\mathrm{K_{TRPM4\_Ca\_act}}$	10 µmol
$\mathrm{n_{TRPM4\_IP3}}$	2
$\mathrm{K_{TRPM4\_IP3}}$	1.38 µmol
$\mathrm{K_{DAG,IP3}}$	0.01
$\mathrm{IP3_{max}}$	0.1 µmol
$\mathrm{IP3_{min}}$	0.02 µmol
$\mathrm{TRPM4}_T$	50×10^2
$\mathrm{perm_{TRPM4}}$	$3.3 \times 10^{-14}\ \mathrm{cm^3\,s^{-1}}$

TMEM16A channel

The TMEM16A protein, also known as Anoctamin 1 (ANO1), forms a calcium-activated chloride channel (CaCC) that is activated by localized calcium increases, primarily regulated by nearby TRPC6 channels [8]. We

assumed that TMEM16A is also located within the WSTM. Therefore, its open probability is primarily regulated by Ca_{WSTM} and the membrane potential v:

$$X_{\mathrm{CaCC}}(v) = \frac{1}{1 + \exp(-\zeta_{\mathrm{CaCC}} \cdot \beta(v - \mathrm{V}_{\mathrm{CaCC}}))} \tag{S28}$$

$$K_{\mathrm{CaCC\_Ca\_act}}(v) = \frac{1}{\frac{1}{\mathrm{K}_{\mathrm{CaCC\_Ca\_act\_max}}} + X_{\mathrm{CaCC}}(v) \cdot \left(\frac{1}{\mathrm{K}_{\mathrm{CaCC\_Ca\_act\_min}}} - \frac{1}{\mathrm{K}_{\mathrm{CaCC\_Ca\_act\_max}}}\right)} \tag{S29}$$

$$P_{\mathrm{CaCC}}(Ca_{WSTM}, v) = \frac{Ca_{WSTM}^{\mathrm{n_{CaCC}}}}{Ca_{WSTM}^{\mathrm{n_{CaCC}}} + K_{\mathrm{CaCC\_Ca\_act}}^{\mathrm{n_{CaCC}}}} \cdot \frac{\mathrm{K}_{\mathrm{CaCC\_Ca\_inh}}^{\mathrm{n_{CaCC}}}}{Ca_{WSTM}^{\mathrm{n_{CaCC}}} + \mathrm{K}_{\mathrm{CaCC\_Ca\_inh}}^{\mathrm{n_{CaCC}}}} \tag{S30}$$

$$J_{\mathrm{CaCC}}(v, Ca_{WSTM}, Cl_{\mathrm{in}}) := \begin{cases} 10^9 \cdot \mathrm{CaCC}_T \cdot \mathrm{perm}_{\mathrm{CaCC}} \cdot P_{\mathrm{CaCC}}(Ca_{WSTM}, v) \cdot \\ (Cl_{\mathrm{ex}} - Cl_{\mathrm{in}}), & \text{if } v = 0 \\ 10^9 \cdot \mathrm{CaCC}_T \cdot \mathrm{perm}_{\mathrm{CaCC}} \cdot P_{\mathrm{CaCC}}(Ca_{WSTM}, v) \cdot z_{\mathrm{Cl}} \cdot \beta \cdot v \cdot \\ \qquad \frac{Cl_{\mathrm{ex}} - Cl_{\mathrm{in}} \exp(z_{\mathrm{Cl}} \cdot \beta \cdot v)}{\exp(z_{\mathrm{Cl}} \cdot \beta \cdot v) - 1}, & \text{if } v \neq 0 \end{cases} \tag{S31}$$

$$I_{\mathrm{CaCC}}\left(v, Ca_{WSTM}, Cl_{in}\right) := \frac{-\mathrm{z}_{\mathrm{Cl}} \cdot \mathrm{F} \cdot J_{\mathrm{CaCC}}\left(v, Ca_{WSTM}, Cl_{in}\right)}{1000} \tag{S32}$$

Table S6: TMEM16A (CaCC) channel model parameters[1]

**Parameter**	**Value and Unit**
ζ_{CaCC}	2
$\mathrm{V}_{\mathrm{CaCC}}$	0 mV
$\mathrm{n}_{\mathrm{CaCC}}$	2.6
$\mathrm{K}_{\mathrm{CaCC\_Ca\_act\_max}}$	4.1 µmol
$\mathrm{K}_{\mathrm{CaCC\_Ca\_act\_min}}$	0.3 µmol
$\mathrm{K}_{\mathrm{CaCC\_Ca\_inh}}$	4×10^1 µmol
$\mathrm{perm}_{\mathrm{CaCC}}$	1.6×10^{-14} $\mathrm{cm^3\,s^{-1}}$
CaCC_T	2.5×10^3

KATP Channel

The ATP-sensitive potassium ($\mathrm{K_{ATP}}$) channel is expressed at lower levels in aSMCs and aECs compared to its higher prevalence in capillary vascu-

lar cells [9]. Attempts to induce dilation in isolated PAs through pharmacological activation of these channels have been largely ineffective [10, 11]. Research suggests that pericytes exhibit a nearly *all-or-none* response to energy substrates, indicating a binary-like behavior in which the channels are either almost entirely open or completely closed [9]. Building on these findings, we incorporated the K_{ATP} channel into our model under the assumption of complete closure, setting the open probability ($P_{open\_K_{ATP}}$) to zero.

$$J_{K_{ATP}}(v, K_{in}) := \begin{cases} 10^9 \cdot K_{ATP,T} \cdot \mathrm{perm}_{K_{ATP}} \cdot P_{open\_K_{ATP}} \cdot (K_{ex} - K_{in}), & \text{if } v = 0 \\ 10^9 \cdot K_{ATP,T} \cdot \mathrm{perm}_{K_{ATP}} \cdot P_{open\_K_{ATP}} \cdot z_K \cdot \beta \cdot v \cdot \dfrac{K_{ex} - K_{in}\exp(z_K \cdot \beta \cdot v)}{\exp(z_K \cdot \beta \cdot v) - 1}, & \text{if } v \neq 0 \end{cases} \tag{S33}$$

$$I_{K_{ATP}}(v, K_{in}) := \frac{-z_K \cdot F \cdot J_{K_{ATP}}(v, K_{in})}{1000} \tag{S34}$$

Table S7: K_{ATP} channel model parameters[1]

**Parameter**	**Value and Unit**
$\mathrm{perm}_{K_{ATP}}$	7×10^{-14} cm^3 s^{-1}
$K_{ATP}T$	3×10^2
$P_{open\_K_{ATP}}$	0

Leak channels

$$J_{Cl\_leak}(v, Cl_{in}) = \begin{cases} 10^9 \cdot Cl_{leakT} \cdot \mathrm{perm}_{Cl_{leak}} \cdot (Cl_{ex} - Cl_{in}) & \text{if } v = 0 \\ 10^9 \cdot Cl_{leakT} \cdot \mathrm{perm}_{Cl_{leak}} \cdot z_{Cl} \cdot \beta \cdot v \frac{(Cl_{ex} - Cl_{in} \cdot \exp(z_{Cl} \cdot \beta \cdot v))}{\exp(z_{Cl} \cdot \beta \cdot v) - 1} & \text{if } v \neq 0 \end{cases} \tag{S35}$$

$$J_{K\_leak}(v, K_{in}) = \begin{cases} 10^9 \cdot K_{leakT} \cdot \mathrm{perm}_{K_{leak}} \cdot (K_{ex} - K_{in}) & \text{if } v = 0 \\ 10^9 \cdot K_{leakT} \cdot \mathrm{perm}_{K_{leak}} \cdot z_K \cdot \beta \cdot v \frac{(K_{ex} - K_{in} \cdot \exp(z_K \cdot \beta \cdot v))}{\exp(z_K \cdot \beta \cdot v) - 1} & \text{if } v \neq 0 \end{cases} \tag{S36}$$

$$J_{Na\_leak}(v, Na_{in}) = \begin{cases} 10^9 \cdot Na_{leakT} \cdot \mathrm{perm}_{Na_{leak}} \cdot (Na_{ex} - Na_{in}) & \text{if } v = 0 \\ 10^9 \cdot Na_{leakT} \cdot \mathrm{perm}_{Na_{leak}} \cdot z_{Na} \cdot \beta \cdot v \\ \frac{(Na_{ex} - Na_{in} \cdot \exp(z_{Na} \cdot \beta \cdot v))}{\exp(z_{Na} \cdot \beta \cdot v) - 1} & \text{if } v \neq 0 \end{cases} \tag{S37}$$

$$J_{\text{Ca\_leak}}\left(v, Ca_{in}\right) = \begin{cases} 10^6 \cdot \text{Ca}_{\text{leakT}} \cdot \text{perm}_{\text{Ca}_{\text{leak}}} \cdot (\text{Ca}_{\text{ex}} - Ca_{in}) & \text{if } v = 0 \\ 10^6 \cdot \text{Ca}_{\text{leakT}} \cdot \text{perm}_{\text{Ca}_{\text{leak}}} \cdot \text{z}_{\text{Ca}} \cdot \beta \cdot v & \\ \frac{(\text{Ca}_{\text{ex}} - Ca_{in} \cdot \exp(\text{z}_{\text{Ca}} \cdot \beta \cdot v))}{\exp(\text{z}_{\text{Ca}} \cdot \beta \cdot v) - 1} & \text{if } v \neq 0 \end{cases} \tag{S38}$$

$$\begin{aligned} I_{\text{Cl\_leak}}\left(v, Cl_{in}\right) &= \frac{-\text{z}_{\text{Cl}} \cdot \text{F} \cdot I_{\text{Cl\_leak}}\left(v, Cl_{in}\right)}{1000} \\ I_{\text{K\_leak}}\left(v, K_{in}\right) &= \frac{-\text{z}_{\text{K}} \cdot \text{F} \cdot J_{\text{K\_leak}}\left(v, K_{in}\right)}{1000} \\ I_{\text{Na\_leak}}\left(v, Na_{in}\right) &= \frac{-\text{z}_{\text{Na}} \cdot \text{F} \cdot J_{\text{Na\_leak}}\left(v, Na_{in}\right)}{1000} \\ I_{\text{Ca\_leak}}\left(v, Ca_{in}\right) &= \frac{-\text{z}_{\text{Ca}} \cdot \text{F} \cdot J_{\text{Ca\_leak}}\left(v, Ca_{in}\right)}{1000} \end{aligned} \tag{S39}$$

$$\begin{aligned} I_{\text{leak}_{ALL}}\left(v, K_{in}, Cl_{in}, Ca_{in}, Na_{in}\right) := {} & I_{\text{K\_leak}}\left(v, K_{in}\right) + I_{\text{Cl\_leak}}\left(v, Cl_{in}\right) \\ & + I_{\text{Ca\_leak}}\left(v, Ca_{in}\right) + I_{\text{Na\_leak}}\left(v, Na_{in}\right) \end{aligned} \tag{S40}$$

Table S8: Leak currents model parameters[1]

**Parameter**	**Value and Unit**
Cl_{leakT}	22
$\text{perm}_{\text{Cl}_{\text{leak}}}$	1×10^{-15} $\text{cm}^3\,\text{s}^{-1}$
Na_{leakT}	0
$\text{perm}_{\text{Na}_{\text{leak}}}$	1×10^{-15} $\text{cm}^3\,\text{s}^{-1}$
K_{leakT}	20
$\text{perm}_{\text{K}_{\text{leak}}}$	1×10^{-15} $\text{cm}^3\,\text{s}^{-1}$
Ca_{leakT}	13
$\text{perm}_{\text{Ca}_{\text{leak}}}$	1×10^{-14} $\text{cm}^3\,\text{s}^{-1}$

Na,K ATPase pump

$$rel\Delta\mu_{\text{NaK}}(v, K_{in}, Na_{in}) = \frac{3 \cdot \text{R} \cdot \text{T} \cdot \ln\left(\frac{\text{Na}_{\text{ex}}}{Na_{in}}\right) + 2 \cdot \text{R} \cdot \text{T} \cdot \ln\left(\frac{K_{in}}{\text{K}_{\text{ex}}}\right) - \left(\frac{\text{F} \cdot v}{1000}\right) + \Delta\mu_{\text{ATP}}}{\Delta\mu_{\text{ATP}}} \tag{S41}$$

$$I_{\mathrm{NaK}}(v, Na_{in}, K_{in}) = \mathrm{I}_{\mathrm{NaK}_{\mathrm{max}}} \cdot \frac{\mathrm{K_{ex}}^{\mathrm{n}_{\mathrm{NaK_K}}}}{(\mathrm{K_{ex}}^{\mathrm{n}_{\mathrm{NaK_K}}} + \mathrm{K}_{\mathrm{NaK_{K_{ex}}}}{}^{\mathrm{n}_{\mathrm{NaK_K}}})} \cdot \frac{Na_{in}{}^{\mathrm{n}_{\mathrm{NaK_{Na}}}}}{(Na_{in}{}^{\mathrm{n}_{\mathrm{NaK_{Na}}}} + \mathrm{K}_{\mathrm{NaK_{Na_{in}}}}{}^{\mathrm{n}_{\mathrm{NaK_{Na}}}})} \cdot rel\Delta\mu_{\mathrm{NaK}}(v, K_{in}, Na_{in}) \tag{S42}$$

Table S9: Na,K ATPase model parameters[1]

**Parameter**	**Value and Unit**
$\Delta\mu_{\mathrm{ATP}}$	-5×10^4 J mol^{-1}
$\mathrm{n}_{\mathrm{NaK_K}}$	1.1
$\mathrm{K}_{\mathrm{NaK_{K_{ex}}}}$	1.6 mM
$\mathrm{n}_{\mathrm{NaK_{Na}}}$	2.5
$\mathrm{K}_{\mathrm{NaK_{Na_{in}}}}$	22 mM
$\mathrm{I}_{\mathrm{NaK_{max}}}$	60 pA

NaKCl cotransporter

$$\alpha_{NaKCl_0} = \frac{\beta_{\mathrm{NaKCl_0}} \cdot \alpha_{\mathrm{NaKCl_4}}}{\beta_{\mathrm{NaKCl_4}}} \tag{S43}$$

$$Q(Na_{in}, K_{in}, Cl_{in}) = \frac{\alpha_{\mathrm{NaKCl_4}} + \alpha_{\mathrm{NaKCl_0}} \cdot \frac{\mathrm{L_{NaKCl_{Na}}} \cdot \mathrm{L_{NaKCl_K}} \cdot \mathrm{L_{NaKCl_{Cl}}}^2}{\mathrm{Na_{ex}} \cdot \mathrm{K_{ex}} \cdot \mathrm{Cl_{ex}}^2}}{\beta_{\mathrm{NaKCl_4}} + \beta_{\mathrm{NaKCl_0}} \cdot \frac{\mathrm{L_{NaKCl_{Na}}} \cdot \mathrm{L_{NaKCl_K}} \cdot \mathrm{L_{NaKCl_{Cl}}}^2}{Na_{in} \cdot K_{in} \cdot Cl_{in}{}^2}} \tag{S44}$$

$$D_1 = 1 + \frac{\mathrm{L_{NaKCl_{Cl}}}}{\mathrm{Cl_{ex}}} + \frac{\mathrm{L_{NaKCl_{Cl}}} \cdot \mathrm{L_{NaKCl_K}}}{\mathrm{Cl_{ex}} \cdot \mathrm{K_{ex}}} + \frac{\mathrm{L_{NaKCl_{Cl}}}^2 \cdot \mathrm{L_{NaKCl_K}}}{\mathrm{Cl_{ex}}^2 \cdot \mathrm{K_{ex}}} + \frac{\mathrm{L_{NaKCl_{Cl}}}^2 \cdot \mathrm{L_{NaKCl_{Na}}} \cdot \mathrm{L_{NaKCl_K}}}{\mathrm{Cl_{ex}}^2 \cdot \mathrm{Na_{ex}} \cdot \mathrm{K_{ex}}} \tag{S45}$$

$$D_2(K_{in}, Na_{in}, Cl_{in}) = 1 + \frac{\mathrm{L_{NaKCl_{Na}}}}{Na_{in}} + \frac{\mathrm{L_{NaKCl_{Cl}}} \cdot \mathrm{L_{NaKCl_{Na}}}}{Cl_{in} \cdot Na_{in}} + \frac{\mathrm{L_{NaKCl_{Cl}}} \cdot \mathrm{L_{NaKCl_K}} \cdot \mathrm{L_{NaKCl_{Na}}}}{Cl_{in} \cdot K_{in} \cdot Na_{in}} + \frac{\mathrm{L_{NaKCl_{Cl}}}^2 \cdot \mathrm{L_{NaKCl_{Na}}} \cdot \mathrm{L_{NaKCl_K}}}{Cl_{in}{}^2 \cdot Na_{in} \cdot K_{in}} \tag{S46}$$

$$J_{4\_NaKCl_{ALL}}(Na_{in}, Cl_{in}, K_{in}) = \frac{\mathrm{NaKCl_T}}{D_1 + Q(Na_{in}, K_{in}, Cl_{in}) \cdot D_2(K_{in}, Na_{in}, Cl_{in})} \cdot (\alpha_{\mathrm{NaKCl_4}} - \beta_{\mathrm{NaKCl_4}} \cdot Q(Na_{in}, K_{in}, Cl_{in})) \tag{S47}$$

$$\begin{aligned} J_{\mathrm{NaK\_Cl}}(Na_{in}, Cl_{in}, K_{in}) &= \frac{2 \times 10^{15}}{\mathrm{NA}} \cdot J_{4\_\mathrm{NaKCl_{ALL}}} \\ J_{\mathrm{KCl\_Na}}(Na_{in}, Cl_{in}, K_{in}) &= \frac{1 \times 10^{15}}{\mathrm{NA}} \cdot J_{4\_\mathrm{NaKCl_{ALL}}} \\ J_{\mathrm{NaCl\_K}}(Na_{in}, Cl_{in}, K_{in}) &= \frac{1 \times 10^{15}}{\mathrm{NA}} \cdot J_{4\_\mathrm{NaKCl_{ALL}}} \end{aligned} \tag{S48}$$

$$I_{\mathrm{NaK\_Cl}}\left(Na_{in}, Cl_{in}, K_{in}\right) := \frac{-\mathrm{z_{Cl}} \cdot \mathrm{F} \cdot J_{\mathrm{NaK\_Cl}}(Na_{in}, Cl_{in}, K_{in})}{1000} \tag{S49}$$

Table S10: NaKCl cotransporter model parameters[1]

**Parameter**	**Value and Unit**
$\beta_{\mathrm{NaKCl_0}}$	5×10^4 $\mathrm{s^{-1}}$
$\alpha_{\mathrm{NaKCl_4}}$	5×10^4 $\mathrm{s^{-1}}$
$\beta_{\mathrm{NaKCl_4}}$	4×10^4 $\mathrm{s^{-1}}$
NA	6.022×10^{23}
$\mathrm{L_{NaKCl_{Na}}}$	3.2×10^1 mM
$\mathrm{L_{NaKCl_K}}$	2.7×10^1 mM
$\mathrm{L_{NaKCl_{Cl}}}$	6.3×10^1 mM
NaKCl_T	1×10^4 pA

L-Type VOCC

$$X_{CaV_a}(v) := \frac{1}{1 + \exp\left(-\frac{v - V_{CaV_{act}}}{\xi_{CaV_{act}}}\right)} \tag{S50}$$

$$Y_{CaV_i}(v, Ca_{\mathrm{in}}) := \frac{1}{1 + \frac{Ca_{\mathrm{in}}}{\mathrm{K_{CaV_{inh}}}}} \tag{S51}$$

$$P_{CaV}(v, Ca_{\mathrm{in}}) := X_{CaV_a}(v) \cdot Y_{CaV_i}(v, Ca_{\mathrm{in}}) \tag{S52}$$

$$J_{\mathrm{CaV}}(v, Ca_{\mathrm{in}}) := \begin{cases} 10^6 \cdot \mathrm{CaV}_T \cdot \mathrm{perm}_{\mathrm{CaV}} \cdot P_{\mathrm{CaV}}(v) \cdot (Ca_{\mathrm{ex}} - Ca_{\mathrm{in}})\,, & \text{if } v = 0 \\ 10^6 \cdot \mathrm{CaV}_T \cdot \mathrm{perm}_{\mathrm{CaV}} \cdot P_{\mathrm{CaV}}(v) \cdot z_{\mathrm{Ca}} \cdot \beta \cdot v \cdot \dfrac{Ca_{\mathrm{ex}} - Ca_{\mathrm{in}} \exp(z_{\mathrm{Ca}} \cdot \beta \cdot v)}{\exp(z_{\mathrm{Ca}} \cdot \beta \cdot v) - 1}, & \text{if } v \neq 0 \end{cases} \tag{S53}$$

$$I_{\mathrm{CaV}}(v, Ca_{\mathrm{in}}) := \frac{-\mathrm{z}_{\mathrm{Ca}} \cdot \mathrm{F} \cdot J_{\mathrm{CaV}}(v, Ca_{\mathrm{in}})}{1000} \tag{S54}$$

Table S11: L-Type VOCC model parameters[1]

**Parameter**	**Value and Unit**
$\mathrm{V}_{\mathrm{CaV}_{\mathrm{act}}}$	6.2 mV
$\xi_{\mathrm{CaV}_{\mathrm{act}}}$	9.3 mV
$\mathrm{K}_{\mathrm{CaV}_{\mathrm{inh}}}$	1.2 µmol
CaV_T	3×10^3
$\mathrm{perm}_{\mathrm{CaV}}$	$1.21 \times 10^{-13}\ \mathrm{cm^3\,s^{-1}}$

Na/Ca exchanger (NCX)

$$\beta_{\mathrm{NCX\_0}} = \frac{\alpha_{\mathrm{NCX\_0}} \cdot \beta_{\mathrm{NCX\_4}}}{\alpha_{\mathrm{NCX\_4}}} \tag{S55}$$

$$Z_{\mathrm{NCX\_act}}(Ca_{in}) = \frac{Ca_{\mathrm{in}}^{\mathrm{n\_NCX\_act}}}{\mathrm{K}_{\mathrm{NCX\_act\_Ca}}^{\mathrm{n\_NCX\_act}} + Ca_{\mathrm{in}}^{\mathrm{n\_NCX\_act}}} \tag{S56}$$

$$QQ(v, Ca_{in}, Na_{in}) = \frac{\alpha_{\mathrm{NCX\_4}} + \alpha_{\mathrm{NCX\_0}} \cdot \frac{\mathrm{K}_{\mathrm{NCX\_Ca}} \cdot \mathrm{K}^3_{\mathrm{NCX\_Na}} \cdot \exp(\frac{\mathrm{F} \cdot v}{2000 \cdot \mathrm{R} \cdot \mathrm{T}})}{Ca_{\mathrm{in}} \cdot \mathrm{Na}^3_{ex}}}{\beta_{\mathrm{NCX\_4}} + \beta_{\mathrm{NCX\_0}} \cdot \frac{\mathrm{K}_{\mathrm{NCX\_Ca}} \cdot \mathrm{K}^3_{\mathrm{NCX\_Na}} \cdot \exp(-\frac{\mathrm{F} \cdot v}{2000 \cdot \mathrm{R} \cdot \mathrm{T}})}{\mathrm{Ca_{ex}} \cdot Na^3_{\mathrm{in}}}} \tag{S57}$$

$$DD_1(Ca_{in}) = 1 + \frac{\mathrm{K}_{\mathrm{NCX\_Ca}}}{Ca_{\mathrm{in}}} + \frac{3 \cdot \mathrm{K}_{\mathrm{NCX\_Na}} \cdot \mathrm{K}_{\mathrm{NCX\_Ca}}}{\mathrm{Na}_{ex} \cdot Ca_{\mathrm{in}}} + \frac{3 \cdot \mathrm{K}^2_{\mathrm{NCX\_Na}} \cdot \mathrm{K}_{\mathrm{NCX\_Ca}}}{\mathrm{Na}^2_{\mathrm{ex}} \cdot Ca_{\mathrm{in}}} + \frac{\mathrm{K}^3_{\mathrm{NCX\_Na}} \cdot \mathrm{K}_{\mathrm{NCX\_Ca}}}{\mathrm{Na}^3_{\mathrm{ex}} \cdot Ca_{\mathrm{in}}} \tag{S58}$$

$$DD_2(Na_{in}) = 1 + \frac{\mathrm{K}_{\mathrm{NCX\_Ca}}}{\mathrm{Ca_{ex}}} + \frac{3 \cdot \mathrm{K}_{\mathrm{NCX\_Na}} \cdot \mathrm{K}_{\mathrm{NCX\_Ca}}}{\mathrm{Ca_{ex}} \cdot Na_{\mathrm{in}}} + \frac{3 \cdot \mathrm{K}^2_{\mathrm{NCX\_Na}} \cdot \mathrm{K}_{\mathrm{NCX\_Ca}}}{\mathrm{Ca_{ex}} \cdot Na^2_{\mathrm{in}}} + \frac{\mathrm{K}^3_{\mathrm{NCX\_Na}} \cdot \mathrm{K}_{\mathrm{NCX\_Ca}}}{\mathrm{Ca_{ex}} \cdot Na^3_{\mathrm{in}}} \tag{S59}$$

$$S_4(v, Ca_{\mathrm{in}}, Na_{\mathrm{in}}) = (\alpha_{\mathrm{NCX4}} - QQ(v, Ca_{\mathrm{in}}, Na_{\mathrm{in}}) \cdot \beta_{\mathrm{NCX4}}) \cdot \frac{Z_{\mathrm{NCX,act}}(Ca_{\mathrm{in}}) \cdot \mathrm{NCX}_T}{DD_1(Ca_{\mathrm{in}}) + QQ(v, Ca_{\mathrm{in}}, Na_{\mathrm{in}}) \cdot DD_2(Na_{\mathrm{in}})} \tag{S60}$$

$$I_{\mathrm{NCX}}(v, Ca_{in}, Na_{in}) = \frac{-1 \times 10^{12} \cdot \mathrm{F}}{\mathrm{NA}} \cdot S_4(v, Ca_{in}, Na_{in}) \tag{S61}$$

Table S12: Na/Ca exchanger (NCX) model parameters[1]

**Parameter**	**Value and Unit**
$\alpha_{\mathrm{NCX\_0}}$	3×10^4 s^{-1}
$\beta_{\mathrm{NCX\_4}}$	5×10^4 s^{-1}
$\alpha_{\mathrm{NCX\_4}}$	3×10^4 s^{-1}
$K_{\mathrm{NCX\_act\_Ca}}$	1×10^{-1} µmol
$\mathrm{n}_{\mathrm{NCX\_act}}$	2
$\mathrm{K}_{\mathrm{NCX\_Ca}}$	8×10^{-2} µmol
$\mathrm{K}_{\mathrm{NCX\_Na}}$	1×10^2 mM
NCX_T	1.6×10^3

Plasma membrane calcium ATPase (PMCA)

$$rel\Delta\mu_{\mathrm{PMCA}}(v, Ca_{in}) = \frac{-\frac{\mathrm{F} \cdot v}{1000} + \mathrm{R} \cdot \mathrm{T} \cdot \ln \frac{\mathrm{Ca_{ex}}}{Ca_{\mathrm{in}}} + \Delta\mu_{\mathrm{ATP}}}{\Delta\mu_{\mathrm{ATP}}} \tag{S62}$$

$$I_{\mathrm{PMCA}}(v, Ca_{in}) = \mathrm{I_{PMCA\_max}} \cdot \frac{Ca_{\mathrm{in}}^{\mathrm{n_{PMCA\_Ca}}}}{Ca_{\mathrm{in}}^{\mathrm{n_{PMCA\_Ca}}} + \mathrm{K_{PMCA\_Ca}^{n_{PMCA\_Ca}}}} \cdot rel\Delta\mu_{\mathrm{PMCA}}(v, Ca_{in}) \tag{S63}$$

Table S13: PMCA model parameters[1]

**Parameter**	**Value and Unit**
$\Delta\mu_{\mathrm{ATP}}$	-5×10^4 J mol^{-1}
$\mathrm{I}_{\mathrm{PMCA\_max}}$	18 pA
$\mathrm{n}_{\mathrm{PMCA\_Ca}}$	3
$\mathrm{K}_{\mathrm{PMCA\_Ca}}$	0.4 µmol

Sarcoplasmic reticulum calcium ATPase (SERCA)

$$rel_{SERCA}(Ca_{in}) = \frac{Ca_{\mathrm{in}}^{\mathrm{n_{SERCA}}}}{Ca_{\mathrm{in}}^{\mathrm{n_{SERCA}}} + \mathrm{K}_{\mathrm{SERCA\_Ca}}^{\mathrm{n_{SERCA}}}} \cdot \frac{1}{1 + \left(\frac{\mathrm{Ca_{SRcen}}}{\mathrm{K_{SERCA_{inh_{Ca_{SRcen}}}}}}\right)^{\mathrm{n_{SERCA}}}} \tag{S64}$$

$$rel\Delta\mu_{\mathrm{SERCA}}(Ca_{in}) = \frac{(2 \cdot \mathrm{R} \cdot \mathrm{T} \cdot \ln(\frac{\mathrm{Ca_{SRcen}}}{Ca_{\mathrm{in}}})) + \Delta\mu_{\mathrm{ATP}}}{\Delta\mu_{\mathrm{ATP}}} \tag{S65}$$

$$J_{\mathrm{SERCA}}(Ca_{in}) = -\frac{10^3}{\mathrm{F}} \cdot \mathrm{I_{SERCA\_max}} \cdot rel_{SERCA}(Ca_{in}) \cdot rel\Delta\mu_{\mathrm{SERCA}}(Ca_{in}) \tag{S66}$$

$$I_{\mathrm{SERCA}}(Ca_{in}) := \frac{-\mathrm{F} \cdot J_{\mathrm{SERCA}}(Ca_{in}))}{1000} \tag{S67}$$

Table S14: SERCA model parameters[1]

**Parameter**	**Value and Unit**
$\mathrm{n_{SERCA}}$	1.6
$\mathrm{Ca_{SRcen}}$	100 µmol
$\mathrm{K_{SERCA\_Ca}}$	0.2 µmol
$\mathrm{K_{SERCA_{inh_{Ca_{SRcen}}}}}$	1×10^2 µmol
$\Delta\mu_{\mathrm{ATP}}$	$-5 \times 10^4\ \mathrm{J\,mol^{-1}}$
$\mathrm{I_{SERCA_{max}}}$	8×10^{-1} pA

Calcium flux coupling and buffering

$$J_{Ca_{\text{BK-Glu\_}cyt}}(Ca_{\text{BK-Glu}}, Ca_{in}) = 10^9 \cdot \lambda_{Ca_{\text{BK-Glu\_}cyt}} \cdot (Ca_{\text{Bk-Glu}} - Ca_{in}) \tag{S68}$$

$$J_{Ca_{\text{WSTM\_}cyt}}(Ca_{\text{WSTM}}, Ca_{in}) = 10^9 \cdot \lambda_{Ca_{\text{WSTM\_}cyt}} \cdot (Ca_{\text{WSTM}} - Ca_{in}) \tag{S69}$$

$$\frac{d\,BUF_1}{dt} = \mathrm{K_{BUF\_on}} \cdot Ca_{in} \cdot (\mathrm{BUF}_T - BUF_1) - \mathrm{K_{BUF\_off}} \cdot BUF_1 \tag{S70}$$

$$\frac{d\,\mathrm{BUF}_M}{dt} = \mathrm{K_{BUF\_on}} \cdot Ca_{WSTM} \cdot (\mathrm{BUF}_{M_T} - \mathrm{BUF}_M) - \mathrm{K_{BUF\_off}} \cdot \mathrm{BUF}_M \tag{S71}$$

Table S15: Ca^{2+} flux coupling and buffering parameters[1]

**Parameter**	**Value and Unit**
$\lambda_{Ca_{\text{BK-Glu\_cyt}}}$	4×10^{-13}
$\lambda_{Ca_{\text{WSTM\_cyt}}}$	1.6×10^{-13}
$\mathrm{K_{BUF\_on}}$	2.2×10^{1} µmol^{-1} s^{-1}
$\mathrm{K_{BUF\_off}}$	7.7×10^{1} s^{-1}
BUF_T	3×10^{2} µmol
BUF_{M_T}	3×10^{2} µmol

NMDA receptor-mediated calcium influx

NMDA receptor activation was modeled as a glutamate-dependent gating mechanism that regulates Ca^{2+} influx into the Glu–BK microdomain. The open probability was described by a Hill function:

$$P_{\text{NMDA}}(Glu) = \frac{Glu^{n_{\text{GluR}}}}{Glu^{n_{\text{GluR}}} + \mathrm{K}_{\text{GluR}}^{n_{\text{GluR}}}} \tag{S72}$$

$$J_{\text{Ca}_{\text{NMDA}}}(v, Glu, Ca_{\text{in}}, Ca_{\text{ex}}) := \begin{cases} 10^6 \cdot \text{Ca}_{\text{NMDA}T} \cdot \text{perm}_{\text{Ca}_{\text{NMDA}}} \cdot P_{\text{NMDA}}(Glu, v) \cdot (Ca_{\text{ex}} - Ca_{\text{in}}), & \text{if } v = 0 \\ 10^6 \cdot \text{Ca}_{\text{NMDA}T} \cdot \text{perm}_{\text{Ca}_{\text{NMDA}}} \cdot P_{\text{NMDA}}(Glu, v) \cdot z_{\text{Ca}} \cdot \beta \cdot v \cdot \\ \qquad \dfrac{Ca_{\text{ex}} - Ca_{\text{in}} \exp(z_{\text{Ca}} \cdot \beta \cdot v)}{\exp(z_{\text{Ca}} \cdot \beta \cdot v) - 1}, & \text{if } v \neq 0 \end{cases} \tag{S73}$$

$$I_{\text{Ca}_{\text{NMDA}}} = -\frac{\mathrm{z_{Ca}} \cdot \mathrm{F} \cdot J_{\text{Ca}_{\text{NMDA}}}}{1000} \tag{S74}$$

Table S16: NMDA receptor model parameters

**Parameter**	**Value and Unit**
$\text{Ca}_{\text{NMDA}_T}$	0.8×10^{-2}
$\text{perm}_{\text{Ca}_{\text{NMDA}}}$	1.5×10^{-13} cm^3 s^{-1}
$\mathrm{K_{GluR}}$	1.38 µmol
$\mathrm{n_{GluR}}$	2

aEC Kir channel[5]

$$\frac{dKir_{EC\text{OP}}}{dt} = -\frac{1}{\tau_{\text{mct}}} \left(Kir_{EC\text{OP}} - Kir_{EC\text{OP\_SS}}\right) \tag{S75}$$

$$Kir_{EC\text{OP\_SS}}(WSS) = \frac{1}{1 + \exp\left(-\mathrm{k}_{Kir_{EC_{OP}}} \cdot \left(WSS - \frac{\mathrm{WSS_{max}}}{2}\right)\right)} \tag{S76}$$

$$I_{Kir_{EC}}(WSS, v_{EC}, K_{ex_{EC}}) = \frac{\bar{\mathrm{G}}_{Kir_{EC}} \sqrt{K_{ex_{EC}}}\,(v_{EC} - E_{KEC})}{1 + \exp\left(\frac{v_{EC} - V_{0.5_{kir_{EC}}}}{\mathrm{k_{kir_{EC}}}}\right)} \cdot Kir_{EC\text{OP}}(WSS) \tag{S77}$$

$$E_{KEC}(K_{in}, K_{ex_{EC}}) = \frac{\mathrm{RT}}{\mathrm{F}} \ln \frac{K_{ex_{EC}}}{K_{in}} \tag{S78}$$

$$V_{0.5_{kir_{EC}}}(K_{in}, K_{ex_{EC}}) = E_{KEC} + \Delta V_{kir_{EC}} \tag{S79}$$

Table S17: aEC Kir channel model parameters

Parameter	Value and Unit
$\mathrm{k_{Kir_{EC\_OP}}}$	5.25
$\bar{\mathrm{G}}_{\mathrm{Kir_{EC}}}$	4.8 $\mathrm{nS}/\sqrt{\mathrm{mmol}}$
$\mathrm{k_{kir_{EC}}}$	12 mV
$\Delta V_{\mathrm{kir_{EC}}}$	4.65 mV

Gap junctions

$$I_{SMC-SMC}(v) = \frac{1}{\mathrm{R_{gj_{SMC-SMC}}}} \cdot (v - v_{adj}) \tag{S80}$$

$$I_{EC-EC}(v_{\mathrm{EC}}) = \frac{1}{\mathrm{R_{gj_{EC-EC}}}} \cdot (v_{\mathrm{EC}} - v_{\mathrm{EC}_{adj}}) \tag{S81}$$

$$I_{\text{SMC-EC}} = \frac{1}{\mathrm{R_{gj\_SMC\text{-}EC}}} \cdot (v - v_{\mathrm{EC}}) \tag{S82}$$

Table S18: Gap junction resistance parameters[5]

Parameter	Value and Unit
$\mathrm{R_{gj_{SMC\text{-}SMC}}}$	100 MΩ
$\mathrm{R_{gj_{EC\text{-}EC}}}$	100 MΩ
$\mathrm{R_{gj_{SMC\text{-}EC}}}$	1 GΩ

Differential equations

$$\frac{dv}{dt} = -\frac{10^{-9}}{\mathrm{C}_m} \cdot (I_{\mathrm{BK}} + I_{CaV} + I_{KATP} + I_{TRPM4} + I_{TRPC6} + I_{CaCC} + I_{Kv} + I_{Kir} + I_{PMCA} + I_{NCX} + I_{NaK} + I_{\mathrm{leak}_{ALL}} + I_{\text{SMC-EC}} + I_{SMC-SMC}) \quad \text{(S83)}$$

$$\frac{dCl_{in}}{dt} = (-\frac{10^{-9}}{\mathrm{z}_{Cl} \cdot \mathrm{F} \cdot \mathrm{VOL\_cell}}) \cdot (I_{CaCC} + I_{\mathrm{Cl\_leak}} + (\frac{10^{-12}}{\mathrm{VOL\_cell}}) \cdot J_{\mathrm{NaK\_Cl}} \quad \text{(S84)}$$

$$\frac{dNa_{in}}{dt} = (-\frac{10^{-9}}{\mathrm{z}_{Na} \cdot \mathrm{F} \cdot \mathrm{VOL\_cell}}) \cdot (I_{\mathrm{TRPM4}} + 3 \cdot I_{\mathrm{NCX}} + 3 \cdot I_{\mathrm{NaK}} + I_{\mathrm{Na\_leak}}) + (\frac{10^{-12}}{\mathrm{VOL\_cell}}) \cdot J_{\mathrm{KCl\_Na}} \quad \text{(S85)}$$

$$\frac{dK_{in}}{dt} = (-\frac{10^{-9}}{\mathrm{z}_K \cdot \mathrm{F} \cdot \mathrm{VOL\_cell}}) \cdot (I_{KATP} + I_{\mathrm{BK}} - 2 \cdot I_{\mathrm{NaK}} + I_{Kv} + I_{Kir} + I_{\mathrm{K\_leak}} + I_{\text{SMC-EC}}) + (\frac{10^{-12}}{\mathrm{VOL\_cell}}) \cdot J_{\mathrm{NaCl\_K}} \quad \text{(S86)}$$

$$\frac{dCa_{\mathrm{in}}}{dt} = -\frac{10^{-6}}{z_{\mathrm{Ca}}\, F\, \mathrm{VOL}_{\mathrm{cell}}} \left(I_{\mathrm{CaV}} + I_{\mathrm{Ca,leak}}\right) + \frac{10^{-6}}{F\, \mathrm{VOL}_{\mathrm{cell}}} \left(I_{\mathrm{PMCA}} - I_{\mathrm{NCX}}\right) + \frac{10^{-9}}{\mathrm{VOL}_{\mathrm{cell}}} \left(J_{\mathrm{SERCA}} + J_{\mathrm{Ca}_{\mathrm{m_i}} \to \mathrm{cyt,ALL}} + J_{\mathrm{Ca_M} \to \mathrm{cyt}}\right) - \left(k_{\mathrm{BUF,on}}\, Ca_{\mathrm{in}}\, (\mathrm{BUF}_T - \mathrm{BUF}_1) - k_{\mathrm{BUF,off}}\, \mathrm{BUF}_1\right) \quad \text{(S87)}$$

$$\frac{dCa_{WSTM}}{dt} = \frac{10^{-9}}{\mathrm{VOL}_{WSTM}} \cdot (J_{Ca\_TRPC6} - J_{Ca_{\mathrm{WSTM}}\text{-}cyt}) - (\mathrm{K}_{\mathrm{BUF\_on}} \cdot Ca_{WSTM} \cdot (\mathrm{BUF}_{M_T} - BUF_M) + \mathrm{K}_{\mathrm{BUF\_off}} \cdot BUF_M) \quad \text{(S88)}$$

$$\frac{dCa_{\text{BK-Glu}}}{dt} = \frac{10^{-9}}{\mathrm{VOL}_{BK-Glu}} \cdot (-J_{Ca_{\text{BK-Glu}}\text{-}cyt}) + (\frac{10^{-6}}{\mathrm{z}_{Ca} \cdot \mathrm{F} \cdot \mathrm{VOL}_{BK-Glu}})(I_{\mathrm{Ca_{NMDA}}}) \quad \text{(S89)}$$

$$\frac{dv_{EC}}{dt} = -\frac{10^{-9}}{\mathrm{C}_{EC}} \cdot (I_{Kir_{EC}} - I_{\text{SMC-EC}} + I_{EC-EC}) \quad \text{(S90)}$$

SM 2: Arteriolar Mechanobiological model

The contraction mechanism of SMCs is modeled using four-state Michaelis-Menten kinetics proposed by [12] to describe the different states of actin-myosin interaction. This model incorporates the different states of cross-

Table S19: Global electrophysiological model parameters

**Parameter**	**Value and Unit**	**Parameter**	**Value and Unit**
τ_{mct}	1–100 s	F	96,485 $\mathrm{C\,mol^{-1}}$
R	8.314 $\mathrm{J\,mol^{-1}\,K^{-1}}$	T	310 K
β	$\frac{1}{RT/F} = 37.45\ \mathrm{mV^{-1}}$	$\mathrm{C_m}$	2.5×10^{-11} F
$\mathrm{VOL_{cell}}$	1×10^{-12} L	$\mathrm{VOL_{WSTM}}$	6.3×10^{-17} L
$\mathrm{VOL_{Glu\text{-}BK}}$	2.6×10^{-17} L	$\mathrm{z_{Cl}}$	-1
$\mathrm{z_{Na}}$	+1	$\mathrm{z_K}$	+1
$\mathrm{z_{Ca}}$	+2	$\mathrm{K_{ex}}$	4 $\mathrm{mmol\,L^{-1}}$
$\mathrm{Na_{ex}}$	143 $\mathrm{mmol\,L^{-1}}$	$\mathrm{Ca_{ex}}$	1.6 $\mathrm{mmol\,L^{-1}}$
$\mathrm{Cl_{ex}}$	127 $\mathrm{mmol\,L^{-1}}$	$\mathrm{C_{EC}}$	8×10^{-12} F

bridge cycling, including: free nonphosphorylated cross bridges (M), free phosphorylated cross bridges (Mp), attached phosphorylated cross bridges (AMp), and attached dephosphorylated latch bridges (AM). A system of ODEs describes the temporal evolution of these states, with rate constants defining the transitions. The Ca^{2+} dependency of actomyosin interactions, along with the effects of NO/cGMP/PKG signaling, was incorporated by modulating these rate constants. By conserving the total myosin across all states, the model provides a robust framework for simulating SMC constriction dynamics.

$$\frac{dM_p}{dt} = \mathrm{K}_4 \cdot AM_p + K_1 \cdot M - (K_2 + \mathrm{K}_3) \cdot M_p \tag{S91}$$

$$\frac{dAM_p}{dt} = \mathrm{K}_3 \cdot M_p + K_6 \cdot AM - (\mathrm{K}_4 + K_5) \cdot AM_p \tag{S92}$$

$$\frac{dAM}{dt} = K_5 \cdot AM_p - (\mathrm{K}_7 + K_6) \cdot AM \tag{S93}$$

$$M = 1 - AM - AM_p - M_p \tag{S94}$$

$$K_1(Ca_{\mathrm{in}}) = \gamma_{\mathrm{cross}} \cdot Ca_{\mathrm{in}}^{\mathrm{n_{cross}}}, \qquad K_6(Ca_{\mathrm{in}}) = K_1 \tag{S95}$$

$$K_2(NO) = \alpha_{\mathrm{mlcp\_b}} \cdot \mathrm{k_{mlcp\_b}} + \alpha_{\mathrm{mlcp\_c}} \cdot \mathrm{k_{mlcp\_c}} \cdot NO, \qquad K_5 = K_2 \tag{S96}$$

$$F_r(AM_p, AM) = (AM_p + AM) \tag{S97}$$

$$E_{\mathrm{Pass}}(WS) = \frac{\mathrm{E_{passive}} \cdot WS}{\mathrm{K_{pass}}} + \mathrm{E_{passive}} \tag{S98}$$

$$E(WS, F_r) = E_{\mathrm{Pass}} + \mathrm{RC} \cdot F_r \cdot (\mathrm{E_{active}} - E_{\mathrm{Pass}}) \tag{S99}$$

$$R_0 = \mathrm{R0_{passive}} + \mathrm{RC} \cdot F_r \cdot (\alpha_R - 1) \cdot \mathrm{R0_{passive}} \tag{S100}$$

$$\frac{dR}{dt} = (\frac{\mathrm{R0_{passive}}}{\eta}) \cdot (\frac{R \cdot P(Pa)}{h} - E \cdot \frac{(R - R_0)}{R_0}) \tag{S101}$$

where RC refers to relative contractility of each PA segment. The fraction of attached cross-bridges, F_r, varies between 0 and 1, modulating the active contribution to the total Young's modulus. $\mathrm{h} = 0.1 \cdot \mathrm{R0_{passive}}$, and $\mathrm{R0_{passive}} = 0.9 \cdot \mathrm{Rmax_{active}}$. For a detailed explanation, readers can refer to [13].

Table S20: Mechanobiological model parameters[13, 14]

**Parameter**	**Value and Unit**	**Parameter**	**Value and Unit**
γ_{cross}	35 µmol^{-3}/s	$\mathrm{n_{cross}}$	3
$\alpha_{\mathrm{mlcp\_b}}$	58.1395	$\mathrm{k_{mlcp\_b}}$	0.0086 s^{-1}
$\alpha_{\mathrm{mlcp\_c}}$	58.1395	$\mathrm{k_{mlcp\_c}}$	0.0327 s^{-1}
$\mathrm{E_{passive}}$	66×10^3 Pa	$\mathrm{K_{pass}}$	22–42 $\times 10^3$
$\mathrm{E_{active}}$	433×10^3 Pa	RC	0.9–1.1
α_R	0.6	K_3	2.4 s^{-1}
K_4	0.1 s^{-1}	K_7	0.1 s^{-1}
η	1×10^4 Pa·s		

References

[1] Arthur Karlin. Membrane potential and ca2+ concentration dependence on pressure and vasoactive agents in arterial smooth muscle: A model. *Journal of General Physiology*, 146(1):79–96, 2015.

[2] Elshin J Mathias, Allanah Kenny, Michael J Plank, and Tim David. Integrated models of neurovascular coupling and bold signals: Responses for varying neural activations. *Neuroimage*, 174:69–86, 2018.

[3] Osama F Harraz, David Hill-Eubanks, and Mark T Nelson. Pip2: a critical regulator of vascular ion channels hiding in plain sight. *Proceedings of the National Academy of Sciences*, 117(34):20378–20389, 2020.

[4] Albert L Gonzales, Ying Yang, Michelle N Sullivan, Lindsey Sanders, Fabrice Dabertrand, David C Hill-Eubanks, Mark T Nelson, and Scott Earley. A plcγ1-dependent, force-sensitive signaling network in the myogenic constriction of cerebral arteries. *Science Signaling*, 7(327):ra49–ra49, 2014.

[5] Arash Moshkforoush, Baarbod Ashenagar, Osama F Harraz, Fabrice Dabertrand, Thomas A Longden, Mark T Nelson, and Nikolaos M Tsoukias. The capillary kir channel as sensor and amplifier of neuronal signals: Modeling insights on k+-mediated neurovascular communication. *Proceedings of the National Academy of Sciences*, 117(28):16626–16637, 2020.

[6] Masayo Koide, Arash Moshkforoush, Nikolaos M Tsoukias, David C Hill-Eubanks, George C Wellman, Mark T Nelson, and Fabrice Dabertrand. The yin and yang of kv channels in cerebral small vessel pathologies. *Microcirculation*, 25(1):e12436, 2018.

[7] Maniselvan Kuppusamy, Matteo Ottolini, and Swapnil K Sonkusare. Role of trp ion channels in cerebral circulation and neurovascular communication. *Neuroscience letters*, 765:136258, 2021.

[8] Qian Wang, M Dennis Leo, Damodaran Narayanan, Korah P Kuruvilla, and Jonathan H Jaggar. Local coupling of trpc6 to ano1/tmem16a channels in smooth muscle cells amplifies vasoconstriction in cerebral arteries. *American Journal of Physiology-Cell Physiology*, 310(11):C1001–C1009, 2016.

[9] Ashwini Hariharan, Colin D Robertson, Daniela CG Garcia, and Thomas A Longden. Brain capillary pericytes are metabolic sentinels that control blood flow through a katp channel-dependent energy switch. *Cell Reports*, 41(13):111872, 2022.

[10] Ashwini Hariharan, Nick Weir, Colin Robertson, Liqun He, Christer Betsholtz, and Thomas A Longden. The ion channel and gpcr toolkit of brain capillary pericytes. *Frontiers in Cellular Neuroscience*, 14:423, 2020.

[11] Maria Sancho, Nicholas R Klug, Amreen Mughal, Masayo Koide, Saul Huerta de la Cruz, Thomas J Heppner, Adrian D Bonev, David Hill-Eubanks, and Mark T Nelson. Adenosine signaling activates atp-sensitive k+ channels in endothelial cells and pericytes in cns capillaries. *Science Signaling*, 15(727):eabl5405, 2022.

[12] Chi-Ming Hai and Richard A Murphy. Cross-bridge phosphorylation and regulation of latch state in smooth muscle. *American Journal of Physiology-Cell Physiology*, 254(1):C99–C106, 1988.

[13] Katharina Dormanns. *Computational models of neurovascular coupling.* Phd thesis, University of Canterbury, Christchurch, New Zealand, 2015.

[14] Allanah Kenny, Constantine Zakkaroff, Michael J Plank, and Tim David. Massively parallel simulations of neurovascular coupling with extracellular diffusion. *Journal of computational science*, 24:116–124, 2018.
